# Comprehensive analysis of both long and short read transcriptomes of a clonal and a seed-propagated model species reveal the prerequisites for transcriptional activation of autonomous and non-autonomous transposons in plants

**DOI:** 10.1186/s13100-022-00271-5

**Published:** 2022-05-12

**Authors:** Ting-Hsuan Chen, Christopher Winefield

**Affiliations:** 1 Department of Wine, Food, and Molecular Biosciences, Lincoln University, Lincoln, 7647 New Zealand; 2 Present address: The New Zealand Institute for Plant and Food Research Ltd, Lincoln, 7608 New Zealand

**Keywords:** Transposable element, Stress treatment, Transcription, Differential expression, Alternative splicing, Nanopore sequencing

## Abstract

**Background:**

Transposable element (TE) transcription is a precursor to its mobilisation in host genomes. However, the characteristics of expressed TE loci, the identification of self-competent transposon loci contributing to new insertions, and the genomic conditions permitting their mobilisation remain largely unknown.

**Results:**

Using *Vitis vinifera* embryogenic callus, we explored the impact of biotic stressors on transposon transcription through the exposure of the callus to live cultures of an endemic grapevine yeast, *Hanseniaspora uvarum*. We found that only 1.7–2.5% of total annotated TE loci were transcribed, of which 5–10% of these were full-length, and the expressed TE loci exhibited a strong location bias towards expressed genes. These trends in transposon transcription were also observed in RNA-seq data from *Arabidopsis thaliana* wild-type plants but not in epigenetically compromised Arabidopsis *ddm1* mutants. Moreover, differentially expressed TE loci in the grapevine tended to share expression patterns with co-localised differentially expressed genes. Utilising nanopore cDNA sequencing, we found a strong correlation between the inclusion of intronic TEs in gene transcripts and the presence of premature termination codons in these transcripts. Finally, we identified low levels of full-length transcripts deriving from structurally intact TE loci in the grapevine model.

**Conclusion:**

Our observations in two disparate plant models representing clonally and seed propagated plant species reveal a closely connected transcriptional relationship between TEs and co-localised genes, particularly when epigenetic silencing is not compromised. We found that the stress treatment alone was insufficient to induce large-scale full-length transcription from structurally intact TE loci, a necessity for non-autonomous and autonomous mobilisation.

**Supplementary Information:**

The online version contains supplementary material available at 10.1186/s13100-022-00271-5.

## Background

A substantial proportion of eukaryotic genomes are comprised of transposable elements (TEs) [1, 2]. These elements have long been considered unavoidable and deleterious components of host genomes, being described variously as parasitic or junk DNA. However, there has been a recent resurgence in interest in the roles of TEs, in particular those associated with genome and organism evolution [2–6], as well as environmental adaptation [7–10].

The first committed step in the life cycle of a transposon is the transcription of a fully competent, or autonomous, copy of a given TE. De-novo transcription of these TEs is an absolute requirement for TE mobilisation irrespective of TE type. Autonomous transposition of type I retrotransposons is dependent on reverse transcription of transcripts derived from intact retrotransposon loci, while autonomous mobilisation of type II DNA transposons requires transcription of a functional transposase enzyme that facilitates the transposase-mediated excision of elements from genomic loci containing the corresponding inverted repeat sequences [5, 11]. Non-autonomous TE elements, such as terminal-repeat retrotransposon in miniature (TRIMs) or miniature inverted-repeat transposable elements (MITEs), cannot mobilise without the aid of functional protein elements provided by closely related autonomous elements. In addition to possible transposition, transcription of TE loci, containing either intact or fragmented TEs, can impact normal activity of neighbouring genes without generating new TE insertions by interfering with normal gene splicing and epigenetic silencing of these loci [12, 13].

Eukaryote epigenetic regulatory networks are known to target TE activity through DNA methylation, histone modification and small RNA based mechanisms [14–16]. Consequently, the complex interplay between initiation of TE transcription and epigenetic suppression sets the scene for transposon mobility and the consequential impact that such mobility has on genome function.

Despite the multitude of TE-focused silencing mechanisms, TEs remain able to mobilise and contribute to genetic diversity across evolutionary time. It is unclear both how TEs escape from these silencing systems and at what rates to increase their prevalence in many wild-type eukaryote genomes. Reactivation of TE transcription and mobilisation has often been observed in plants containing mutations that impair epigenetic silencing [9, 17–20]. In wild-type backgrounds that are not impaired in epigenetic silencing, it has been widely reported that the establishment of plant tissue culture or stress treatments can result in transcriptional activation and mobilisation of TEs [21–25]. Investigation of the location of new TE insertions has revealed an insertion bias of low-copy-number DNA transposons and LTR-retrotransposons towards the gene-rich region in maize [26, 27], rice [28–30], and *Arabidopsis thaliana* [9]. Mobilisation of elements, as mentioned above, relies on autonomous TEs, whose transcriptional activity and production of encoded proteins is an essential precursor of transposition. However, which of these autonomous elements is key often remain elusive.

Due to the highly repetitive nature of TE sequence in genomes, most literature on TE transcriptional regulation reports this transcriptional activity at the family level [12]. Thus very little is known about the number and location of autonomous TE loci that contribute to observed pool of TE transcripts. Attribution of sequencing reads to specific autonomous loci has been practically impossible. This inability to effectively determine transcriptional activity at individual TE loci hampers the study of the transcriptional regulation of these loci and, therefore, regulation of the mobilisation of autonomous TE elements and partnered non-autonomous loci throughout the genome.

To address these questions, we chose to use *Vitis vinifera,* and specifically, embryogenic callus, as a model system to thoroughly interrogate exposure of these cultures to biotic stressors with respect to the impact of such stress on the transcription of TE loci. Grapevine, being largely clonally propagated, is likely to have epigenetic landscapes that favour transposon activity due to the lack of epigenetic reprogramming observed in sexually propagated plant species [31, 32]. This system provides a useful counterpoint to sexually reproducing systems, such as Arabidopsis, where the epigenetic landscape is reset at each generation [33].

The combination of a small (~ 0.5 Gb), well annotated genome and transposon landscape [34–36], alongside a potentially favourable epigenetic landscape, presented grapevine as a useful model system to study transposon activation. The biotic stressor we chose to expose callus to was live *Hanseniaspora uvarum* cultures. This organism is a yeast that is commonly found in vineyards [37–39] and that we have previously shown to activate TE transcription in embryogenic callus [21]. To validate our findings in grapevine, we also explored the transcriptional patterns of TE loci in Arabidopsis using published *A. thaliana* RNA-sequencing (RNA-seq) datasets, including wild-type [40], and *ddm1* mutants [17]. The *ddm1* mutant is impaired in epigenetic silencing of heterochromatic TEs [18] and shows high levels of TE mobilisation. Short-read RNA-seg data is being augmented by the RNA-seq data produced by longer read sequencing technology. While long-read sequencing technology delivers obvious benefits for analysis of TE activity in genomes [32], the availability of large numbers of short-read sequence datasets still provides a need for TE-analysis pipelines for these datasets. Short-read sequence data has a number of well documented shortcomings [41]. This has resulted in most analyses focusing on family level analysis, abandoning attempts to use these data to identify transcription from specific loci within genomes [12]. To circumvent these commonly encountered issues with the mapping of short-read RNA-seq datasets to repetitive sequences, we utilised multiple existing tools to collectively identify ‘TE expression candidates’. We define these as annotated TE loci that were mappable by both unique- or multi-mapping short sequencing reads and thus potentially expressed (see Methods). After defining the set of TE loci from which identified transcripts can originate we further delved into the nature of these loci and resolved uniquely mapped loci and un-mappable loci due to the lack of identifiable nucleotide variation within and between expression candidates. Our analyses reveal that the transcriptional activity of TE expression candidates was tightly linked with expressed genes in terms of co-location of expressed genes and TE insertions and with respect to the temporal pattern of expression of the co-localised genes. In order to augment and validate our analysis pipeline for short-read sequence data, we explored the utility of long-read sequence technology to detect transcription of potentially autonomous elements and thus map their location in a genome, using long-read Oxford Nanopore cDNA sequencing in the grapevine system.

## Results

### Most TE expression candidates are fragmented and polymorphic TE loci

**Fig. 1:**
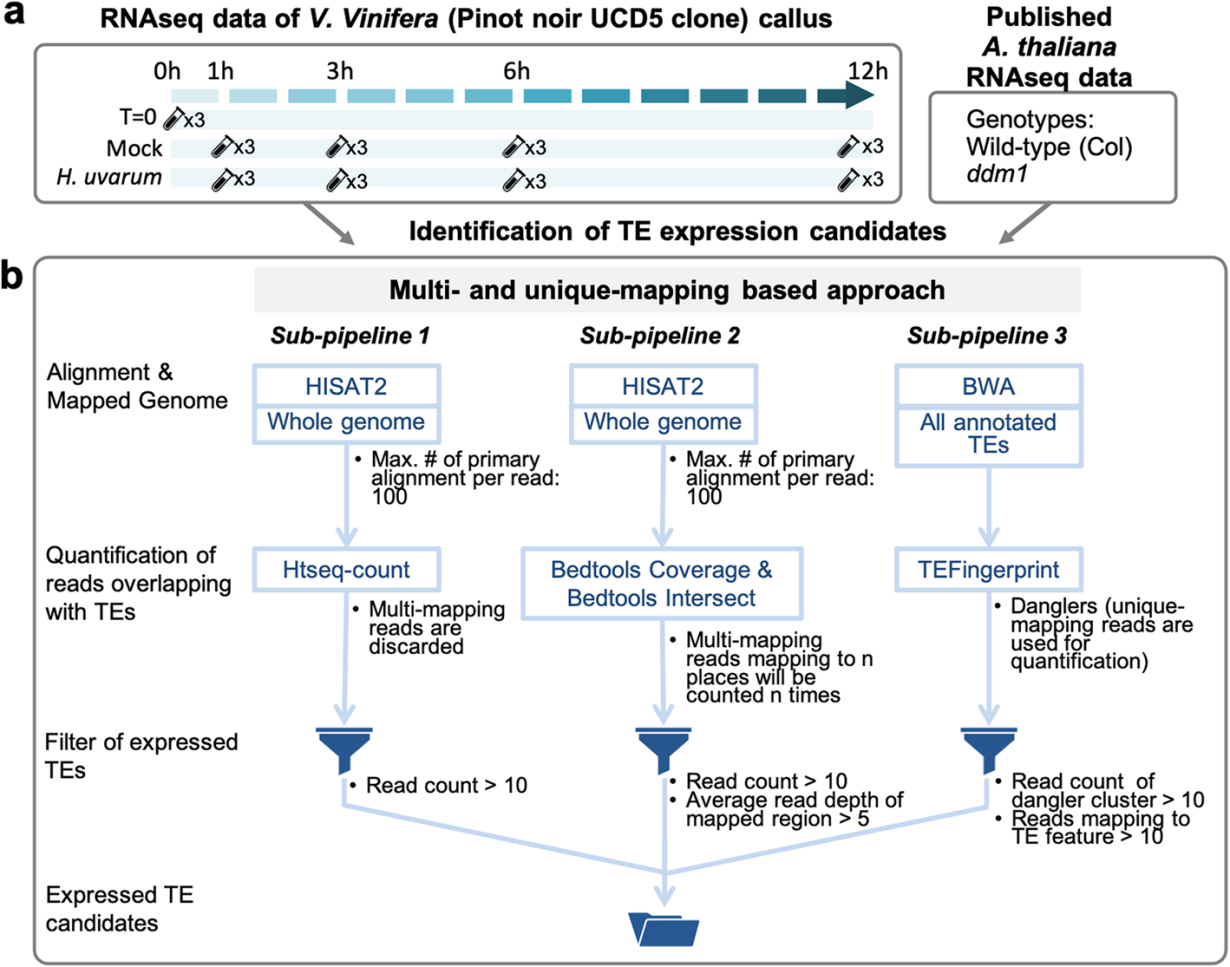
Short-read RNA-seq data sources and the analysis workflow to identify expressed TE loci. **a** A schematic of the sampling regime for the generation of grapevine short-read RNA-seq data. The grapevine RNA-seq data was derived from the *V. vinifera* embryogenic callus subjected to time-series stress treatment (each with three biological replicates). Published RNA-seq data of *Arabidopsis thaliana* seedlings of wild-type [40] and *ddm1* [17] were also used in the analysis pipeline. **b** Computational workflow to identify TE expression candidates. The first and second sub-pipelines apply HISAT2 [42] for the alignment of sequencing reads against the reference genome and then use htseq-count [43] and Bedtools toolset [44], respectively, to quantify reads overlapping with TEs. While htseq-count only adopts unique-mapping reads, Bedtools incorporates both unique- and multi-mapping reads. The third sub-pipeline uses BWA [45] to align reads against TE sequences, after which the mates of TE-mapped reads would be fed into TEFingerprint [46] for mapping against the reference genome to capture danglers. The three sets of TEs passing through the filtering steps are joined together as a pool of expression candidates (see Methods, Fig. S1 and additional file 1 for detailed descriptions of definitions and thresholds)

Following the system established by Lizamore [21] to activate TE transcription, *V. vinifera* (Pinot noir UCD5 clone) embryogenic callus cultures were subjected to either a mock treatment (hereinafter Vv_Mock; which included vigorous shaking that was expected to mount a wounding response in this tissue; see Methods) or mock treatment plus *H. uvarum* live cultures (hereinafter Vv_Yeast). Samples were collected after exposure at four time points, with an untreated embryogenic callus culture included as a common time zero (Vv_T = 0) for both Vv_Mock and Vv_Yeast treatments (see Methods and Fig. 1a for experimental schematic). Triplicated treatments of embryogenic callus were carried out, RNA-seq libraries prepared and sequence data generated as outlined in Methods. All TE loci potentially transcribed were identified by our analysis pipeline constituted by existing tools (see Methods and Fig. 1b). To provide data to validate our analysis pipeline we retrieved the RNA-seq data of Arabidopsis wild type (*Col*) [40] and *ddm1* mutant [17] and subjected this to the same analysis pipeline. The pipeline produced three sets of TE loci that passed our arbitrary expression threshold (see Methods and Fig. 1b) and were united as a pool of expression candidates. ‘Trackable’ expression candidates were considered TE loci in which mapped reads possessed a unique polymorphism such as a single nucleotide variant (SNV) or a small insertion or deletion (INDEL). ‘Un-trackable’ expression candidates represent a collection of TE loci for which the sequencing reads obtained show no sequence divergence and that all TE loci in this set have to be considered as potentially transcribed and likely represent TEs that have recently mobilised [47]*.*

**Fig. 2:**
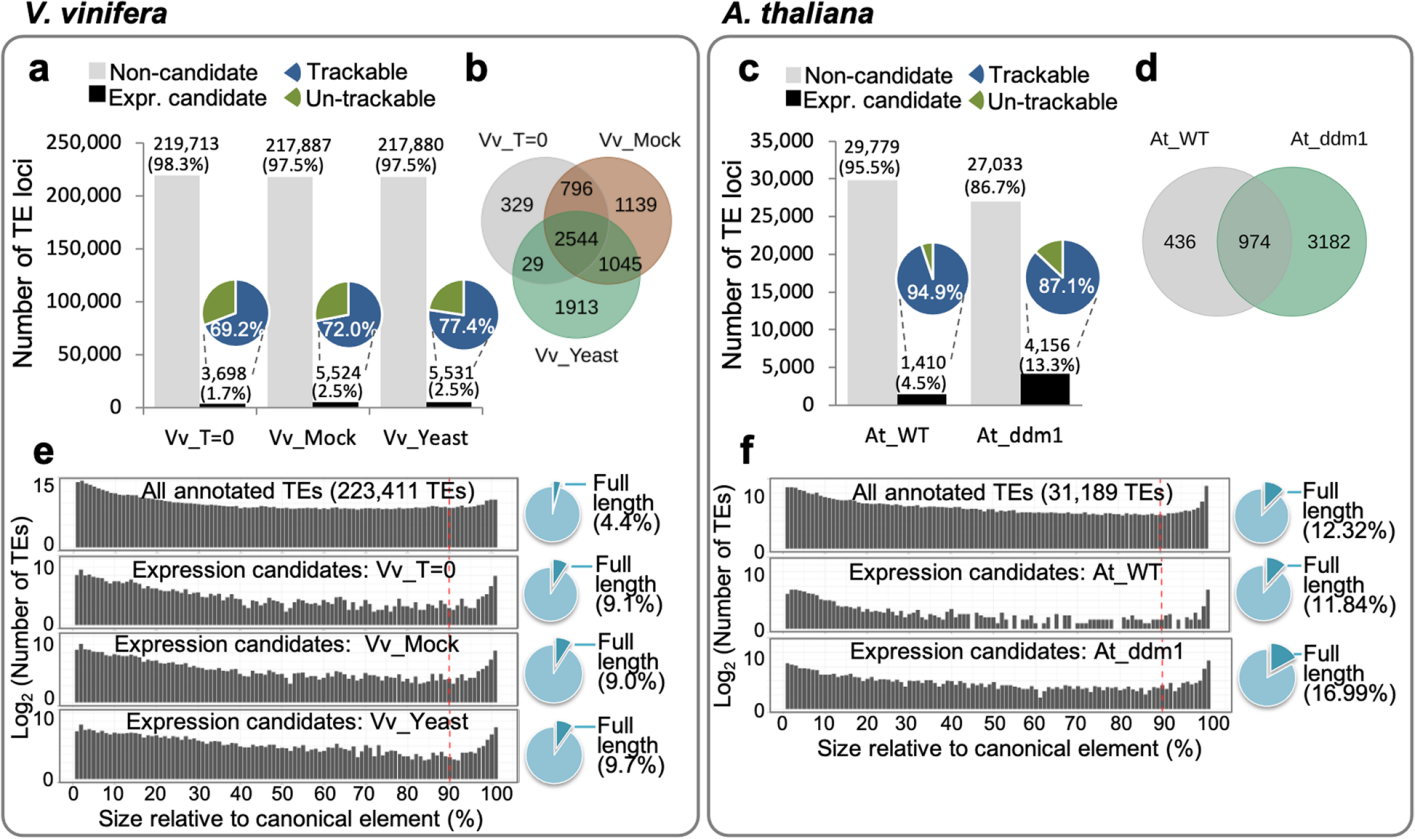
Identification of TE expression candidates in *V. vinifera* embryogenic callus and *A. thaliana* seedlings. **a, c** Bar charts demonstrating the abundance of TE expression candidate loci identified in different experimental conditions or genotypes of the *V. vinifera* (**a**) and *A. thaliana* (**c**) systems, with pie charts showing the proportion of trackable/un-trackable loci of TE expression candidates. **b, d** Venn diagrams showing different sets of TE expression candidates in the *V. vinifera* (**b**) and *A. thaliana* (**d**) systems. **e, f** Histograms demonstrating the length integrity of all annotated TE loci and TE expression candidates, and pie charts showing the proportion of full-length TE loci among all annotated TE loci and TE expression candidates, in the *V. vinifera* (**e**) and *A. thaliana* (**f**) systems. Trackable elements refer to those loci that has sufficient sequence divergence to allow reads to be unambiguously mapped to a specific genomic locus. Un-trackable elements refer to loci that have reads that multimap to many loci due to lack of any sequence divergence at these loci, the assumption being these represent recent insertions that have not yet accumulated sequence variation

In *V. vinifera* embryogenic callus, our analysis workflow identified 3698 (1.6%), 5524 (2.5%), and 5531 (2.5%) of a total of 223,411 annotated TE loci as expression candidates in Vv_T = 0, Vv_Mock, and Vv_Yeast samples, respectively (Fig. 2a). Of the total number of loci containing annotated TE sequence in the *Vitis* genome, 75–87% were effectively excluded as having no evidence of transcription and a further 11–22% of annotated TE loci fell below our expression threshold due to insufficient support in terms of numbers of mapped reads (Additional file 2: Fig. S2). In each of the tested conditions (Vv_T = 0, Vv_Mock and Vv_Yeast), different sets of expression candidates were identified (Fig. 2b). The increase in number of expression candidates uniquely found in Vv_Mock (1139 TE loci; Fig. 2b) and Vv_Yeast (1913 TE loci; Fig. 2b) compared to our time 0 control (Vv_T = 0; 329 TE loci; Fig. 2b) indicates heightened transcriptional activation of TE loci due to wound and biotic stress treatments.

Similarly, in the Arabidopsis RNA-seq datasets, the wild-type dataset revealed over 95% of the total 31,189 annotated Arabidopsis TE loci were excluded from the pool of expression candidates, leaving 1410 TE loci identified as expression candidates (Fig. 2c). However, in the mutant *ddm1* background, the number of expression candidates increased to 4156 TE loci (Fig. 2c), of which 3182 were transcribed TE loci compared to wild type (Fig. 2d). This marked difference in patterns of transcribed loci in the *ddm1* mutant likely reflected the deficiency in the epigenetic regulatory system in this mutant background leading to more transcriptionally permissive epigenetic landscape and activation of TEs [18].

Depending on the experimental condition or genotype in the *V. vinifera* and *A. thaliana* system, 69.2–77.4% and 87.1–94.9% of the expression candidates, respectively, contained unique-mapping reads, and were thus identified as trackable expression candidates in these two plant systems (Fig. 2a, c). Of the identified expression candidates, most where fragmented, with only 9–10% or 11–17% of the expression candidates in *V. vinifera* and *A. thaliana* system retaining > 90% integrity compared to the length of canonical elements (Fig. 2e, f). We termed TE loci with > 90% integrity in length ‘full-length’ TE loci.

The high proportion of trackable and fragmented TE loci in the pools of expression candidates in both systems indicate that the bulk of transcription of TEs observed in plants is likely derived from TEs which have both accumulated sequence polymorphisms and are fragmented, indicating that they likely derived from much older TE insertion events.

### The characteristics of TE expression candidates varies by TE family

The high proportion of fragmented elements triggered our interest in determining whether the degree of mutation accumulation within expressed TE families varied according to each family’s mobilisation history. We hypothesised that transcribed TE loci of more recently mobile families are expected to have less sequence divergence compared to those transcribed TE loci belonging to families with more ancient mobilisation events [48, 49]. Thus while there may not be a correlation between older versus newer insertions and transcription levels of TE-loci, younger elements may have a higher proportion of full-length and un-trackable loci.

**Fig. 3:**
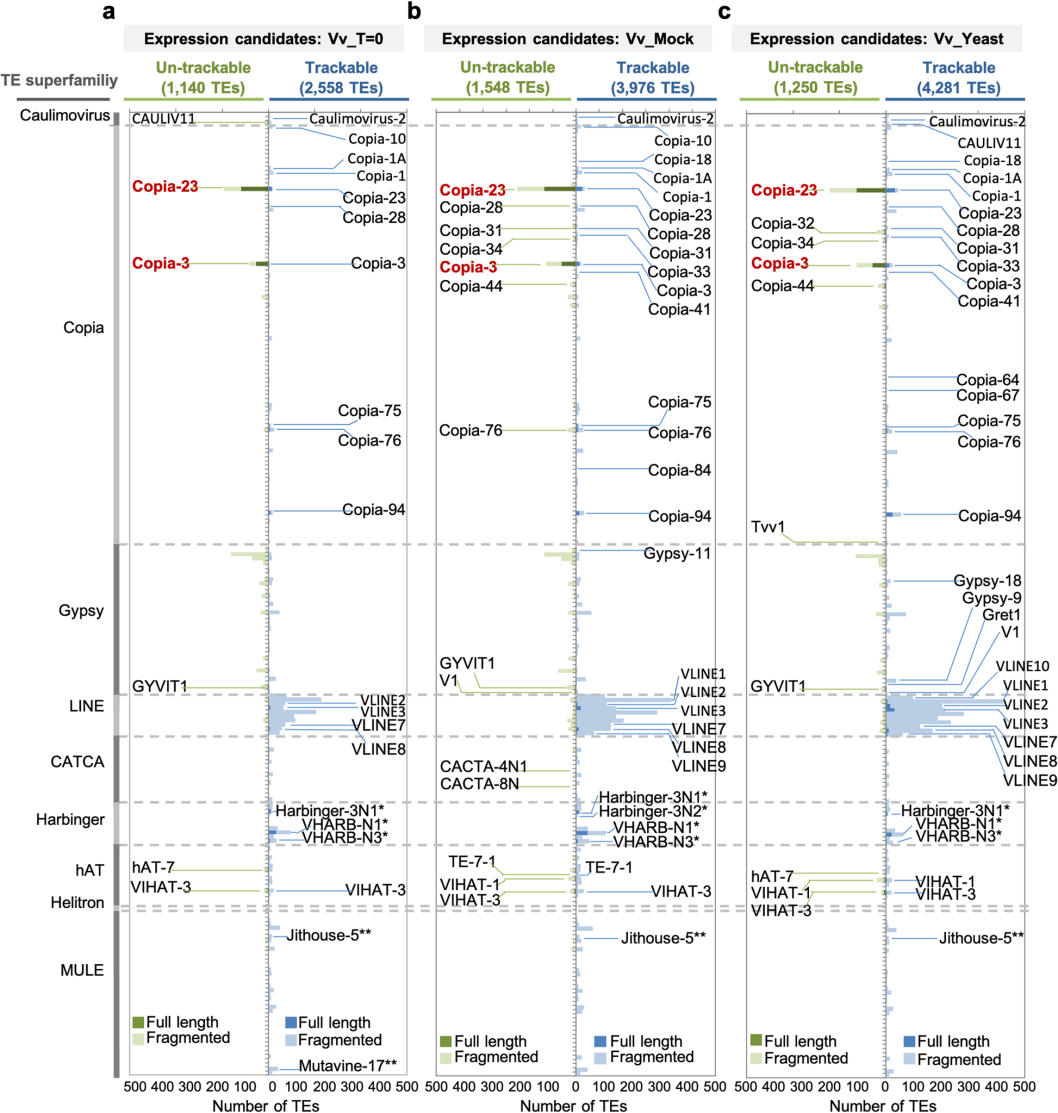
Transcriptionally active TE families in grapevine embryogenic callus. **a–c** Histograms demonstrating all expression candidates of Vv_T = 0 (**a**), Vv_Mock (**b**), and Vv_Yeast (**c**) categorised by families, distinctiveness and integrity. Each bar represents a TE family containing expression candidates. The expression candidates were then further grouped into un-trackable (green) and trackable (blue) candidates, those of which full-length were filled with either dark green or dark blue. TE families containing at least two full-length expression candidates of either group were indicated. Note that Harbinger families missing open reading frame (ORF) encoding transposase and MULE families lack of terminal inverted repeats (TIRs) in their canonical sequences are indicated

To test this assumption, we initially considered the grape control data (Vv_T = 0) and grouped grapevine expression candidates hierarchically by each family, degree of sequence divergence (i.e. trackable or un-trackable), and length (i.e. fragmented or full-length). Of the TE loci showing evidence of transcription 2558 (69%) of the total 3698 expression candidates were trackable, whereas the remaining 1140 expression candidates fell into the ‘un-trackable’ category (Fig. 3a). Among the 232 TE families, belonging to nine super-families, presented on the y-axis of Fig. 3a, 174 of these contained expression candidates in the control treatment, Vv_T = 0. Of the 174 families, 102 contained fewer than ten expression candidate loci, and a further 32 families contained 10 to 23 expression candidate loci (Additional file 3: Table S3). It was noticeable that 6 of the 174 TE families had more than 100 expression candidate loci; these families were Copia-23 (211 expression candidates), Gypsy-12 (175 expression candidates), VLINE1 (245 expression candidates), VLINE4 (211 expression candidates), VLINE5 (117 expression candidates) and VLINE6 (139 expression candidates). However, of these, the expression candidate loci in 5 of the families were mostly fragmented and trackable TE sequences (Additional file 3: Table S3), indicating that only a small number of families contribute the bulk of putatively transcribed loci, while the bulk of the expressed TE families have few transcriptionally active TE loci. In fact, half of the expressed TE families (87 of the 174 expressed families) possessed only trackable expression candidates, and a further 74 TE families had only 1 to 20 un-trackable expression candidates, most of which are fragmented.

These observations demonstrates that the vast majority of the transcriptionally active loci across TE families are fragmented and could be identified by polymorphisms (e.g. SNVs and INDELs). In contrast, Copia-23 and Copia-3 families were over-represented with expression candidates that were both un-trackable and potentially full-length. These findings were concordant with the observations in Vv_mock (Fig. 3b) and Vv_Yeast (Fig. 3c). In the treatment series, we observed larger numbers of TE families containing transcribed TE loci, however, as observed in the control Vv_T = 0, most of the expressed families possess a majority of loci that are fragmented. Copia-3 and Copia-23 remained the two families possessing the largest numbers of full-length un-trackable expression candidates (Additional file 3: Table S3).

A closer interrogation of the sequences of the canonical Copia-3 element and the 90 annotated full-length Copia-3 elements shows a condensed phylogenetic cluster mostly comprised of structurally intact (and thus potentially autonomous) Copia-3 indicated by the presence of LTRs flanking an intact INT domain (Additional file 2: Fig. S3; also see Methods). This cluster included 26 sequences, 19 of which were structurally intact un-trackable expression candidates with over 90% read coverage of the annotated INT domain, and a further 4 of which were intact trackable Copia-3 with a nearly complete transcription of INT (see Methods).

A similar analysis of the Copia-23 family, comparing the canonical sequence of Copia-23 and the 220 full-length Copia-23 sequences, revealed that the 11 un-trackable and four trackable structurally intact candidates with nearly complete transcription across INT were scattered in 5 broom-like unresolved clusters. These clusters were densely packed with other un-trackable candidates that had either lost intact LTRs or lacked full INT coverage (Additional file 2: Fig. S4). These compact clades with short branches were distinguished from the sequences of unexpressed full-length Copia-23.

To test the contribution of the full-length Copia-3 loci to the pool of Copia-3-related transcripts, reads mapping to all Copia-3 expression candidates were categorised into four groups by whether they mapped to; full-length trackable, full-length un-trackable, fragmented trackable and fragmented un-trackable candidates. This analysis revealed that each category contained reads shared with one or more categories, irrespective of treatments and treatment time-point (Additional file 2: Fig. S5). Nonetheless, each category obtained a unique subset of reads that only mapped to one of the four groups (pairwise combination of full-length/fragmented and trackable/un-trackable) of expression candidates, meaning no one group was able to represent the whole collection of Copia-3 transcripts. The same analysis was applied to reads mapping to all Copia-23 expression candidates. This analysis also demonstrated reads shared across different categories and those reads unique to a single category (Additional file 2: Fig. S6).

**Fig. 4:**
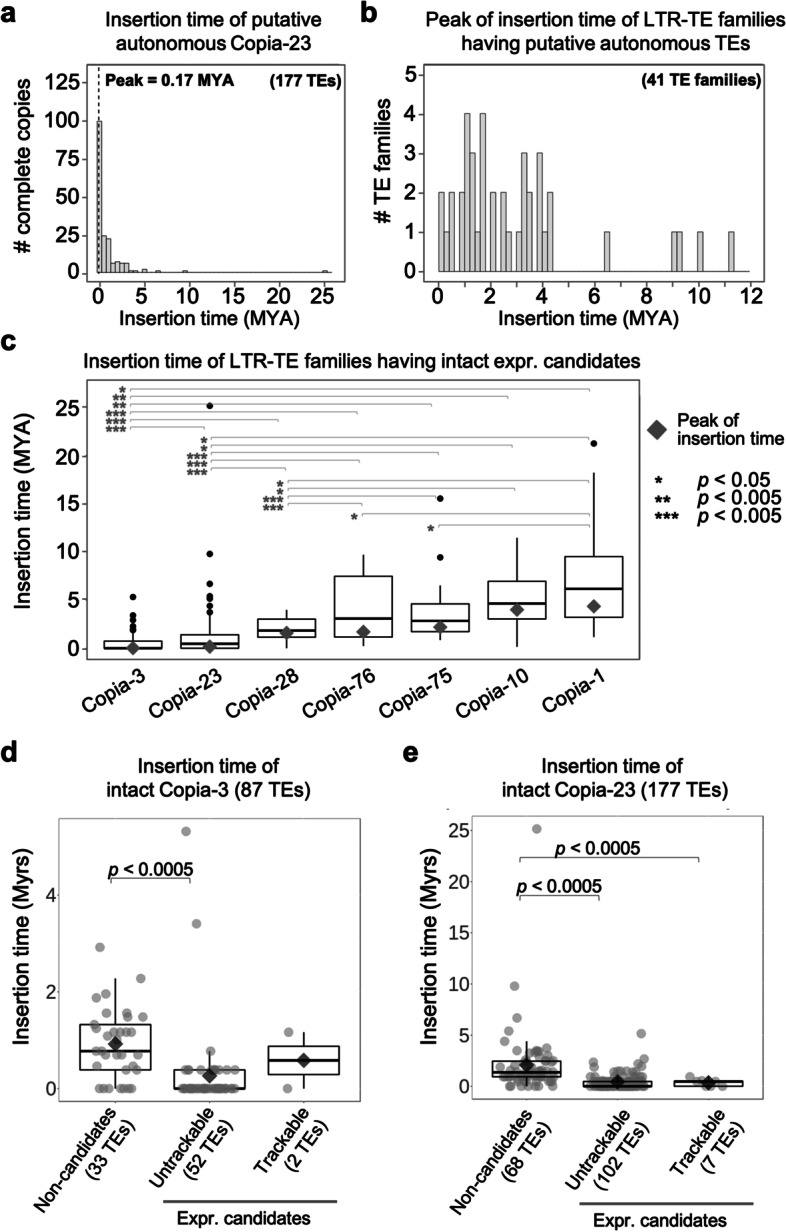
Insertion dates of LTR-TE families containing complete copies. **a** Distribution of the insertion dates for 177 complete copies of the Copia-23 family. The peak of amplification (0.17 MYA) is indicated as the dashed line. **b** Distribution of the insertion dates using 41 LTR-TE families with at least 10 complete copies. **c** Comparison of the transposition burst time among LTR-TE families containing full-length expression candidates. The families were ordered by the peak of insertion time (grey diamonds). The asterisks denote a significant level of t-test for the mean of insertion time as indicated. **d, e** Comparison of the insertion time of all annotated structurally intact Copia-3 (**d**) and Copia-23 (**e**) loci grouped into three categories: non-candidates, un-trackable expression candidates, and trackable expression candidates

Copia-3 and Copia-23 belong to LTR-retrotransposon super family (LTR-TEs). This type of TE is characterised by the presence of identical long terminal repeat (LTR) at either end of the element. The pair of LTRs gradually degrades over time, accumulating increasing numbers of mutations across time; therefore, it has been believed that the more diverse a pair of LTR sequences are, the more time that has passed since insertion [49, 50]. To test whether Copia-3 and Copia-23 were active more recently than other LTR-TEs, we analysed the divergence of each pair of LTRs, then estimating the insertion time of each structurally intact TE loci. The insertion dates of the 87 and 177 Copia-3 and Copia-23 intact elements, respectively, were estimated to peak approximately 0.02 and 0.017 million years ago (MYA), respectively (Fig. 4a, b; Additional file 4: Table S3). Peak insertion times of the other 39 LTR-TE families with at least ten intact copies were analysed in the same way (Fig. 4b; Additional file 4: Table S3). Most LTR-TE families experienced bursts no longer than 4.5 million years ago (MYA). Note that Copia-3 and Copia-23 were the most recently active LTR-TE families (Additional file 4: Table S3). Comparison of the peak insertion time of Copia-3, Copia-23 and the other 5 Copia families, which obtained trackable full-length candidates across all treatments but lacked un-trackable full-length candidates, showed that Copia-3 and Copia-23 experienced their bursts of activity more recently than these five other families (Fig. 4c). In addition, the average insertion time of intact un-trackable candidates is significantly more recent than the non-candidates of Copia-3 and Copia-23 (Fig. 4d, e).

Together, these results suggest that LTR-TE families that show evidence of the most recent mobilisation burst in evolutionary time may not necessarily be TE families possessing the largest number of expression candidate loci. Instead, recently mobile families that contain currently active element loci are likely typified by collections of loci that are structurally intact and where RNA-seq reads multi-map across these conserved sequences.

### TE expression candidates are not randomly distributed in the genome

**Fig. 5:**
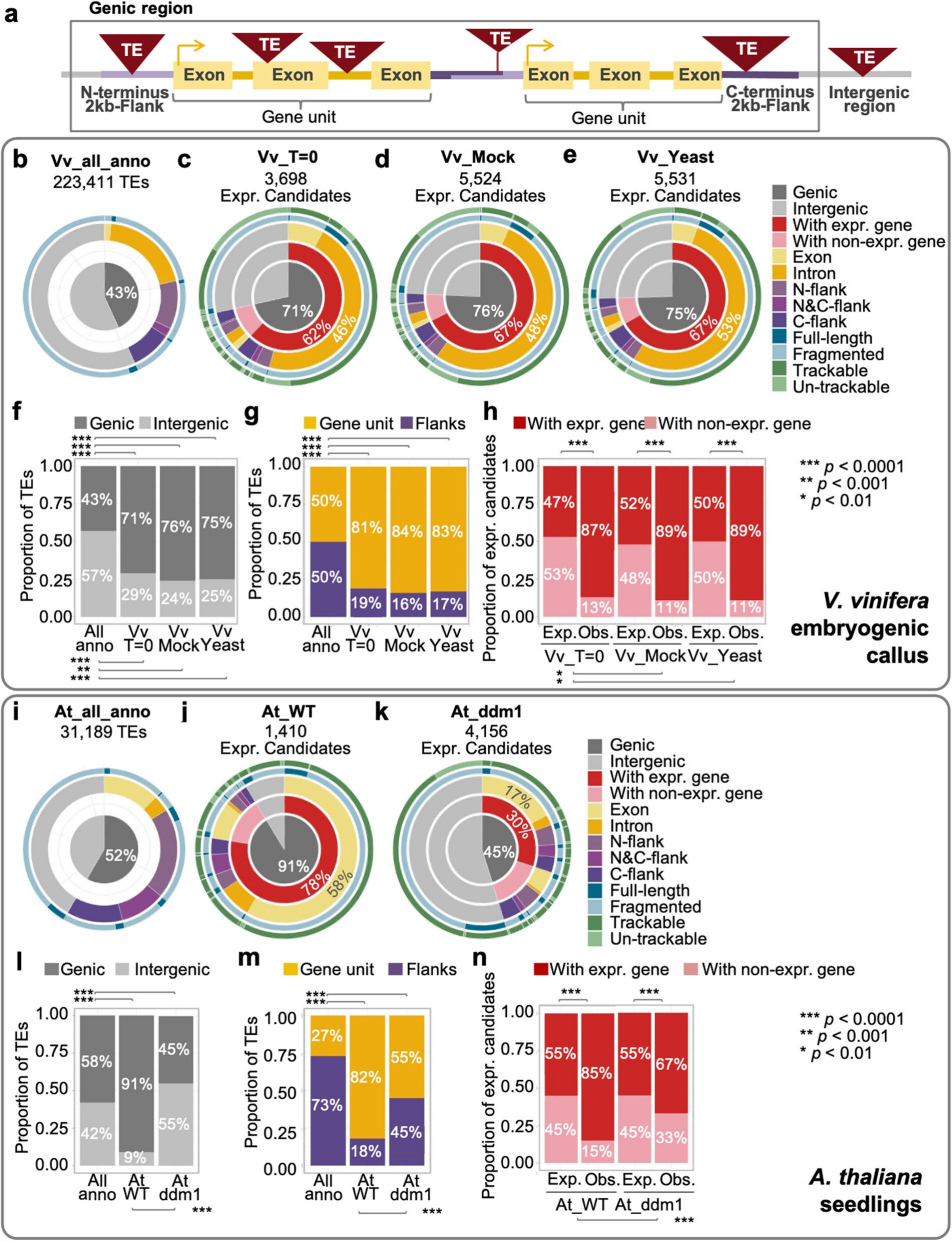
Location bias of expressed TE loci. **a** Illustration of TE insertions in the different genomic region (genic or intergenic) or location (exon, intron, or flanks) relative to genes. **b-e** Hierarchical classification of all annotated *V. vinifera* TE loci (**b**) and all TE expression candidates of Vv_T = 0 (**c**), Vv_Mock (**d**), and Vv_Yeast (**e**), by location, integrity, and distinctiveness. TE loci were categorised in the order of region (centre), the transcriptional activity of co-localised genes (2nd layer), location (3rd layer), integrity (4th layer), and the presence/absence of unique-mapping reads (outer-most layer). **f-h** Hundred percent stacked bar charts showing the proportion of all annotated TE loci of *V. vinifera* and TE expression candidates (Vv_T = 0, Vv_Mock, and Vv_Yeast) distributed in genic/intergenic regions (**f**), in gene unit (including exon and intron)/flanks (**g**), and with expressed/non-expressed genes (**h**). **i-k** Hierarchical classification of all annotated *A. thaliana* TE loci (**i**) and all TE expression candidates of At_WT (**j**) and At_ddm1 (**k**), by location, integrity, and distinctiveness. **l-n** Hundred percent stacked bar charts showing the proportion of all annotated *A. thaliana* TE loci and TE expression candidates (At_WT and At_ddm1) distributed in genic/intergenic regions (**l**), in gene unit/flanks (**m**), and with expressed/non-expressed genes (**n**). Colour codes for different categories of location, integrity, and distinctiveness are as indicated at the right panel. Chi-square tests with *p*-value < 0.01 were as indicated. Expr., expressed; Non-expr., non-expressed; N-flank, N-terminal 2 kb-flanking region of gene; C-flank, C-terminal 2 kb-flanking region of gene; Exp., expected proportion; Obs., observed proportion

A number of recent reports highlight that TE mobilisation and reintegration into host genomes often show distinct insertion bias [9, 26–30]. However, little is known regarding the genome-wide distribution of transcriptionally active TE loci. In order to investigate whether there is location bias between all annotated TEs and TEs representing expression candidates, we compartmented the annotated reference genome into genic and intergenic regions. The genic region comprised gene units, which were made of exons and introns that were bounded by the transcription start and stop sites of genes, and flanking regions of genes which encompassed 2 kb upstream (N-flanks) and 2 kb downstream (C-flanks) of corresponding transcription start and stop sites (Fig. 5a). All annotated TE loci with genomic coordinates that were found to intersect with specific gene compartments were categorised and hierarchically ordered with respect to being present in genic/intergenic regions, within the genic region (e.g. exon, intron, flanks), and whether the TE loci contained a fragmented or full-length element. In grapevine, over half of all annotated TE loci were found to be located in intergenic regions (126,976 TEs, 56.83%), while 96,435 (43.16%) TEs co-localised with genes (Fig. 5b). Approximately half of the genic TEs were found to be present in flanking regions, without particular preference for either the 5′-flank or 3′-flank. TEs that mapped to gene units were mostly found in the introns (Fig. 5b; Additional file 5: Table S4).

The expression candidates we defined in grapevine were then classified in the same way, with additional categories added, including the transcriptional activity of genes that were co-localised with TE candidates (i.e. TEs associated with expressed or non-expressed genes) and the presence or absence of unique-mapping reads (trackable or un-trackable TE candidates). Untreated embryogenic callus (Vv_T = 0), Vv_mock, and Vv_Yeast showed that 71.47, 75.69, and 74.62% of the expression candidates, respectively, were found within the genic regions (Fig. 5c-e, Additional file 5: Table S4). The goodness of fit X-square test showed that the increased proportion of expressed TE candidates was significant across all treatments (Fig. 5f). Delving deeper into the insertion context, about two-thirds of these genic TE expression candidates overlapped with introns with a significant bias toward introns of expressed genes (Fig. 5c-e, g, h; Additional file 5: Table S4).

In order to test whether our observations were isolated to grapevine or represent a trend across plants, we examined the location bias of expression candidates within the *A. thaliana* dataset. We found 58.37% of *A. thaliana* annotated TE loci were located in the genic region, while TEs in gene units and flanking regions comprised 16.00 and 42.37% of the total pool, respectively (Fig. 5i; Additional file 5: Table S4). Remarkably, the majority of the TEs annotated within the ‘gene unit’ overlapped with exons (Fig. 5i), contributing to 12.42% of all annotated loci. This high proportion of exonic TE loci is likely due to issues with the TE annotation established by Jin et al. [51], where considerable numbers of long TEs are found to overlap with multiple exons and introns, which were annotated based on the gene annotation file deposited in Ensembl Plants (see Methods), and therefore were preferentially categorised as exonic TEs.

In wild-type *Arabidopsis* (At_WT), TE expression candidates locating in genic regions accounted for 91.28% of the 1410 expression candidates, of which 1093 loci co-localised with expressed genes (Fig. 5j; Additional file 5: Table S4). In addition, the proportion of the gene-unit loci subset had changed significantly from 16% of all annotated TE loci (Fig. 5i) to 74.61% of the expression candidates in At_WT (Fig. 5j; Additional file 5: Table S4). The majority of these expression candidates in the gene-unit were associated with expressed genes (65.67% to the total expression candidates; Additional file 5: Table S4). In comparison, however, while intergenic TE expression candidates comprised only 8.72% of the expression candidate pool in At_WT, the proportion of TE-expression candidates in the At_*ddm1* mutant was significantly higher at 54.79% of expressed TE loci (Fig. 5k, m, Additional file 5: Table S4). In this mutant background the number of expression candidate loci in the gene-unit subset dropped from 74.61% in At_WT to only 24.95% of the expression candidates in At_*ddm1* mutant. These results were found to be statistically significant using the goodness of fit X-square test (Fig. 5l-n).

The observed preference for expressed TE loci to be located in genic regions may be explained by either a general increase in the proportion of genic located TE expression candidate from most TE families or simply a reflection of a genic-enriched annotation of a few TE families that largely contribute to the pool of expression candidates. To test these two assumptions, we further looked into the location distribution of all annotated TE loci and TE expression candidates of individual TE families. The genic and intergenic proportions of annotated TE loci and expression candidates were plotted for families belonging to Copia, Gypsy, LINE, hAT and MULE, the five super-families that contributed to the majority of expression candidates (Additional file 2: Fig. S7-S11). This analysis first looked at the genic and intergenic proportion of all annotated TE loci grouped by families. We then categorised TE expression candidates, in the same way, to examine whether the genic proportion of TE expression candidates was higher than that of annotated TE loci. Considering all annotated TE loci, we observed that for Copia families about two thirds are underrepresented (< 50%) in genic regions (Additional file 2: Fig. S7A). When considering Copia TE expression candidates in Vv_T = 0, 58 of the 71 families with expression candidates demonstrated a higher proportions of expression candidates in genic regions (Additional file 2: Fig. S7B) compared to annotated TE loci (Additional file 2: Fig. S7A), supporting that Copia TE expression candidates tend to be located in the genic regions in the Vv_T = 0 samples. This trend for Copia families was also observed in Vv_Mock and Vv_Yeast (Additional file 2: Fig. S7C-D). Analysis of Gypsy, LINE, hAT and MULE TE families and expression candidates revealed similar trends as observed for Copia (Additional file 2: Fig. S8–11).. The same analysis for the location preference within the genic region subset (exon, intron, and flanking regions) also reveals that the tendency for colocation of TE expression candidates in the intronic fraction of expressed genes is widely presented in most TE families of *V. vinifera* (Additional file 2: Fig. S12-S16).

The distribution bias of TE expression candidates suggested a degree of tolerance for the transcription of TE loci within, or proximal to, expressed genes. We were interested to see if the observed distribution was biased toward non-autonomous TE loci, which are assumed to pose less of a mutational load to host cells than the structurally intact TE loci. We therefore investigated the location distribution of the structurally intact TE loci of Copia-3 and Copia-23, the two families estimated to have mobilised most recently among all LTR-TE families in *V. vinifera*. Among the total annotated structurally intact TE loci of Copia-3 and Copia-23, 55 of 87 (63%) and 117 of 177 (66%) loci were found within introns (Additional file 2: Fig. S17A), respectively. Seventy-four and 138 of these structurally intact Copia-3 and Copia-23 TE loci were identified as TE expression candidates in at least one experimental condition. Astonishingly, 59 (80%) of the 74 structurally intact Copia-3 expression candidate loci and 104 (75%) of the 138 structurally intact Copia-23 expression candidates were found to be co-localised with expressed genes, primarily within introns of these genes (Additional file 2: Fig. S17B). Taken together, this clearly suggests a strong distribution bias of structurally intact and therefore potentially autonomous TE expression candidates to within expressed genes in LTR-TE families that are most recently active in this genome.

### The transcriptional dynamics of TEs is closely related to that of co-localised genes

The over-representation of TE expression candidates in proximity to expressed genes in the *V. vinifera* and wild-type *A. thaliana* indicates a tolerance for the transcriptional activity of intragenic TEs within these genes. This suggested to us that these TEs behave in a ‘hitchhiker-like’ manner where TEs co-located with expressed genes are able to take advantage of the permissive epigenetic landscape associated with the expressed genes for their own expression [13, 52].

**Fig. 6:**
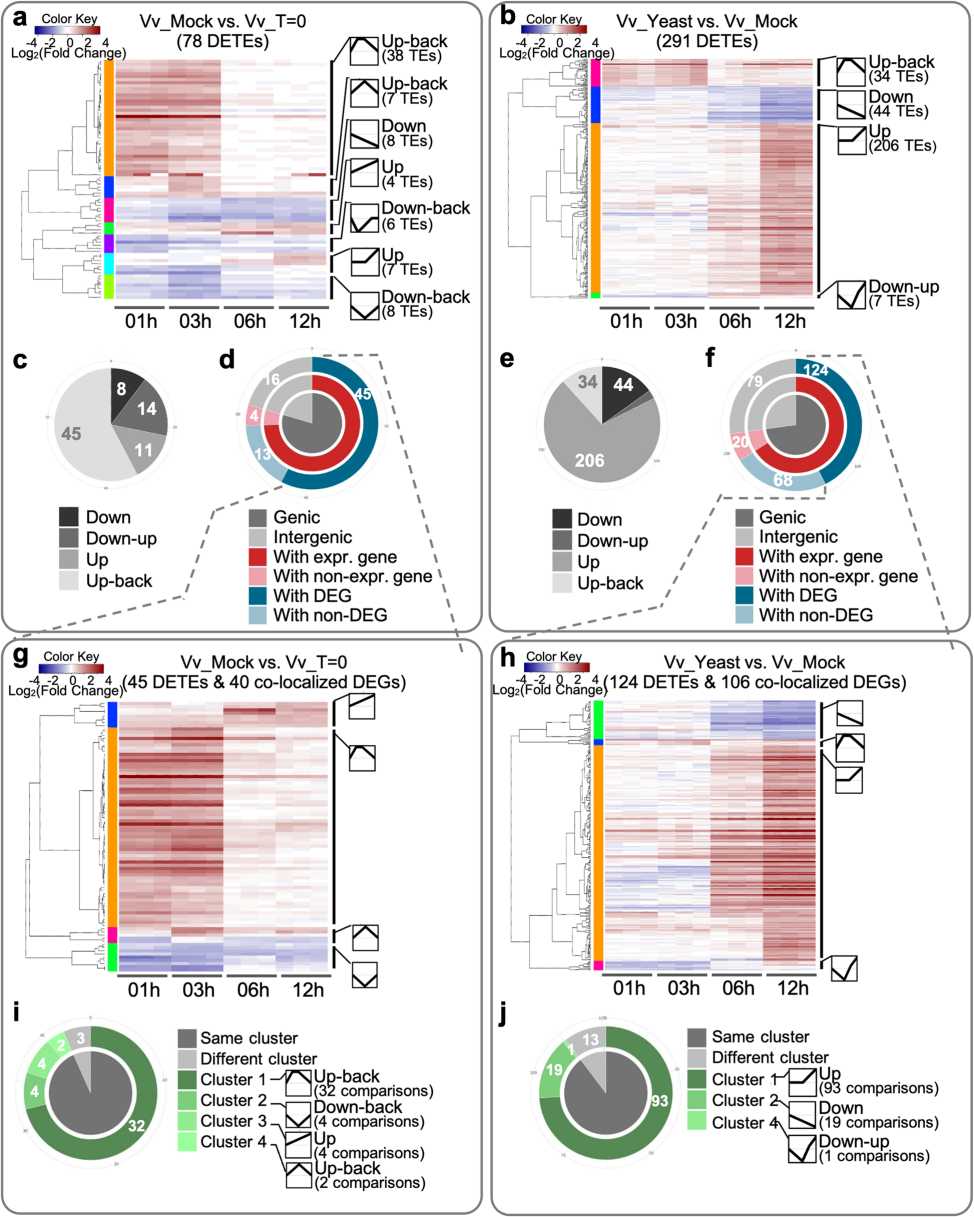
Expression dynamics of differentially-expressed TEs (DETEs) correlate with that of the co-localised differentially-expressed genes (DEGs). **a, b** Heatmaps showing hierarchical clustering of DETEs in Vv_Mock vs Vv_T = 0 (**a**), and DETEs in Vv_Yeast vs Vv_Mock (**b**), based on logarithmic-transformed fold-changes. The three replicates of each time point are individually presented. The time-series expression pattern of each cluster is depicted as line graph with the number of TE loci within each cluster indicated. **c, e** Pie charts to summarise the expression pattern of DETEs in Vv_Mock vs Vv_T = 0 (**c**) and DETEs in Vv_Yeast vs Vv_Mock (**e**). **d, f** Hierarchical classification of DETEs in Vv_Mock vs Vv_T = 0 (**d**) and DETEs in Vv_Yeast vs Vv_Mock (**f**) by the presence/absence of co-localised genes (genic/intergenic), the transcriptional activity of co-localised genes (with expr. Gene/with non-expr. gene), and the differential test of expressed genes (with DEG/with non-DEG). **g, h** Heatmaps showing hierarchical clustering of co-localised DETEs and DEGs in Vv_Mock vs Vv_T = 0 (**g**), and DETEs in Vv_Yeast vs Vv_Mock (**h**), based on logarithmic-transformed fold-changes. **i, j** Donut graphs to summarise the hierarchical clustering of co-localised DETEs and DEGs in Vv_Mock vs Vv_T = 0 (**i**) and DETEs in Vv_Yeast vs Vv_Mock (**j**) by expression pattern

As this ‘hitchhiker-like’ behaviour appears to be widely spread across plant lineages, we hypothesised that the co-localisation of TE and gene loci is likely due to genes that are stress-regulated and that co-located TEs would share a similar expression pattern to their ‘host-gene’. To test this, we first looked at the differential expression of genes in mock and yeast-treated samples and overlaid the presence and location of intron located and expressed TEs. While only 6 and 7% of DEGs contained intronic expressed TEs in Vv_Mock and Vv_Yeast, respectively, 14 and 13% of the non-DEGs co-located with expressed intronic TEs in Vv_Mock and Vv_Yeast (Additional File 2: Fig. S19). This result suggests that intron location of expressed TE loci is not restricted to stress-responsive genes. To address the possibility of the a shared expression pattern between co-located TEs and genes, differential transcriptional changes of TE expression candidates in the grapevine system were analysed using DESeq2 [53]. Due to the repetitive characteristics of TEs and that DESeq2 can only work with uniquely mapped read data, only the subset of expression candidates containing sequence divergence (i.e. trackable expression candidates) were suitable for this analysis. Differential expression analysis was performed on 5869 trackable expression candidates found in at least one of the three experimental conditions of the *V. vinifera* system (Vv_T = 0, Vv_mock, and Vv_Yeast). Hierarchical clustering of differentially expressed TEs (DETEs) revealed various expression patterns across the time course in response to different treatments (Fig. 6a, b). The mock treatment (Vv_Mock) showed that over 50% of the DETEs were transcriptionally activated in the first 3 h (3 h) of post-treatment and then returned to an expression level similar to that observed in Vv_T = 0 (Fig. 6a, c), illustrating an ‘up-back’ expression pattern. Interestingly, the majority (206 of 291) DETEs (70.79%) in the *H. uvarum* exposure treatment (Vv_Yeast) responded in an up-regulated manner (Fig. 6b, e).

Among the DEGs of Vv_Mock samples, 40 DEGs were co-localised with 45 of the 78 DETEs (Fig. 6d). In Vv_Yeast samples, 106 DEGs were co-localised with 124 of the 291 DETEs in this treatment (Fig. 6f). In an attempt to investigate the relationship of expression pattern between DEGs and the co-localised DETEs, these corresponding DETEs and DEGs were used for hierarchical clustering, in which DETEs and DEGs of similar expression pattern were grouped into the same clusters (Fig. 6g, h). Note that a small number of DETEs, especially DETEs within 2 kb flanking regions of genes, might co-localise with multiple DEGs and vice versa. Instead of arbitrarily excluding DETEs or DEGs that fell into this scenario, the comparison of the expression pattern of co-localised DETEs and DEGs was conducted on each DETE-DEG pair. The expression pattern of the 45 DETEs co-localized with 40 DEGs in Vv_Mock was then compared with that of paired DEGs, resulting in 45 pairs of DETE-DEG comparisons summarised in Fig. 6i, where 42 (93.33%) pairs of co-localised DETE-DEG showed concordant clustering between DETEs and corresponding DEGs. The same approach was applied on co-localised DETEs and DEGs of Vv_Yeast, in which 113 (89.68%) of the total 126 co-localised DETE-DEG pairs showed the same expression pattern between paired DETEs and DEGs (Fig. 6j). These findings indicate that the dynamic expression pattern of DETEs co-localized with DEGs tended to resemble that of the paired DEGs.

### Non-de novo TE transcription of intronic TE loci is associated with intron retention and exposure of premature termination codons

Aberrant alternative splicing, such as exon skipping and intron retention, has been observed at gene loci containing epigenetically unmasked intronic TEs [31, 54]. While TE expression candidates in the *V. vinifera* and *A. thaliana* systems show a strong location bias towards expressed genes, how broad the transcriptionally active intronic TE loci associated with aberrant alternative splicing remains unanswered. To interrogate this issue on a genome-wide scale and investigate the transcription of ‘competent’ TE-derived transcripts as well as potential fusion products of TEs and co-localised genes, we took advantage of long-read sequencing to determine transcript sequence integrity. Oxford Nanopore (ONT) cDNA sequencing was utilised for *V. vinifera* (P. noir UCD5 clone) embryogenic callus subjected to 12 h of continuous incubation with live *H. uvarum* culture (hereinafter Vv_Yeast12h) and the corresponding mock treatment (hereinafter Vv_Mock12h). This stress treatment experiment was independent of experiments used to derive RNA for the short-read RNA-seq analyses, We chose only the 12 h time point as we previously showed that with yeast treatment of embryogenic cell cultures, the majority of TE families exhibit a continual increase in transcript accumulation (Fig. 6b), and we thus hypothesised that this time point was likely to seed numbers of autonomous TE-RNAs.

The alignment depth of the ONT cDNA sequencing reads to the *V. vinifera* reference genome was comparable to that of the Illumina libraries (Additional file 6: Table S5). The quantification of gene and TE family expression level in the ONT dataset shows a medium to high level of correlation with that of the Illumina dataset (Spearman’s correlation coefficient *ρ* > 0.80 for genes; *ρ* > 0.58 for TE families; Additional file 2: Fig. S20).

**Fig. 7:**
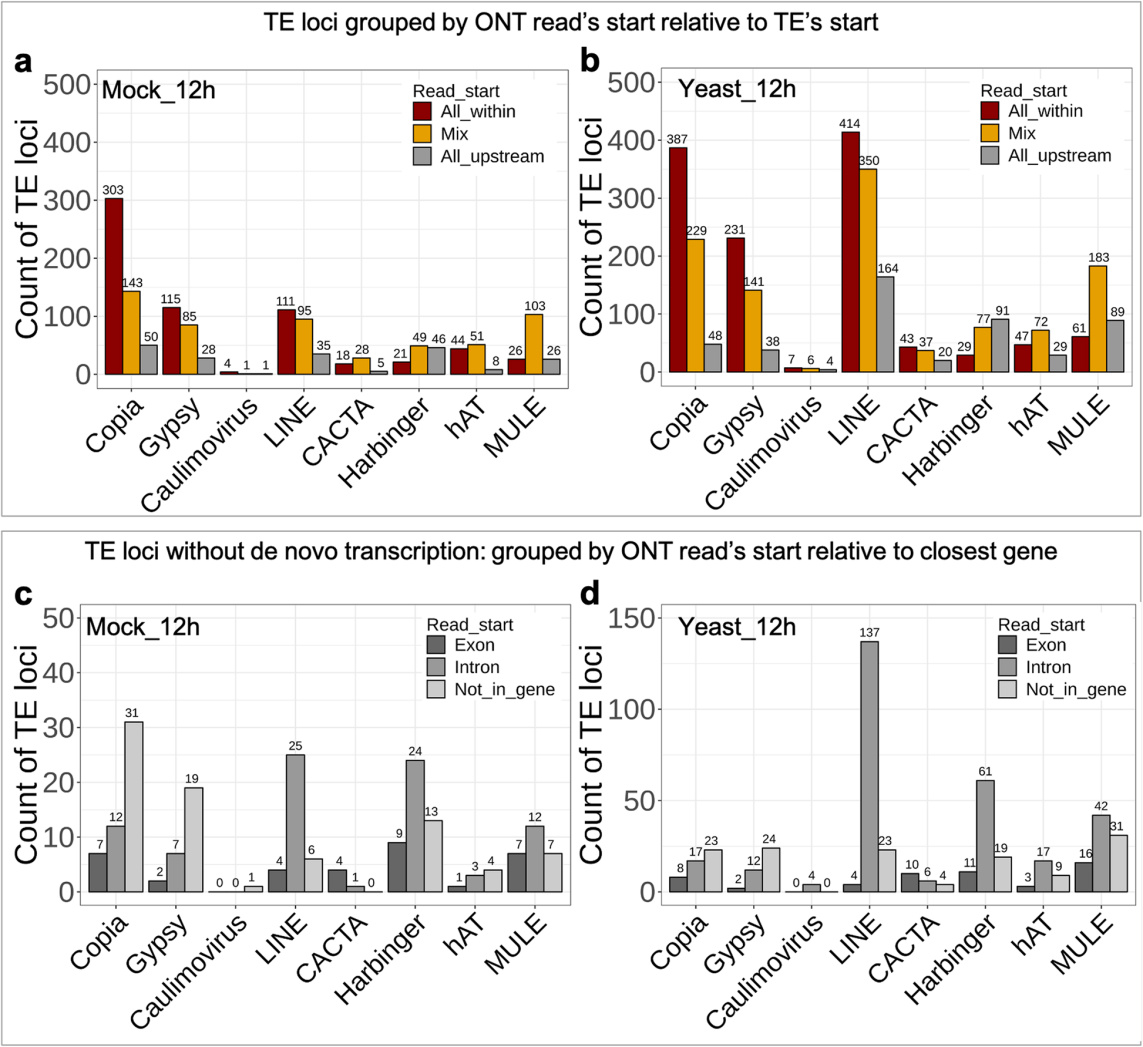
The frequency of non-de novo TE transcription differs by TE superfamily. **a, b** Expressed TE loci in (**a**) Vv_Mock_12h and (**b**) Vv_Yeast_12h were grouped by the mapped start site of ONT reads in relation to the annotated TE boundaries. **c, d** TE loci without de novo transcription in (**c**) Vv_Mock_12h and (**d**) Vv_Yeast_12h were categorised by the mapped start site of ONT reads in relation to the closest gene. “All_within”, all mapped ONT reads initiated within TE loci (i.e. de novo transcription); “All_upstream”, all mapped ONT reads initiated from the upstream of TE loci (i.e. non-de novo transcription); Mix, a mixture of mapped ONT reads representing de novo and non-de novo transcription

To understand the prevalence of TE transcription initiating from outside the annotated TE boundary (i.e. a non-de novo transcription), we examined the transcription start of ONT reads overlapping with TE loci. The results show that the frequency of non-de novo TE transcription varied by TE superfamily (Fig. 7). Most expressed Copia, Gypsy and LINE loci revealed transcription initiation from within the annotation boundary of these TEs. The type II DNA transposon loci investigated, exhibited a mixture of de novo and non-de novo transcription or transcription that was purely initiated outside of the annotation boundaries (Fig. 7a, b).

For TE loci without de novo transcription, the transcription start sites were further investigated in relation to the closest gene, and then these TE loci were grouped by whether the transcript initiated from exonic or intronic sites or did not initiate from an annotated gene (Fig. 7c, d). While the majority of non-de novo transcription of Copia and Gypsy was not initiated from annotated genes, the majority of LINE, Harbinger, hAT and MULE non-de novo transcripts were initiated from within introns (Fig. 7c, d). This result suggests that LINE and TIR-TEs are more likely to contribute to aberrant alternative splicing or gene-TE fusion transcripts.

The analysis of the ONT derived transcript data suggested a range of aberrant transcript splicing. To interrogate these data further we utilised the FLAIR pipeline [55] to categorise alternative splicing events into four categories: alternative 3′ splicing (Alt3), alternative 5′ splicing (Alt5), intron retention (IR) and exon skipping (ES). Gene-related alternative splicing features overlapping with TEs were also collected, following by estimation of the productivity (as per the definition in FLAIR pipeline, this denotes the ability of a transcript to produce protein), of gene transcripts having these alternative splicing features.

**Fig. 8:**
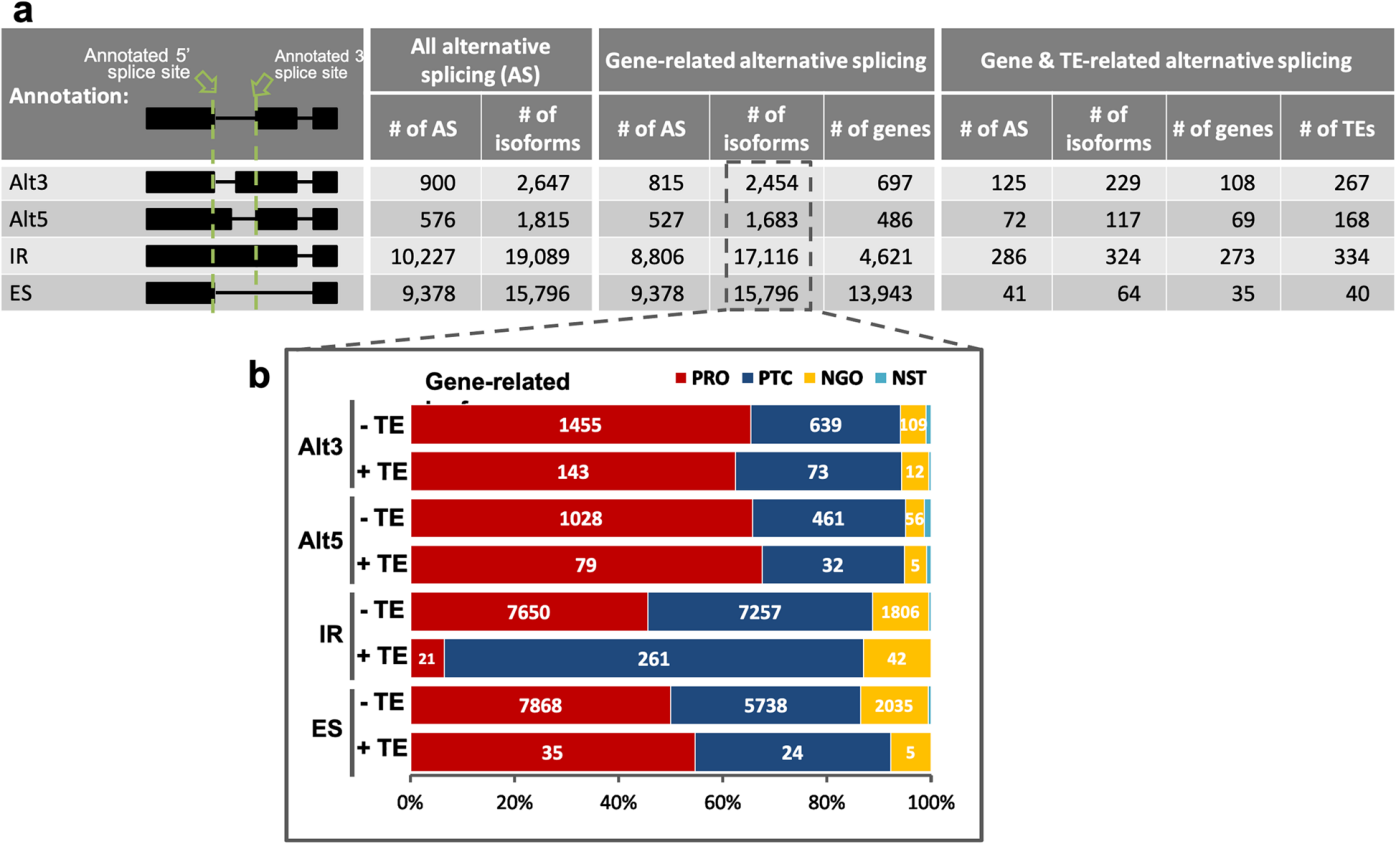
Intronic TE sequences retained in gene isoforms correlates with the presence of premature termination codon. **a** Illustration and summarisation of four types of alternative splicing. An alternative splicing feature could appear in multiple isoforms. Notably, there are more genes than the number of associated ES features, suggesting that, for some ES events, each may involve more than one gene. **b** A 100% bar chart to demonstrate the productivity of gene isoforms in the four types of alternative splicing, each further grouped as TE-present (+ TE) or TE-absent (− TE). AS, alternative splicing; Alt3, alternative 3′ splicing; Alt5, alternative 5′ splicing; IR, intron retention; ES, exon skipping; PRO, productive; PTC, premature termination codon; NGO, having no start codon; NST, having a start codon but no stop codon

Among the total 21,081 alternative splicing features identified by FLAIR, 19,526 (92.6%) of these are related to annotated genes. Of these features, over 90% were IR (8806 alternative splicing features) and ES (9378 alternative splicing features; Fig. 8a). Note that an isoform may contain multiple numbers and various types of alternative splicing features. Of the 19,526 alternative splicing features relating to annotated genes, only 524 (2.7%) of these overlapped with TEs (Fig. 8a). As expected, almost all TEs overlapping with Alt3, Alt5 and IR features are located within introns, while 22 of 40 ES-associated TEs overlapped with annotated exons.

The FLAIR pipeline was able to group splice variant transcripts into four types: productive (PRO), having premature termination codon (PTC, i.e. unproductive), no start codon (NGO), and having start codon but no stop codon (NST). To understand whether the presence of these TEs was associated with the productivity of the gene transcripts, the productivity of isoforms containing these gene-and-TE-related alternative splicing was estimated. The analysis showed that 50 to 68% of the isoforms having Alt3, Alt5, or ES remained productive, no matter whether the alternative splicing features overlapped with TEs (Fig. 8b). However, 80.6% of isoforms having TE-related IR were PTC, while the PTC proportion in isoforms having IR events non-overlapping with TEs was less than 45%. Looking into the estimated translation stop site of these isoforms containing TE-related IR feature, 196 of the 261 PTC isoforms exhibit premature stop codon exactly within the TE-overlapping IR feature (Additional file 7: Table S6). From the perspective of the isoform orientation, nine of the translational premature termination sites appear within TEs, two are after TEs, and the rest 186 isoforms show premature termination sites before the presence of TEs. The distance between TEs and the premature termination sites placed prior to TEs ranged from 2 bp to over 4 kb, with the first quartile, median and third quartile at 147 bp, 311 bp, and 693 bp, respectively (Additional file 7: Table S6).

Interestingly, different TE super-families were preferentially found among the four types of alternative splicing features. The retrotransposon LINE superfamily was over-represented in Alt3 and Alt5 alternative splicing events (Additional file 2: Fig. S21A, B), and Harbinger, a type II DNA transposon, was predominantly seen in IR features (Additional file 2: Fig. S21C). For ES features, MULE DNA transposon was the most predominant superfamily among all TE super-families (Additional file 2: Fig. S21D). Most of the TEs involved in alternative splicing were found to be fragmented. We observed only 10, 7, 34 and 1 full-length TE loci associated with Alt3, Alt5, IR, and ES features, respectively (Additional file 7: Table S6).

### Identification of potential loci contributing ‘full-length’ TE transcription allowing for autonomous mobilisation

Transcriptional activation of TEs in cell exposed to stress conditions has been widely studied in plants and primarily reports transcriptional activation at the family level [12, 56]. The proportion of these TE transcripts derived from structurally intact TE loci and thus capable of seeding TE mobilisation in the genome remains unclear. We have shown that within our *V. vinifera* system the detailed characterisation of transcriptional activation of TE loci under stressed conditions using short read RNA-seq data. The long-read capabilities of Nanopore sequencing has proved to be a useful tool in identification of TE transcripts engaged in the mobilisation of retro-elements [32]. We sought to use ONT RNA-seq to identify which, if any, of the annotated and transcribed TE loci identified earlier may possess full-length TE transcripts necessary for autonomous mobilisation. We initially set out to identify structurally intact TE loci with read alignments that met the criteria for transcription that would be required for autonomous mobilisation. These included intact LTR-TEs with > 90% breadth of coverage across INT domain that would suggest the production of transcripts capable of producing the *gag* and *pol* gene products required for mobilisation (Additional file 2: Fig. S22 A, B), structurally intact LINEs with > 0.9 breadth of coverage across whole elements (Additional file 2: Fig. S23 A, B), as well as intact TIR-transposon (TIR-TE) with > 90% of the transposase ORF covered by ONT reads (Additional file 2: Fig. S24 A, B). In these data we identified 20 and 19 LTR-TE loci in Vv_Mock12h and Vv_Yeast12h libraries, respectively (Additional file 2: Fig. S22 C). These included Copia-3, Copia-23, and Gypsy-V1. For LINE retrotransposons, only a single VLINE7 locus and two VLINE 8 loci were selected (Additional file 2: Fig. S23 C). For TIR-TE, three hAT-7 loci in Vv_Mock12h library revealed > 0.9 breadth of coverage across the transposase ORF (Additional file 2: Fig. S24 C).

We were particularly interested in identification of contiguous cDNA-seq reads that mapped across the appropriate length of identified loci. The ONT read should meet two criteria to prove that a full-length TE transcript was present. Firstly, depending on the expected de novo transcription characteristics of mapped TEs [57], the long-read should be at least as long as the INT domain for LTR-TEs [3], the transposase ORF for DNA transposons, or the full feature of the TE locus for LINE [57]. Secondly, this read should have its TE-mapped bases almost as much as its read length. Taking Copia-23 as an example, the ONT reads mapping to the 19 structurally autonomous Copia-23 loci were all shorter than 3000 bp (x-axis, Additional file 2: Fig. S25 A), whereas the size of the canonical Copia-23 INT domain is 4084 bp. Only a single read longer than 3 kb was identified but this exhibited a very poor mapping quality to the element (y-axis, Additional file 2: Fig. S25 A). In addition, as expected, given the conservation in sequence of these loci, the majority of these ONT reads were multi-mapping (red dots, Additional file 2: Fig. S25 A). This analysis highlighted an inconsistency between lengths of ONT reads and mapping of these reads to TEs. To determine the factors underlying this inconsistency, the alignment of the start and end sites of ONT reads mapped to Copia-23 loci were surveyed. As illustrated in Additional file 2: Fig. S25 B and C, the alignment of the 5′ and 3′ ends of reads could be grouped into three categories, internal, external, and clipped. Our analysis revealed that most of the ONT reads represented transcription initiated within the Copia-23 loci (Additional file 2: Fig. S25 B). However, the tail of the reads were mostly clipped due to the sequence discrepancy between ONT reads and TE loci (Additional file 2: Fig. S25 C). Only a few reads extended through the annotated boundary of TE loci. Overall, there is no convincing evidence of transcription from potentially autonomous Copia-23 loci annotated in this genome.

**Fig. 9:**
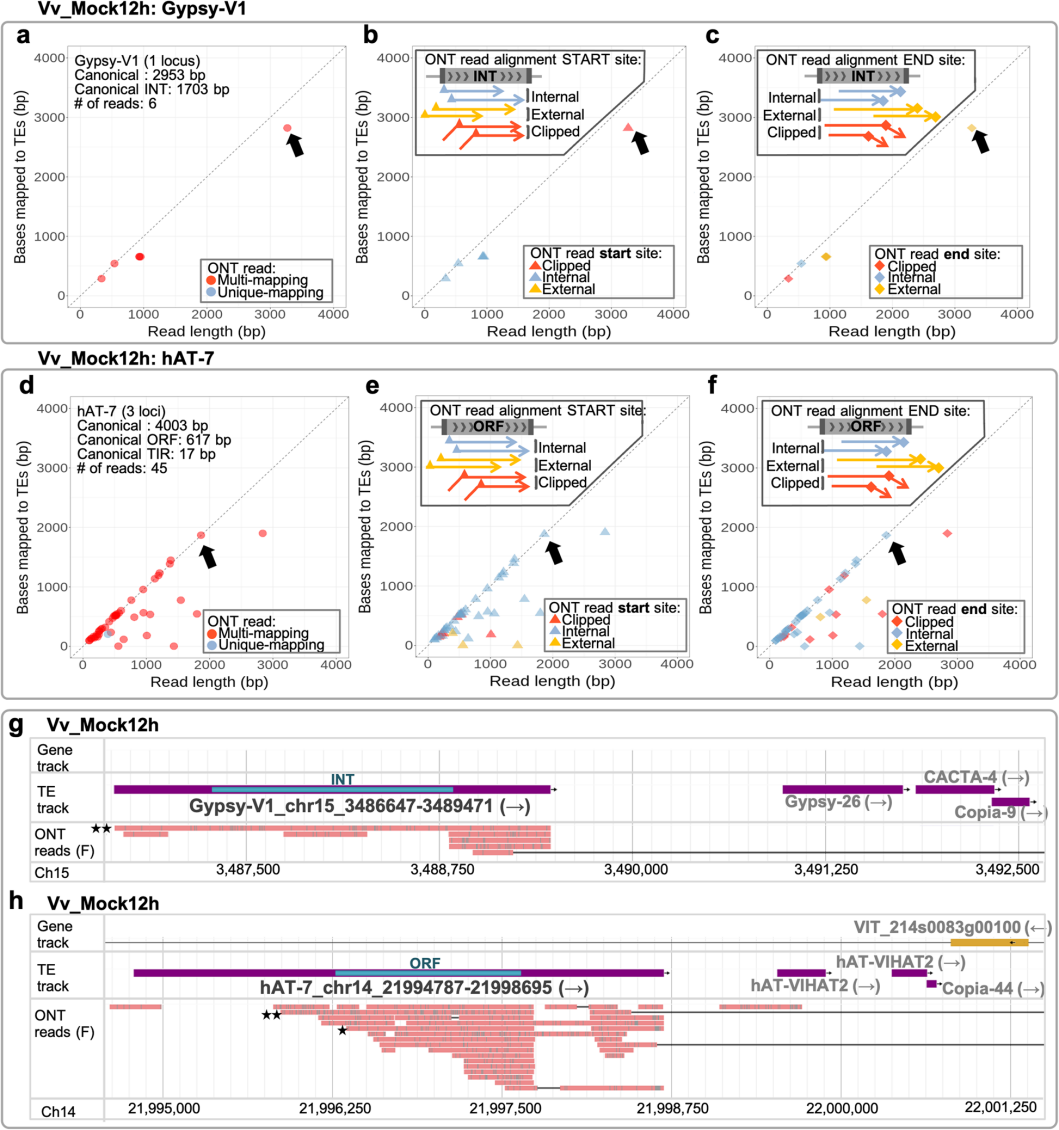
Identification of structurally intact TE loci potentially having transcripts for autonomous mobilisation. **a-c** The alignment properties of the ONT reads mapping to a structurally intact Gypsy-V1 locus whose INT domain was > 90% covered by ONT reads. For each read, the read length was plotted against the number of bases overlapping with the autonomous locus and coloured by mapping specificity (multi−/unique-mapping; **a**) and alignment start site (**b**) and end site (**c**) relative to the TE locus. All reads plotted in (**a**) were presented at the same coordinates in (**b**) and (**c**). The black arrows indicate an ONT read covering most of the structurally intact TE locus. Note that this ONT read is multi-mapping to other structurally intact Gypsy-V1 loci, but only the locus indicated here is TE expression candidate (i.e. supported by short-read data). **d-f** The alignment properties of the ONT reads mapping to three structurally intact hAT-7 loci whose ORF was > 90% covered by ONT reads. (**d**)-(**f**) were plot following the approach used for (**a**)-(**c**). **g-h** Genome browser images of the structurally intact Gypsy-V1 locus (Gypsy-V1_chr15_3486647–3,489,471) and the representative hAT-7 locus (hAT-7_chr14_21994787–21,998,695), whose INT and ORF (teal blue strips) was fully covered by individual ONT reads, respectively. The two black stars label full coverage of the INT or ORF, while the single black star marks > 90% coverage of the ORF. INT, internal domain; ORF, open reading frame; F, forward reads

The situation described for the Copia-23 loci was also observed in the TE loci of Copia-3, Gypsy-V1, VLINE7, VLINE8, and hAT-7 (Fig. 9; Additional file 2: Fig. S25). Only a single ONT read mapping to an autonomous Gypsy-V1 locus and eight reads of hAT-7 appeared to adequately cover the bases of the INT (Fig. 9 a-c) or ORF (Fig. 9 d-f) of the respective TE loci. The genome browser image of the only Gypsy-V1 locus demonstrates the full coverage of this locus by a single ONT read (Fig. 9 g). The genome browser image for hAT-7 shows ONT reads covering the ORF of the hAT-7 in chromosome 14 (Fig. 9 h). These data suggest the observed transcription of Gypsy-V1 and hAT-7 may allow limited mobilisation of these elements.

## Discussion

Here we have shown an efficient strategy to capture individual loci of TE expression candidates in the *V. vinifera* and *A. thaliana* systems and detailed the characteristic profile of these loci.

We demonstrate that, when the epigenetic system is not compromised, TE expression candidates comprised less than 3 and 5% of all annotated TEs in the *V. vinifera* and *A. thaliana* systems, respectively. When the epigenetic regulatory system is compromised, such as in the *ddm1* mutant of Arabidopsis, however, we found that the number of TE expression candidates was significantly increased, highlighting the role of *DDM1* in epigenetic silencing of transposons [18]. The high proportion of fragmented and trackable expression candidates clearly shows that much TE transcription is derived from relics of autonomous elements. It has been reported that short and fragmented TE loci (< 2 kb) are often lacking CHH methylation, indicating they may be less efficiently targeted by RNA dependant DNA methylation (RdDM) derived silencing [57]. In contrast, large and structurally intact TE loci are enriched with CHH methylation and tend to be efficiently targeted by RdDM [57]. This lessening of TE-focused epigenetic silencing may reflect the accumulation of sequence polymorphisms over evolutionary time, leading to a divergence and degradation of TE sequences at individual loci that eventually reaches a point where the endogenous siRNAs system fails to recognise these elements [58], and thus results in permissive transcription of these TE loci. The repetitive nature of these loci is the basis for difficulties in discerning the origins of TE transcription. Additionally, these mechanisms might also explain the over-representation of fragmented and trackable TE loci in the expression candidate pool. However, to address this fully, the patterns of accumulation of DNA methylation at these loci and identity of various siRNA species targeting individual TE loci need to be determined.

The transposition history of TE families likely contributes to varying proportions of fragmentation and increased nucleotide variation among element loci. For example, TE families experiencing the most recent mobilisation bursts tend to contribute structurally intact and un-trackable TE loci in transcript pools. Using the Copia-3 and Copia-23 families from grapevine as an example, the relatively high proportion of full-length, un-trackable loci is a reflection of recent mobilisation compared to older families. As a result, a sequencing read derived from the TE locus of a recently mobilised lineage of a TE family cannot be attributed to a single locus; rather, it is mapped to most of the TE loci of this lineage, and thus numbers of TE loci of the transcribed lineage are identified as expression candidates (Additional file 2: Fig. S3-S4). This finding might also reflect the notion that young TE families or individual loci that mobilised recently are more likely to be transcriptionally active than the old elements upon specific environmental cues [48].

The findings discussed above only relate to those elements within the reference genome and that have been annotated. There is a high likelihood that there are number of elements that have inserted into novel locations the genome of the Pinot Noir plant was used as source material for these experiments. Much recent discussion and experimentation supports a view that a single reference genome sequence cannot capture the important structural variation that may underpin phenotypic variation within a population [59, 60]. While our data may give hints at new loci a whole genome long read sequencing of the parent plant, alongside an in depth transposon assembly would be required to validate any putative insertions that differ from the reference. Alternative approaches using long-read genome sequence to identify structural variation and mapping approaches such as SNIFFLES [61] appear to provide another pathway forward to explore novel structural variation with existing reference resources. It should be noted, however, that while these approaches will capture structural variation, primarily insertions or deletions, caused by both type I and type II TEs further analysis would be required to identify and validate the TEs involved. In Arabidopsis, the heat-sensitive LTR retrotransposon, *ONSEN*, is transcriptionally activated in wild-type plants subjected to heat-shock treatment. However, this activation has only been observed in *nrpd1–3* mutant backgrounds which have lost the function of an RNA polymerase required for siRNA production in the RdDM pathway [48, 62]. Structurally intact *ONSEN* loci having identical LTRs contribute more *ONSEN* transcripts in both Arabidopsis wild-type and *nrpd1–3* background, and more new insertions in the progeny of heat-treated *nrpd1–3*, than the *ONSEN* loci having non-identical pair of LTRs [48].

In grapevine a retrotransposon has been shown to contribute to berry colour. The insertion of a structurally intact Gret1, a Gypsy retrotransposon, at the promoter of VvMybA1 gene in Cabernet disrupted the gene expression and gave rise to the white grape variety Chardonnay [5, 63]. Indeed, all white grape varieties carry this mutation [64]. In the formation of the Ruby Okuyama, self-recombination between the Gret1-LTR at the VvMybA1 locus resulted in the removal of LTR and the INT domain, leaving a single LTR at VvMybA1 promoter, restoring gene expression and hence red berries [63]. The structure of the Gret1 locus at VvMybA1 suggests a relatively recent insertion [63]. While our data and the previous work of Lizamore [21] showed transcriptional activity of Gret1, no full-length Gret1 transcription was observed in our P. noir embryogenic callus (Fig. 3; Fig. 9; Additional File 2: Fig. S25), and it remains unclear which TEs may retain autonomous mobility in this black-skinned variety. While no evidence for Gret1 activity was observed we determined that Copia-3 and Copia-23 appeared as the most recently mobile LTR-TE families in P. noir (Fig. 4), both exhibiting lineages with low levels of sequence divergence that were transcriptionally active (Fig. 3; Additional File 2: Fig. S3-S4). Although these two TE families did not appear to be producing transcripts that would indicate autonomous mobilisation, their structurally intact loci may potentially contribute to new insertions under other treatment conditions that may include environmental stimuli and alterations in the effectiveness of epigenetic silencing of TEs.

It has been reported that LTR retrotransposons, in particular from the Copia superfamily, and DNA transposons, such as MITEs, exhibit insertion bias in favour of gene-rich or transcribed gene regions [9, 26–28, 62]. It has also been proposed that TE families, particularly those with low copy numbers (less than a few hundred per genomes), prefer integrating into genetically active locations in genomes, thereby gaining the opportunity for transcription and consequently mobilisation [52, 65]. However, while new insertions may favour gene rich euchromatic regions of the genome there is little evidence supporting that these locations also support the ability for co-localised TE loci to become transcriptionally active. Here, for the first time, we reveal the significant location bias of TE expression candidates towards expressed genes in all experimental conditions (Fig. 5; Additional File 2 Fig. S7-S16). Compromising epigenetic silencing of TEs, as seen in the *A. thaliana ddm1* mutant, leads to a greater array of transcribed TE loci outside of gene-rich regions. In the grapevine system, this location bias of transcriptionally activated TE loci particularly favours introns of expressed genes and is observed for most TE families becoming transcriptionally active. Furthermore, most of the structurally intact expression candidates of Copia-3 and Copia-23 families, the two youngest LTR-TE families in grapevine, were found within intron of expressed genes. We also found that this tendency towards expressed genic regions is not restricted to stress-responsive genes (Additional File 2 Fig. S19). Transcriptional co-activation of co-localised TE loci and genes is not only observed in our grapevine system (Fig. 6), but also in other plant species, including rice [66], maize [67, 68], and *A. thaliana* [69]. This coordinated transcription could benefit environmental adaptation and specific cell development [67, 68]. Together, our observations and the findings reported in these reports suggest a mobilisation cycle that positively reinforce genic insertions that predetermine the transcriptional and thus mobility of elements.

It remains unclear whether genic TE expression candidates confer the expression activity on co-localised genes through contributing the *cis*-regulatory element in TE sequences as previously reported [3–5, 8], or whether these genic TE expression candidates acquired transcriptional activity due to the requirement of permissive chromatin or epigenetic status granted to the co-localised and transcribed genes [65]. The former indicates a positive effect of the presence of TEs on gene activity, whereas the latter suggests a ‘hitchhiker-like’ behaviour of TEs and may not necessarily benefit gene activity [65]. In the ‘hitchhiker-like’ scenario, epigenetically unmasked TE loci can sometimes result in the production of fused TE-gene transcripts [70, 71] or aberrant transcript isoforms of the host genes [31]. Our data reveal the prevalence of non-TE initiated TE transcription, a small fraction of which was derived from either exons or introns (Fig. 7). We also show that gene transcript isoforms containing intronic TEs tend to possess premature termination codon and thus are estimated to be non-productive isoforms (Fig. 8b). These findings suggests that the intronic TE loci involved in intron retention may follow the ‘hitchhiker-like’ scenario and influence the transcription and translation productivity of the host genes. In addition, in the analysis of the alignment start sites of ONT cDNA read relative to structurally intact TE loci, 35 and 73 of the total 497 ONT reads analysed were found to cross the boundary of mapped TE loci (crossing-boundary) and possess soft-clipped tail, respectively (Fig. 9a-f; Additional file 2: Fig. S25). The former indicates TE transcription initiated from the upstream of the analysed TE loci, and the latter suggests a discrepancy between the reference and P. noir genomic sequences. Both (crossing-boundary and soft-clipped at the N terminals of the alignments) imply a certain amount of TE transcription initiating outside of the annotated element. Although transcriptional activation of TEs under stress conditions has been widely reported, our ONT cDNA sequencing data shows that the full-length transcripts derived from structurally intact TE loci is extremely low. This finding indicates that the so-called ‘TE activation’ commonly seen from short-read RNA-seq may not necessarily represent transcriptional activation leading to autonomous TE mobilisation and suggests that so called TE bursts occurring in wild-type populations in a natural environment are rare bursts of activity relative to evolutionary timescales [10]. A recent study in *A. thaliana,* pooled six ONT cDNA sequencing libraries, which included *A. thaliana* wild-type and four epigenetically compromised genotypes before applying an expression threshold at five ONT reads to annotate an active TE locus [32]; this generated more than 5 million reads for the model organism with a genome size of ~ 135 Mb. With a similar N50, we would require alignment depth at least 2.3 times deeper than the current Vv_Mock12h or Vv_Yeast12h libraries that we have interrogated. The *A. thaliana* genotypes used in Panda and Slotkin [32] include those which were compromised in epigenetic silencing that allows the constant de-repression of TE loci, whereas the grapevine embryogenic callus in our study was initiated from wild-type P. noir clone UCD5 and was subjected to stress treatment. In such a fully active epigenetic silencing environment, the likelihood of high levels of transcription of autonomous elements is low. That being said, providing a stress treatment that can lead to strong transcriptional activation of autonomous TE loci in the wild-type backgrounds, and with more sequencing and alignment depth we may be able to detect the full-length TE transcription. Alternatively, using genetic backgrounds compromised in epigenetic silencing and thus predisposed to TE activation, applying chemicals that inhibit proteins of the silencing machinery [72, 73], and/or conducting stress treatment according to the stress-responsive specificity of a particular TE family/subfamily [25, 62] may raise the levels of autonomous TE transcription.

## Conclusions

The expression landscape and properties of transcriptionally active TE loci have been enigmatic mainly due to the repetitive and self-proliferating characteristics of TEs. Here, using short-read and long-read sequencing technology, as well as combined analysis of unique-mapping and multi-mapping sequencing reads, we show the evidence that transcriptionally active TE loci derive from a surprisingly small portion of total annotated TEs in *V. vinifera* and *A. thaliana* when the epigenetic silencing system is not compromised. Although most TE transcripts were derived from fragmented and trackable TE loci that are incapable of autonomous mobilisation, our results indicate a strong tendency for TE expression candidates to be found within introns of expressed genes. This distribution bias is commonly found for most of the expressed TE families, and for structurally intact TE expression candidates of grapevine Copia-3 and Copia-23, the two LTR-TE families experienced the most recent mobilisation burst. It was also discovered that the pairs of co-localised TEs and genes shared the same differential expression patterns in response to applied stressors. We further reveal a strong association of expressed intronic TE loci that form part of the gene transcripts (i.e. TE-related intron retention) and the presence of premature termination codon within the retained intron sequences of these gene transcript isoforms. These together suggest a close relationship between the transcriptional activity of TEs and genes. Finally, despite that the transcriptional activation of TEs has been observed in response to stress in *V. vinifera* system [21], the observation that little full-length transcripts can be derived from structurally intact TE loci. Such conditions imply low transposition efficiency and that the proper combination of stress treatments and measurements to induce a wide scale of epigenetic relaxation (e.g. chemical inhibition [72] of critical points in the epigenetic silencing pathways) are required to trigger efficient TE mobilisation in the wild-type genetic background.

## Methods

### Plant material and experimental conditions

Embryogenic callus cultures were established from *V. vinifera* cv Pinor noir clone UCD5 and maintained in the C_1_^P^ medium according to Lizamore (2013) [21]. Stress treatment was conducted essentially based on the methods established by Lizamore (2013) [21]. Before the inoculation with the biotic stressor (live *H. uvarum* cultures), the embryogenic callus cultures were subjected to vigorous shaking in the hormone-free C_1_^P^ liquid medium (HF- C_1_^P^) to break the lumps of callus apart. Therefore, this procedure was considered the mock treatment (denoted as Vv_Mock), which resembles a wound treatment to the callus. Samples exposed to the biotic stressor were denoted as Vv_Yeast. For both mock and biotic stress treatments, samples were harvested at 1, 3, 6, and 12 h as shown in Fig. 1a. A common untreated 0 h time point (denoted as Vv_T = 0) for mock and the biotic treatment was taken prior to any treatment. All treatments and their associated time points consisted of three biological replicates. Detailed procedures of stress treatment can be found in Additional file 8: Supplementary methods.

### Short-read RNA-seq and data pre-processing

Total RNA of harvested embryogenic callus was isolated according to the manufacturer’s instructions of Spectrum Plant Total RNA Kit (Sigma). Each purified RNA sample was treated with DNase I following the TURBO DNA-free kit protocol (Ambion) to remove contaminating genomic DNA before being sent to New Zealand Genomics Ltd. (now Otago Genomics) for library preparation and 150-bp pair-end Illumina sequencing using a HiSeq 2500 sequencer.

Adapter sequences and low-quality bases were trimmed by fastq-mcf [74] before quality check using fastqc [75]. Trimmed quality reads were aligned to *V. vinifera* tRNA and rRNA sequences (Ensembl Plants database: https://plants.ensembl.org) using HISAT2 [42] with default setting and followed by the collection of unmapped reads using samtools [76]. The collected reads were aligned to the 12X PN40024 grapevine reference genome (Ensembl Plants database https://plants.ensembl.org) by HISAT2 with the parameters *–rna-strandness RF –dta –k 100*. In total, there were 27 samples for the short-read RNA sequencing, including the three biological replicates per time point (1, 3, 6, 12 h) per treatment (Vv_Mock and Vv_Yeast) and the three biological replicates of Vv_T = 0. The sequencing and alignment stats (Additional file 1 Table S1) show 41 to 45 million raw sequencing reads, of which 96.61% in average were retained after quality check and removal of adapters and tRNA/rRNA reads. The alignment rate ranged from 87.8 to 90.1% in Vv_T = 0 and Vv_Mock samples yet dropped to 78.8% in average in Vv_Yeast due to the contamination from the live yeast culture (Additional file 1 Table S1).

RNA-seq data of wild-type *A. thaliana* were obtained from Le et al. (2015) [40] (accession codes DRA002305 in DDBJ Sequence Read Archive at https://www.ddbj.nig.ac.jp/dra/), while the *ddm1* RNA-seq data was collected from Oberlin et al. (2017) [17] (accession code GSE93584 in NCBI Gene Expression Omnibus at http://www.ncbi.nlm.nih.gov/geo/). The pre-processing of these raw sequencing data follows the above-mentioned method used for *V. vinifera* dataset. *A. thaliana* tRNA/rRNA sequences were downloaded from Ensembl Plants (https://plants.ensembl.org). There were 118 to 172 million reads retained after quality check, adapter removal, and filtering tRNA/rRNA reads (Additional file 1 Table S1). Ninety-two percent to 99.5% of these reads aligned with the Arabidopsis reference genome TAIR10 obtained from Ensembl Plants (https://plants.ensembl.orghttps://plants.ensembl.org).

### Identification of TE expression candidates

This analysis is comprised of three sub-pipelines described as follows:

For the first sub-pipeline, sequencing reads unmapped to tRNA and rRNA sequences of the analysed species were aligned to the reference genome using HISAT2 [42] as mentioned in the previous section. The software *htseq-count* of the package Htseq [43] was then utilised (with the setting *–m intersection-nonempty*) to quantify unique-mapping reads aligning to annotated TE loci. TE loci having read count of more than ten were collected.

For the second sub-pipeline, reads mapping to the reference genome by the software HISAT2 (with the same setting for the first sub-pipeline) were further analysed using *bedtools coverage* and *bedtools intersect* [44] to calculate unique- and multi-mapping reads mapping to annotated TEs (a multi-mapping read would be counted multiple times) and the number of bases of a TE locus covered by reads (covered bases of a TE locus). These data were then utilised to calculate the average read depth of an individual TE’s mapped region. TE loci with more than ten reads and average read depth > 5 were collected in this sub-pipeline.

The third sub-pipeline was established based on the TEFingerprint’s workflow [46], which initially used BWA (*bwa mem*) [45] to align reads against the reference genome with default setting. TEFingerprint then processed the mapped reads to capture discordant unique-mapping reads (hereinafter dangler reads) whose paired mates were aligned to TE sequences. Individual reads mapping across the annotation boundary were excluded by enabling *–exclude-tails* in TEFingerprint [46]. TE loci flanked by more than ten dangler reads on either side and had been found to have more than ten reads mapped internally were collected.

Finally, TE loci collected by each sub-pipeline were all combined to form the pool of TE expression candidates.

For the analysis of *V. vinifera* system, the TE annotation was established by Lizamore et al. [36]. Grapevine’s gene annotation file (version 2.1) was obtained from the Grape Genome Database at CRIBI (http://genomics.cribi.unipd.it/grape/).

For *A. thaliana* analysis, the *A. thaliana* gene annotation file were downloaded from Ensembl Plants (https://plants.ensembl.org), whereas the corresponding TE annotation file was generated by Jin et al. (2015) [51] and is available from Prof. Molly Hammell’s lab web page (http://hammelllab.labsites.cshl.edu/).

The downstream analysis to profile TE expression candidates’ characteristics was conducted using R script. Details regarding the pipeline and the downstream analysis can be found in Additional file 8: Supplementary methods.

### Differential expression analysis

Grapevine gene expression was quantified by *htseq-count* of the package Htseq [43] with the setting *–m intersection-nonempty*. The raw read counts of expressed genes (FPKM> 1) were imported into DESeq2 [53] for differential expression analysis to identify genes differentially expressed by stress treatment over time. The raw counts were normalised by a DESeq2 function *varianceStabilisingTransformation* before calculating log2foldchange. Genes with adjusted *p*-value lower than 0.05 and log2foldchange > 1 (at least one of the four time points) were collected as DEGs.

For TE analysis, only TE loci with unique-mapping reads (i.e. trackable TE expression candidates) were included in the differential expression test. The raw counts generated by *htseq-count* were fed into DESeq2. The normalisation step of raw read counts was the same as for gene analysis. Trackable TE expression candidates with adjusted p-value lower than 0.05 and log2foldchange > 1 (at least one of the four time points) were collected as DETEs. The interrogation of the expression patterns across time was conducted with R script, which can be found at https://github.com/ting-hsuan-chen/TE_ExpressionCandidate.

### Long-read cDNA-seq and data analysis

To validate the presence or absence of full-length TE transcription with long-read cDNA sequencing, we performed the stress treatment experiment independent of that for short-read RNA-seq. We chose the timepoint of 12-h for the maximum biotic-stress exposure. The grapevine embryogenic callus was subjected to the mock treatment and live *H. uvarum* treatment following the method mentioned earlier. The callus of mock treatment was harvested after 12 h of recovering on the C_1_^P^ plate, whereas the yeast treatment involves 12 h of continuous incubation with the yeast before harvesting. The purpose of this experiment was to detect the full-length TE transcripts rather than quantification of gene or TE expression. Therefore we did not use biological replication in these treatments.

Total RNA was extracted and separated using NORGEN Plant microRNA purification kit (Norgen Biotek) according to the manufacturer’s instruction. This kit allows for the recovery and purification of long RNA as well as short RNA species. Genomic DNA contamination was removed with the standard protocol of the TURBO DNA-free kit (Thermo Fisher). The RNA quantity was measured by Qubit RNA BR (Broad-Range) Assay Kit (Thermo Fisher), and the quality was examined using Agilent 2100 Bioanalyzer, in which the resulting RIN value of each library was above 8.

The cDNA library was prepared following the protocol of the Oxford Nanopore cDNA-PCR kit (SQK-PCS109). Briefly, 50 ng of total RNA was reverse transcribed utilising a poly-A adapter primer, before the strand-switching step. The resulting full-length cDNA was further enriched by PCR, which involved 12–13 amplification cycles, each with 6 min of extension step. The amplified cDNA was purified by AMPure XP beads before the ligation of the 1D sequencing adapters. Finally, the cDNA library was loaded onto a R9.4.1 MinION flow cell and then sequenced using MinKNOW (version 18.12) control software for raw data collection only.

Base-calling was carried out offline using Guppy (version 3.2.1; https://nanoporetech.com/) before removing adapter sequences using Pychopper2 [77]. The full-length reads were mapped to the 12X PN40024 *V. vinifera* reference genome using minimap2 [78] with the pre-set option *-ax splice* for long-read splice alignment. For mapping self-proliferating and highly repetitive TE sequences, the output of up to 100 secondary alignments was allowed for individual TE analysis using the flag *-N 100 -ax splice* in minimap2. For analysis based on TE family level, the ONT reads were mapped to the set of 232 canonical TE sequences by running default minimap2 using *-ax splice* before using *bedtools coverage* to quantify mapped reads at the family level. For genes, based on grapevine gene annotation (version 2.1), FLAIR [55] pipeline was then applied to obtain high fidelity isoforms and quantify the isoform expression level as transcripts per million (TPM). The TPM of isoforms derived from the same gene was then summed up at the gene level for the overall quantification of gene expression. For individual TEs, ONT reads overlapping with TEs in sense orientation were collected and quantified by *bedtools intersect* and *bedtools coverage* [44] as previously described.

To compare the correlation between ONT and Illumina platforms at the individual gene level, genes’ TPM (logarithmically transformed) from ONT was plotted against FPKM (logarithmically transformed) from Illumina data, while the correlation was tested using Spearman’s correlation coefficient. The same approach was applied to TE families, in which the expression level of each TE family was obtained from TEtranscripts [51] for the Illumina dataset and from alignment with the canonical sequences followed by bedtools coverage analysis for ONT data.

Individual TE loci overlapping with at least one ONT read were collected to perform an intersection with the expression candidates obtained from Illumina libraries at a time point of 12 h. The intersected TE loci were supported by both sequencing platforms and thus considered as expressed TEs. Bedtools [44] and bash and R scripts were used to survey the alignment start sites of ONT reads in relation to annotated TE loci and the closest genes, as well as the identification of the potential origin of full-length transcription for autonomous TE loci. Details can be found in Additional file 8: Supplementary methods and https://github.com/ting-hsuan-chen/TE_ExpressionCandidate.

To identify expressed genes, an intersection between annotated genes with ONT TPM above one and genes with Illumina FPKM over one was performed. Genes having overlapping data from both platforms were considered transcriptionally active genes. The alternative-splicing analysis for expressed genes was conducted using FLAIR pipeline [55].

## Supplementary Information

**Additional file 1: Table S1.** Datasets and general mapping statistics of short-read RNA-seq data.

**Additional file 2: Figure S1.** The filtering thresholds of identification of TE expression candidates. **Figure S2.** Details of expression candidates identified by the pipeline across various experimental conditions. **Figure S3-S4.** The consensus neighbour-joining tree of full-length Copia-3 and Copia-23 elements. **Figure S5-S6.** Grouping reads mapped to Copia-3 and Copia-23 expression candidates. **Figure S7-S11.** Genic and intergenic distribution of TEs grouped by TE family (grapevine). **Figure S12-S16.** Gene-unit and flanks distribution of genic TEs grouped by TE family (grapevine). **Figure S17.** Location distribution of structurally intact loci of Coipa-3 and Copia-23. **Figure S18.** DEG expression pattern. **Figure S19.** Grapevine genes grouped by presence of intronic TEs. **Figure S20.** Correlation coefficient: ONT vs Illumina (gene and TE). **Figure S21.** Pie charts of TE families related to alternative splicing. **Figure S22.** Autonomous expression candidates: LTR-TEs. **Figure S23.** Autonomous expression candidates: LINEs. **Figure S24.** Autonomous expression candidates: TIR-TEs. **Figure S25.** Mock: Breadth of coverage of Copia-23.

**Additional file 3: Table S2.** Grapevine expression candidates grouped by family, distinctiveness and integrity.

**Additional file 4: Table S3.** Peak of insertion time of *V. vinifera* LTR-TE families.

**Additional file 5: Table S4.** location distribution of expression candidates.

**Additional file 6: Table S5.** ONT cDNA sequencing mapping statistics.

**Additional file 7: Table S6.** Statistics of alternative splicing containing TEs.

**Additional file 8.** Supplementary methods.

## Data Availability

All sequencing data generated in this study are available at the National Center for Biotechnology Information Gene Expression Omnibus (NCBI GEO, https://www.ncbi.nlm.nih.gov/geo/) under accession number GSE175475**.** Publicly available next-generation sequencing data were downloaded from NCBI GEO and DDBJ under the accession number specified in Le et al. (2015) [40] and Oberlin et al. (2017) [17], and are listed along with general mapping statistics in Additional file 1: Table S1. The computational scripts used for this research, including a pipeline to identify TE expression candidates, is available at https://github.com/ting-hsuan-chen/TE_ExpressionCandidate.

## Abbreviations

Alt3: Alternative 3′ splicing
Alt5: Alternative 3′ splicing
Bp: Base pair(s)
cDNA: Complementary DNA
C-flank: C-terminal 2 kb-flanking region of a gene
DDM1: Decrease in DNA methylation 1 (chromatin-remodelling protein)
DEG: Differentially expressed gene
DETE: Differentially expressed transposable element
DNA: Deoxyribose nucleic acid
epiRIL: Epigenetic recombinant inbred line
ES: Exon skipping
HAT: Histone acetylase
hATC: hAT C-terminal dimerization
IBM2: Increase in BONSAI methylation 2 (an RNA binding protein)
INT: Internal domain
IR: Intron retention
Kb: Kilobase pairs
LINE: Long interspersed repetitive element
LTR: Long-terminal repeat
LTR-TE: LTR transposable element
Mb: Megabase pairs
MITE: Miniature inverted-repeat transposable element
mRNA: Messenger RNA
MULE: Mutator-like element
N-flank: N-terminal 2 kb-flanking region of a gene
NGO: No start codon for mRNA translation
NST: Having start codon but no stop codon for mRNA translation
nt: Nucleotide
ONT: Oxford nanopore technology
ORF: Open reading frame
PRO: Productive in terms of mRNA translation into protein
PTC: Premature termination codon for mRNA translation
RdDM: RNA dependent DNA methylation
RNA: Ribose nucleic acid
RNA-seq: RNA sequencing
rRNA: Ribosomal RNA
siRNA: Small interference RNA
TE: Transposable element
TIR: Terminal inverted repeat
TIR-TE: TIR transposable element
tRNA: Transfer RNA
