# Supplementary material for: Comprehensive analysis of both long and short read transcriptomes of a clonal and a seed-propagated model species reveal the prerequisites for transcriptional activation of autonomous and non-autonomous transposons in plants
Source: Mob DNA. 2022 May 12;13:16. doi: 10.1186/s13100-022-00271-5 (PMC9097378; doi:10.1186/s13100-022-00271-5)
Supplement: Supplementary file 2 — Additional file 2: Figure S1. The filtering thresholds of identification of TE expression candidates. Figure S2. Details of expression candidates identified by the pipeline across various experimental conditions. Figure S3-S4. The consensus neighbour-joining tree of full-length Copia-3 and Copia-23 elements. Figure S5-S6. Grouping reads mapped to Copia-3 and Copia-23 expression candidates. Figure S7-S11. Genic and intergenic distribution of TEs grouped by TE family (grapevine). Figure S12-S16. Gene-unit and flanks distribution of genic TEs grouped by TE family (grapevine). Figure S17. Location distribution of structurally intact loci of Coipa-3 and Copia-23. Figure S18. DEG expression pattern. Figure S19. Grapevine genes grouped by presence of intronic TEs. Figure S20. Correlation coefficient: ONT vs Illumina (gene and TE). Figure S21. Pie charts of TE families related to alternative splicing. Figure S22. Autonomous expression candidates: LTR-TEs. Figure S23. Autonomous expression candidates: LINEs. Figure S24. Autonomous expression candidates: TIR-TEs. Figure S25. Mock: Breadth of coverage of Copia-23. [file 13100_2022_271_MOESM2_ESM.docx]

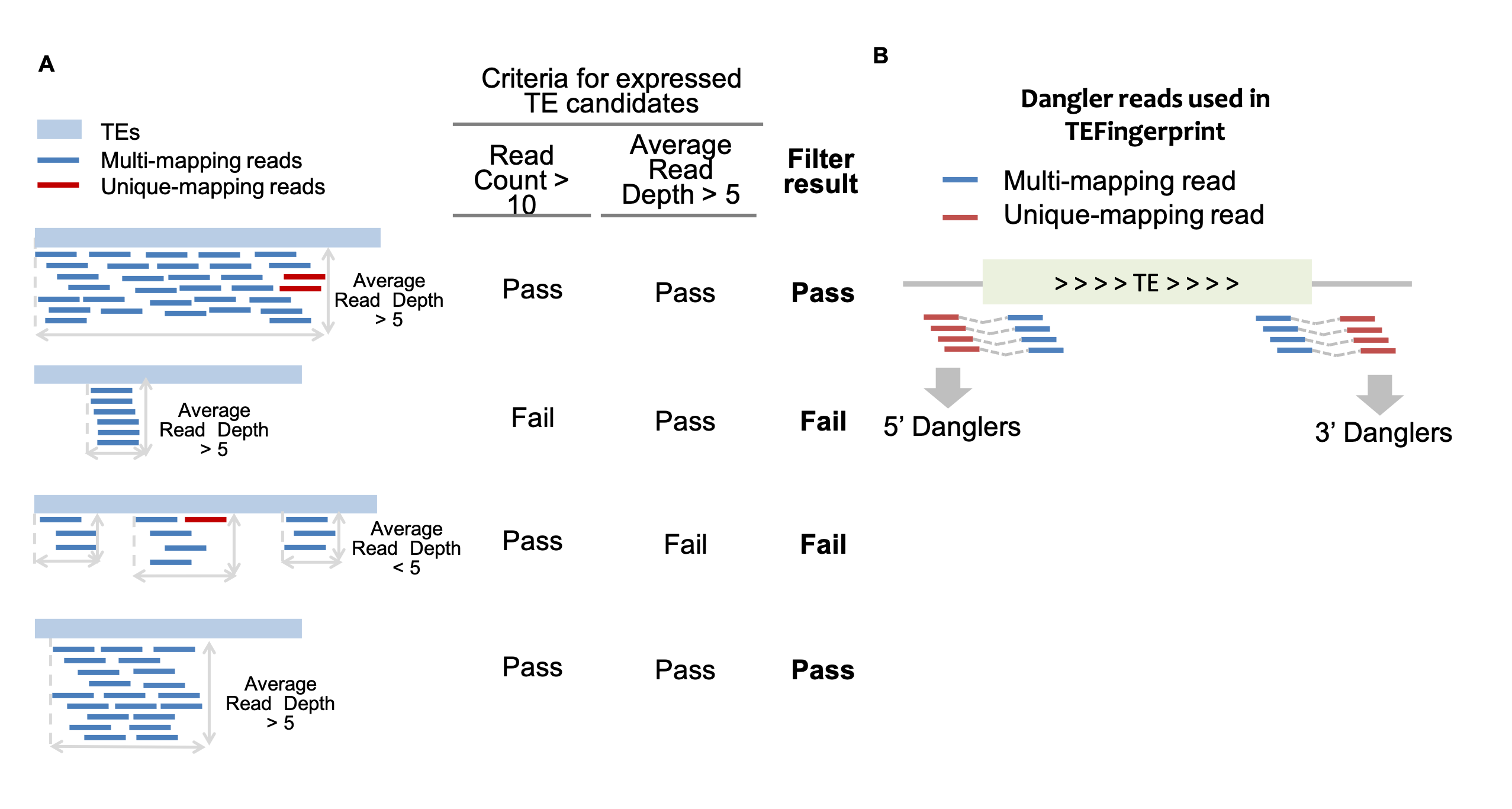


**Figure S1. The filtering thresholds of identification of TE expression candidates**

**(A)** Examples of filter step for the BEDtools-based sub-pipeline. To pass this step, a TE needs to show > 10 read count and >5 average read depth normalized by TE’s mapped region. **(B)** Illustration of the mapping strategy of TEFingerprint used in the pipeline. The blue fragments denotes reads mapping to TE sequences, whereas the red ones are the corresponding read mates (danglers) of TE-mapped reads, which are indicative of the transcriptional activity of a TE with transcription across boundaries.


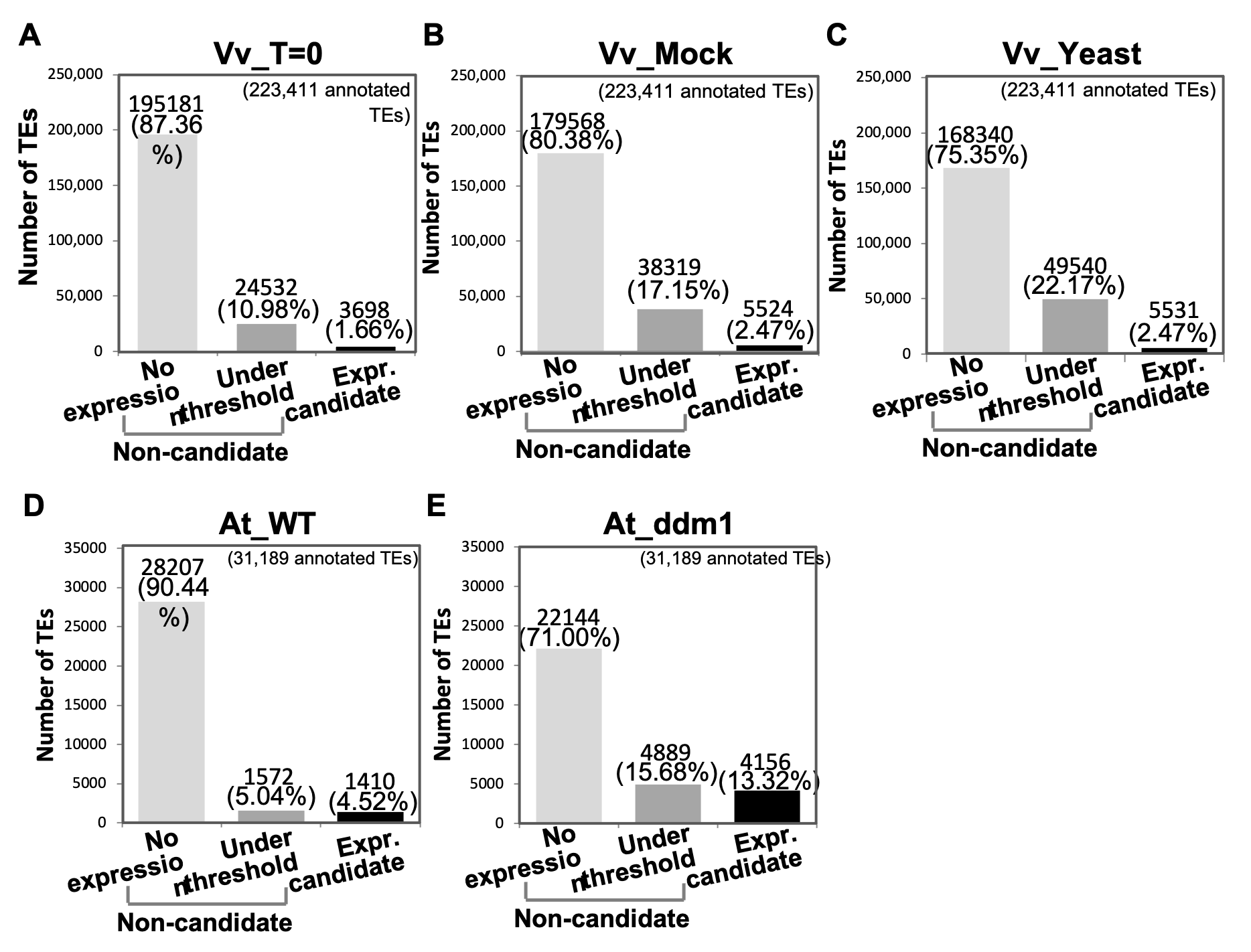


**Figure S2. Expression candidates identified by the pipeline across various experimental conditions**

All annotated TEs were categorized by transcriptional activity indicated at the x-axis and illustrated according to treatments/genotypes as shown in each graph: (A) Vv_T=0, (B) Vv_Mock, (C) Vv_yeast, (D) At_WT, and (E) At_ddm1.


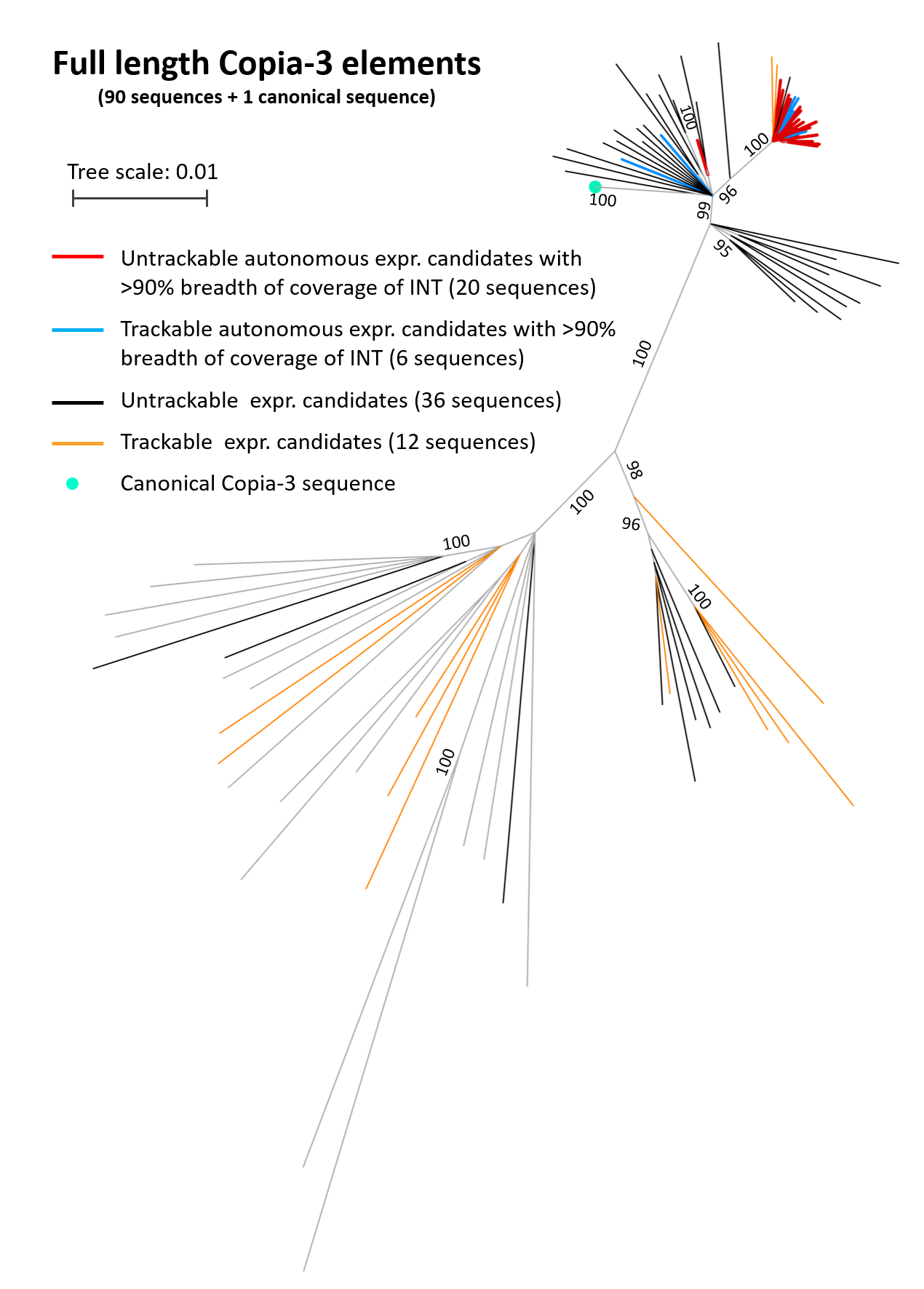


Figure S3. The consensus tree of full-length Copia-3 elements

Copia-3 elements retaining LTR pairs with >90% INT covered by sequencing reads were considered structurally autonomous expression candidates. Blue lines represent those having unique-mapping reads (trackable) blue, and the red lines denote the remaining untrackable ones. For the rest of the full-length Copia-3 expression candidates, those can be distinguished by unique-mapping reads were yellow, otherwise were coloured black. Grey lines denote branches or non-expression candidates.


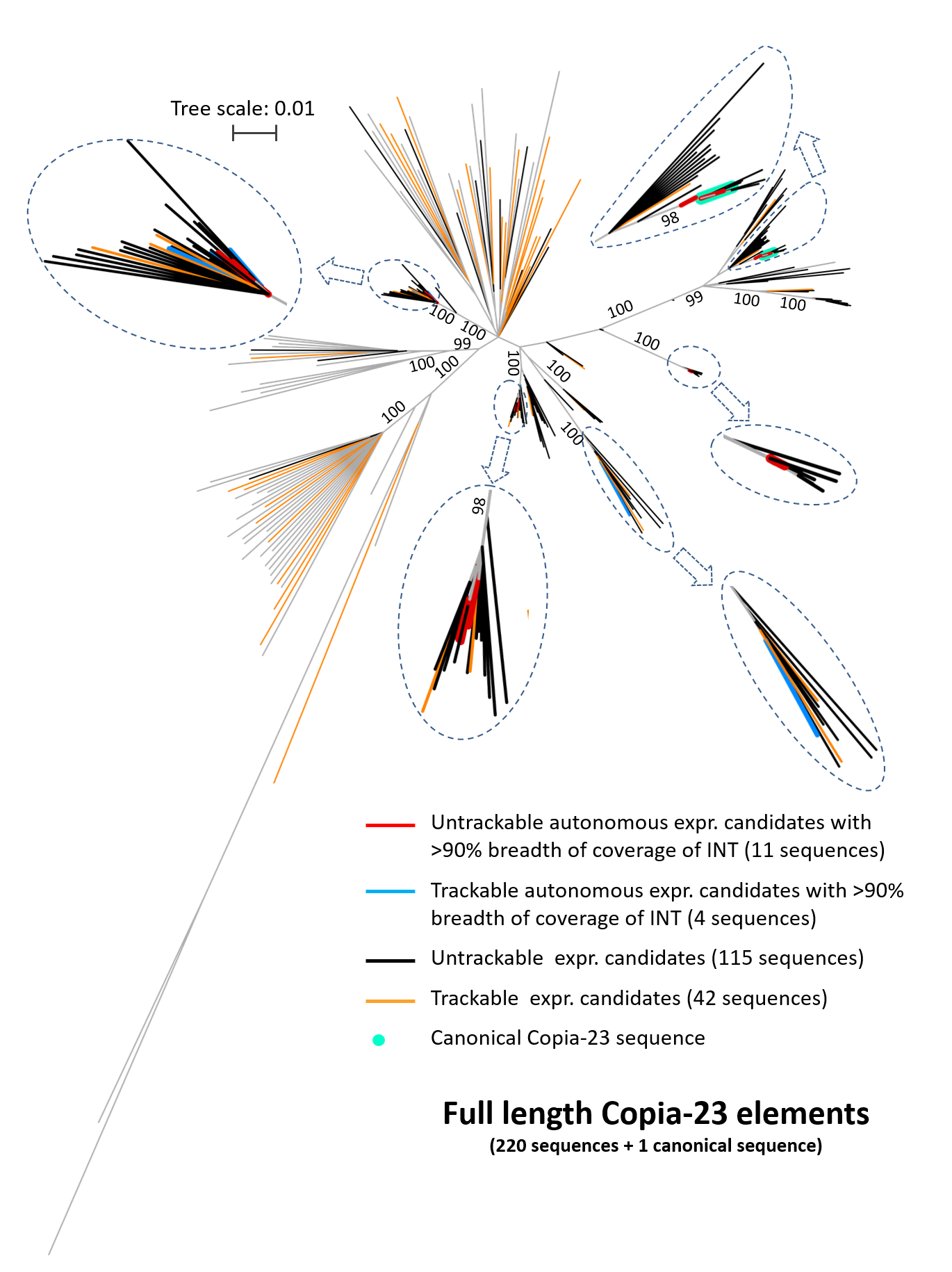


Figure S4. The consensus tree of full-length Copia-23 elements

Copia-23 elements retaining LTR pairs with >90% INT covered by sequencing reads were considered structurally autonomous expression candidates. Blue lines represent those having unique-mapping reads (trackable) blue, and the red lines denote the remaining untrackable ones. For the rest of the full-length Copia-23 expression candidates, those can be distinguished by unique-mapping reads were yellow, otherwise were coloured black. Grey lines denote branches or non-expression candidates. Clades containing trackable or un-trackable autonomous expression candidates were indicated with enlarged illustrations.


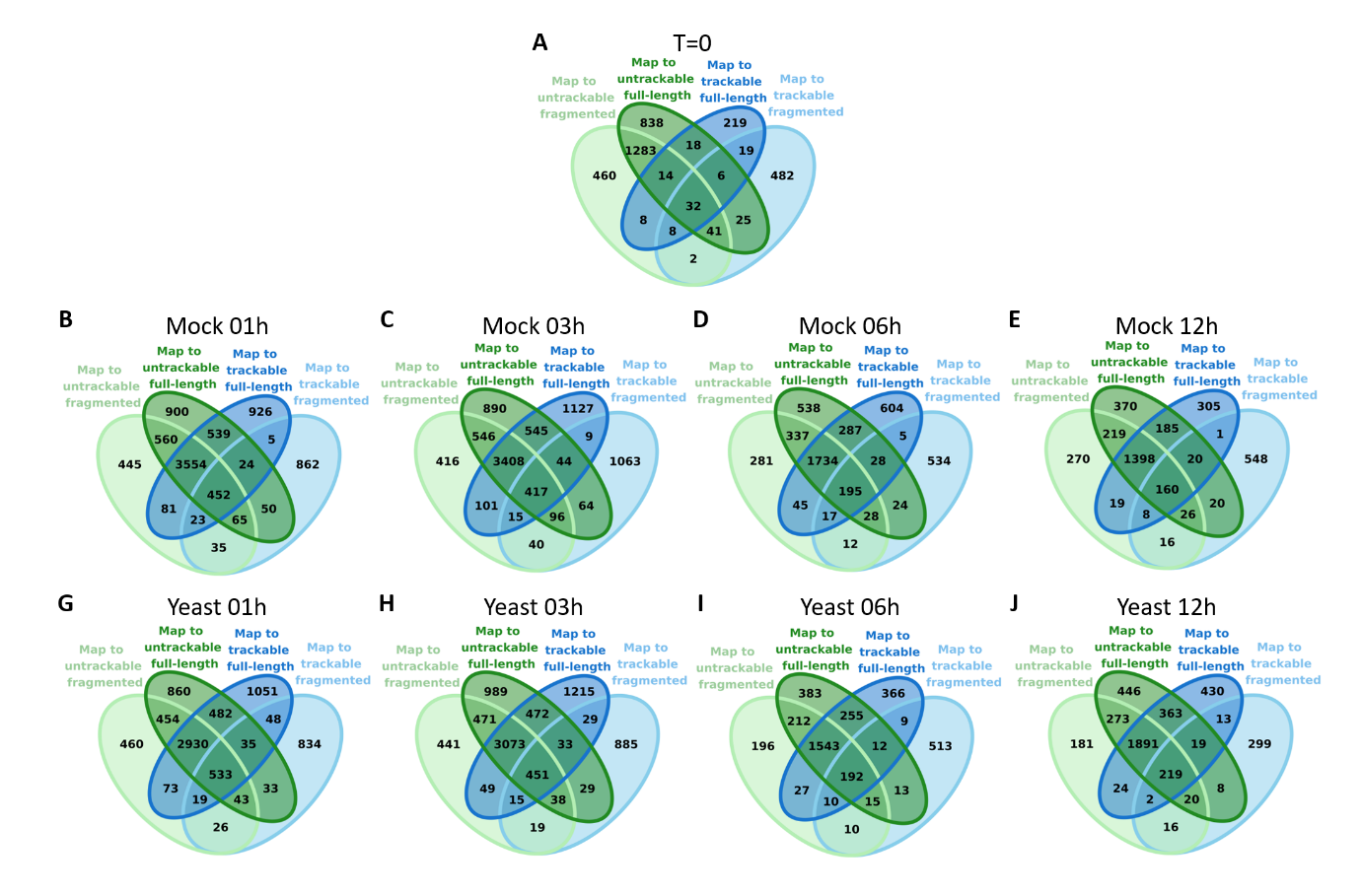


Figure S5. Grouping reads mapping to Copia-3 expression candidates

Reads mapping to Copia-3 expression candidates were categorized into four groups as indicated. Replicates of each time-point were combined.


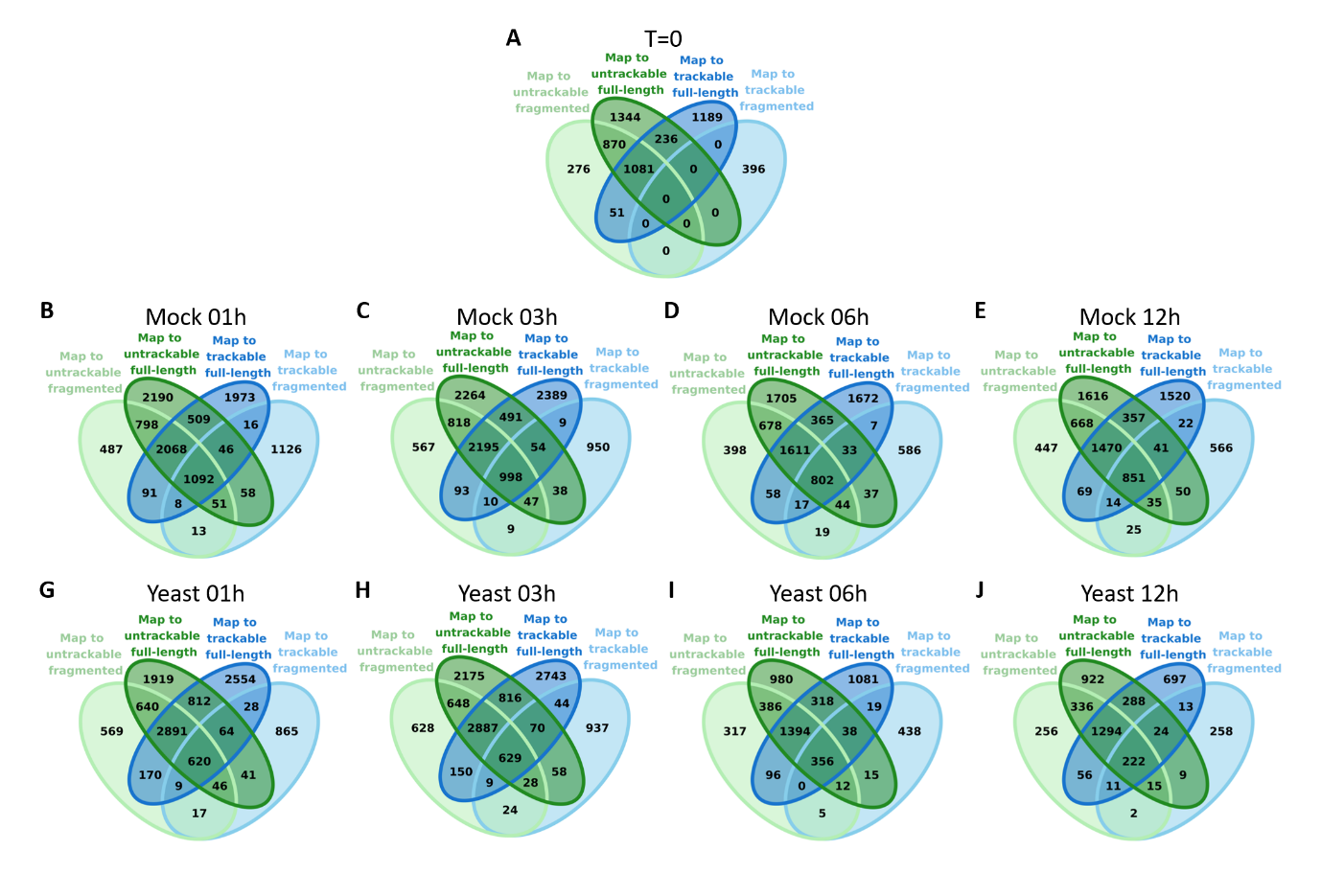


Figure S6. Grouping reads mapping to Copia-23 expression candidates

Reads mapping to Copia-23 expression candidates were categorized into four groups as indicated. Replicates of each time-point were combined.


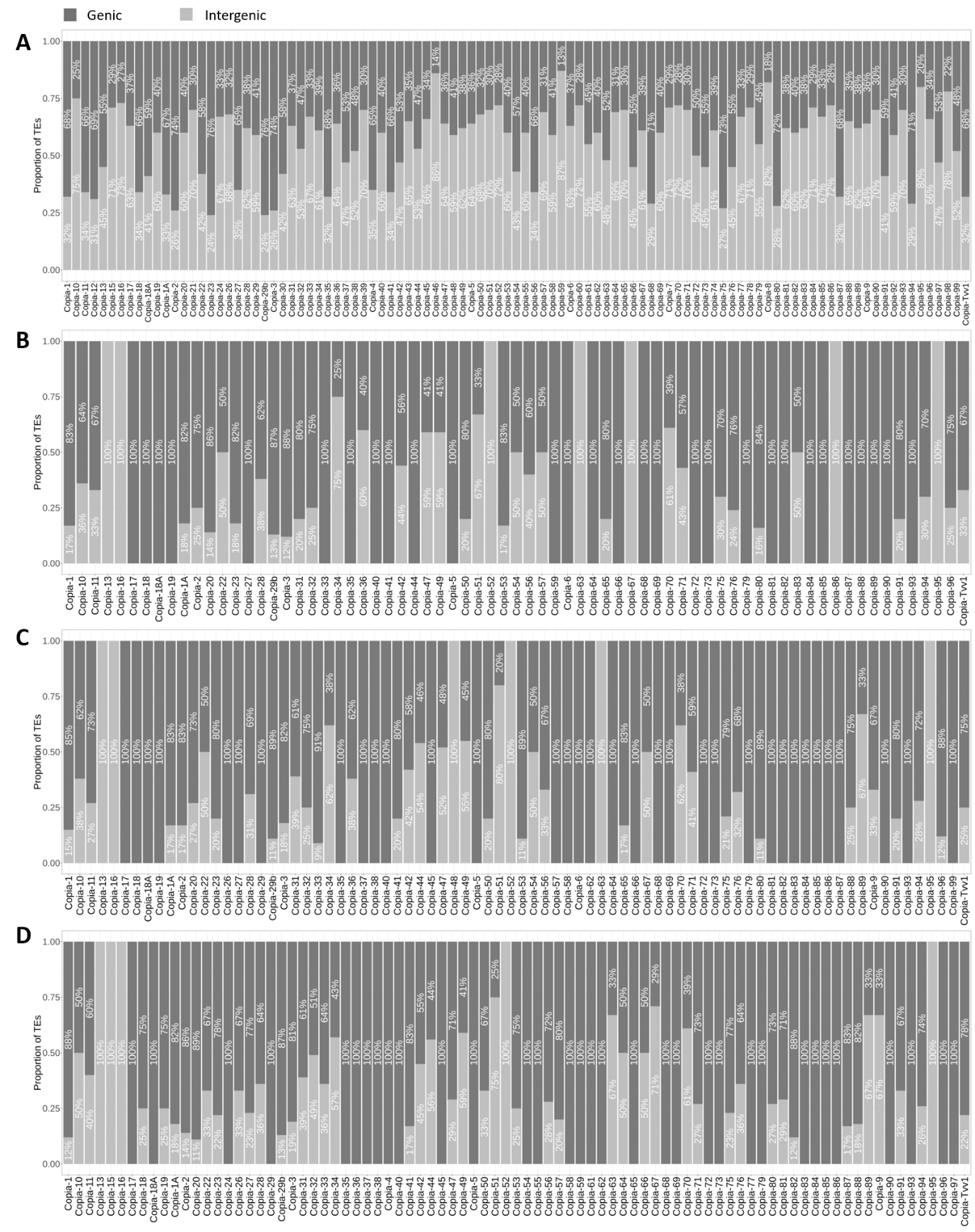


Figure S7. Genic and intergenic distribution of annotated TEs and expression candidates of Copia

**(A)** Distribution of all annotated Copia in the reference genome. **(B-E)** Distribution of Copia expression candidates of Vv_T=0 (B), Vv_Mock (C), and Vv_Yeast (D).


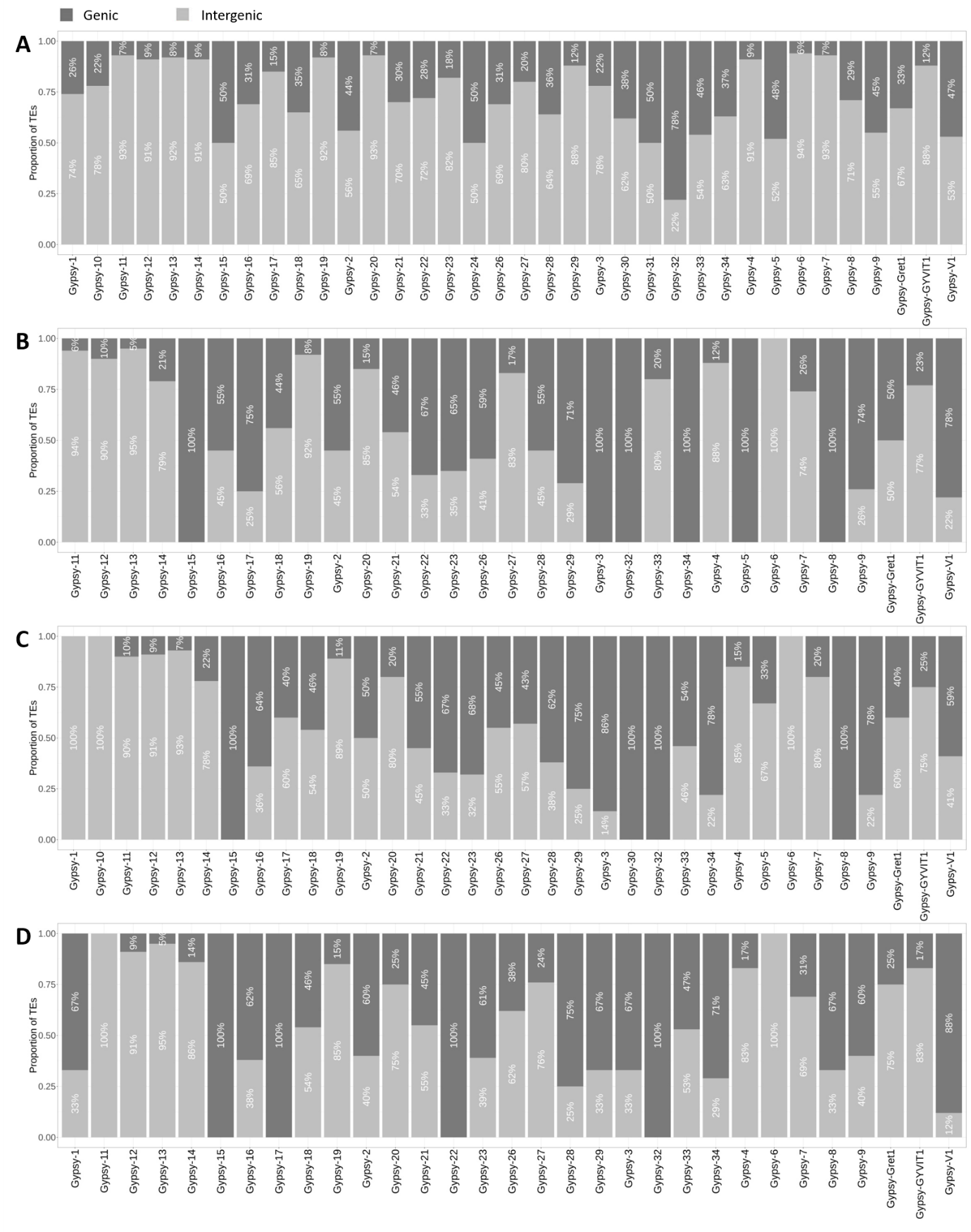


Figure S8. Genic and intergenic distribution of annotated TEs and expression candidates of Gypsy

**(A)** Distribution of all annotated Gypsy in the reference genome. **(B-E)** Distribution of Gypsy expression candidates of Vv_T=0 (B), Vv_Mock (C), and Vv_Yeast (D).


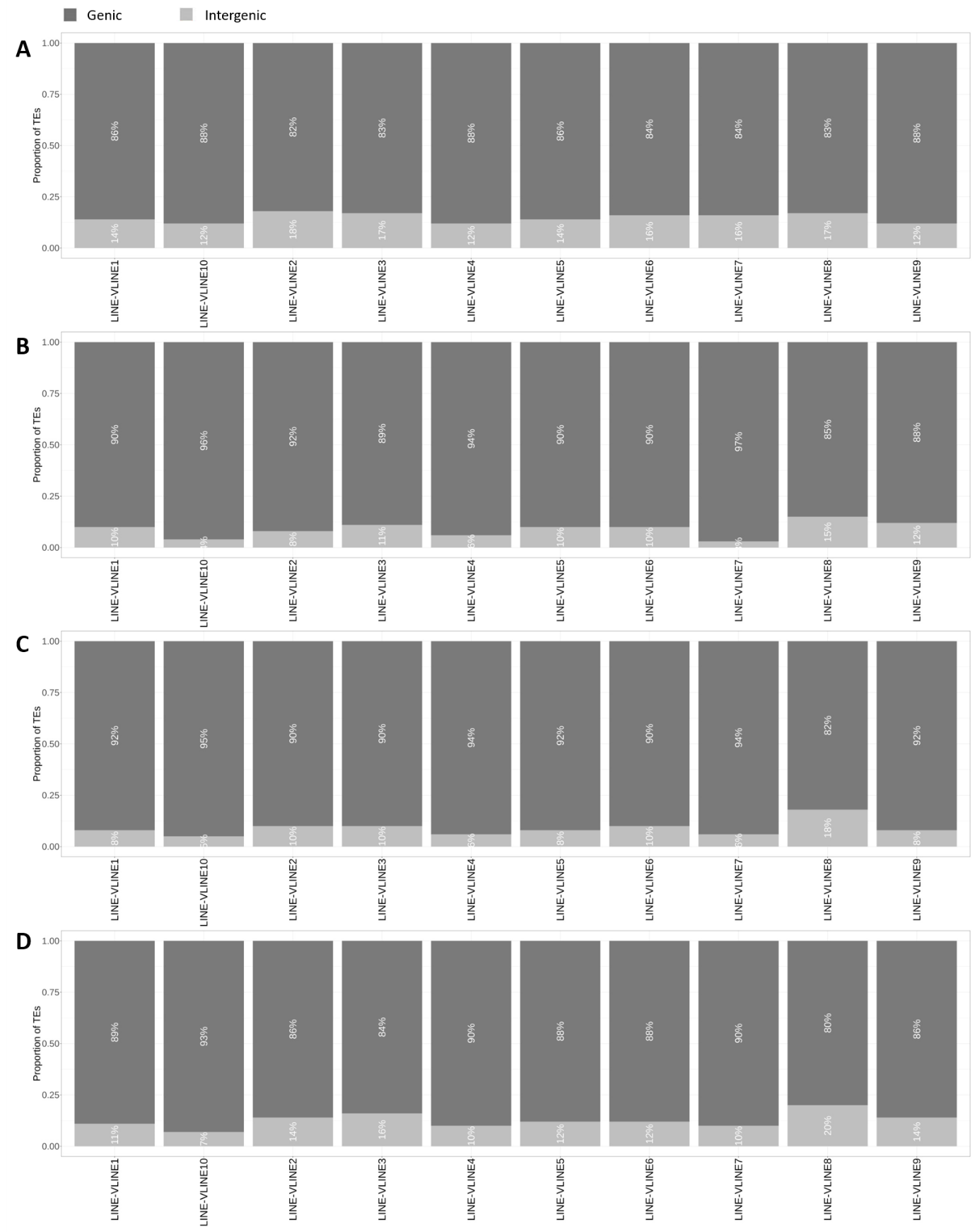


Figure S9. Genic and intergenic distribution of annotated TEs and expression candidates of LINE

**(A)** Distribution of all annotated LINE in the reference genome. **(B-E)** Distribution of LINE expression candidates of Vv_T=0 (B), Vv_Mock (C), and Vv_Yeast (D).


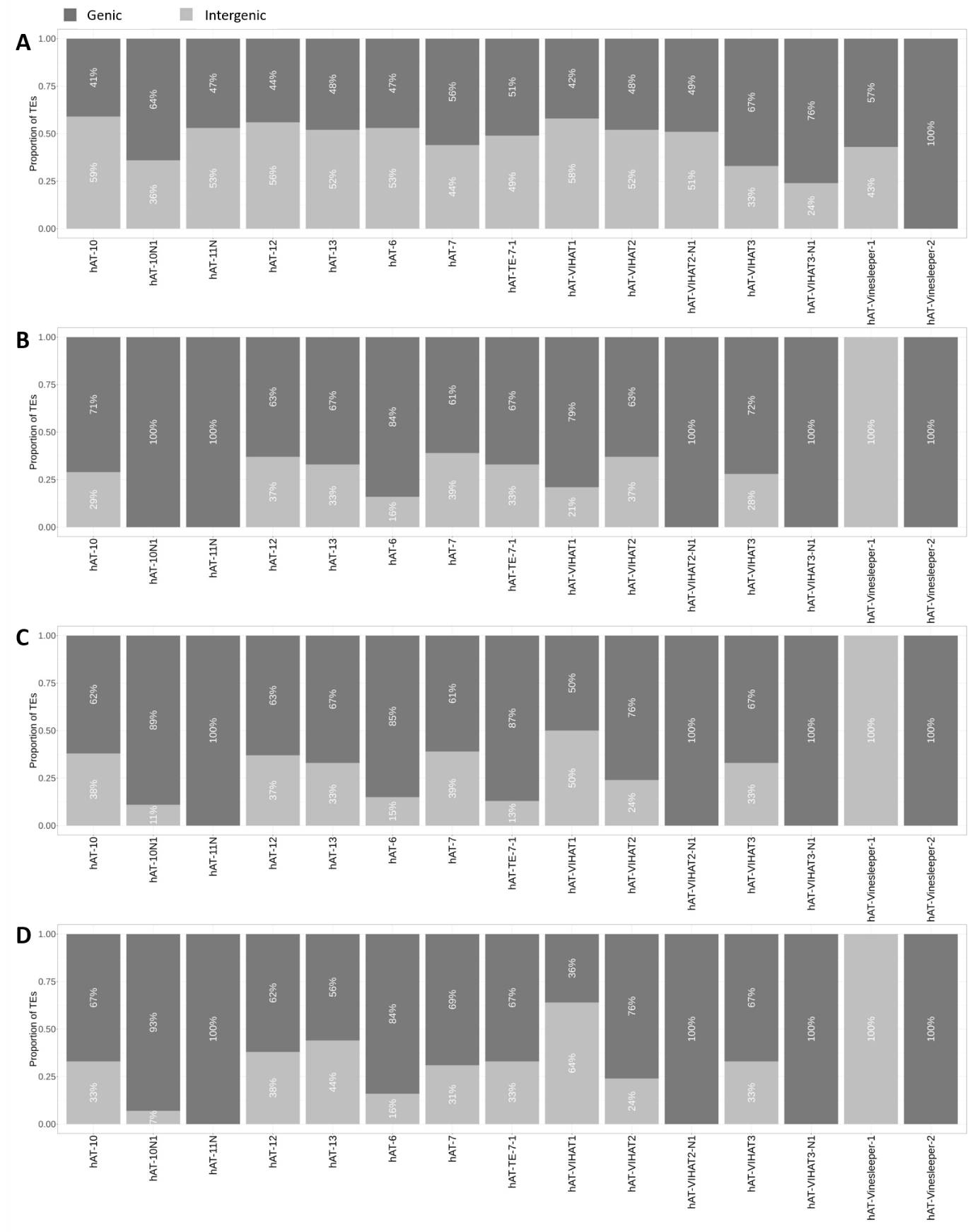


Figure S10. Genic and intergenic distribution of annotated TEs and expression candidates of hAT

**(A)** Distribution of all annotated hAT in the reference genome. **(B-E)** Distribution of hAT expression candidates of Vv_T=0 (B), Vv_Mock (C), and Vv_Yeast (D).


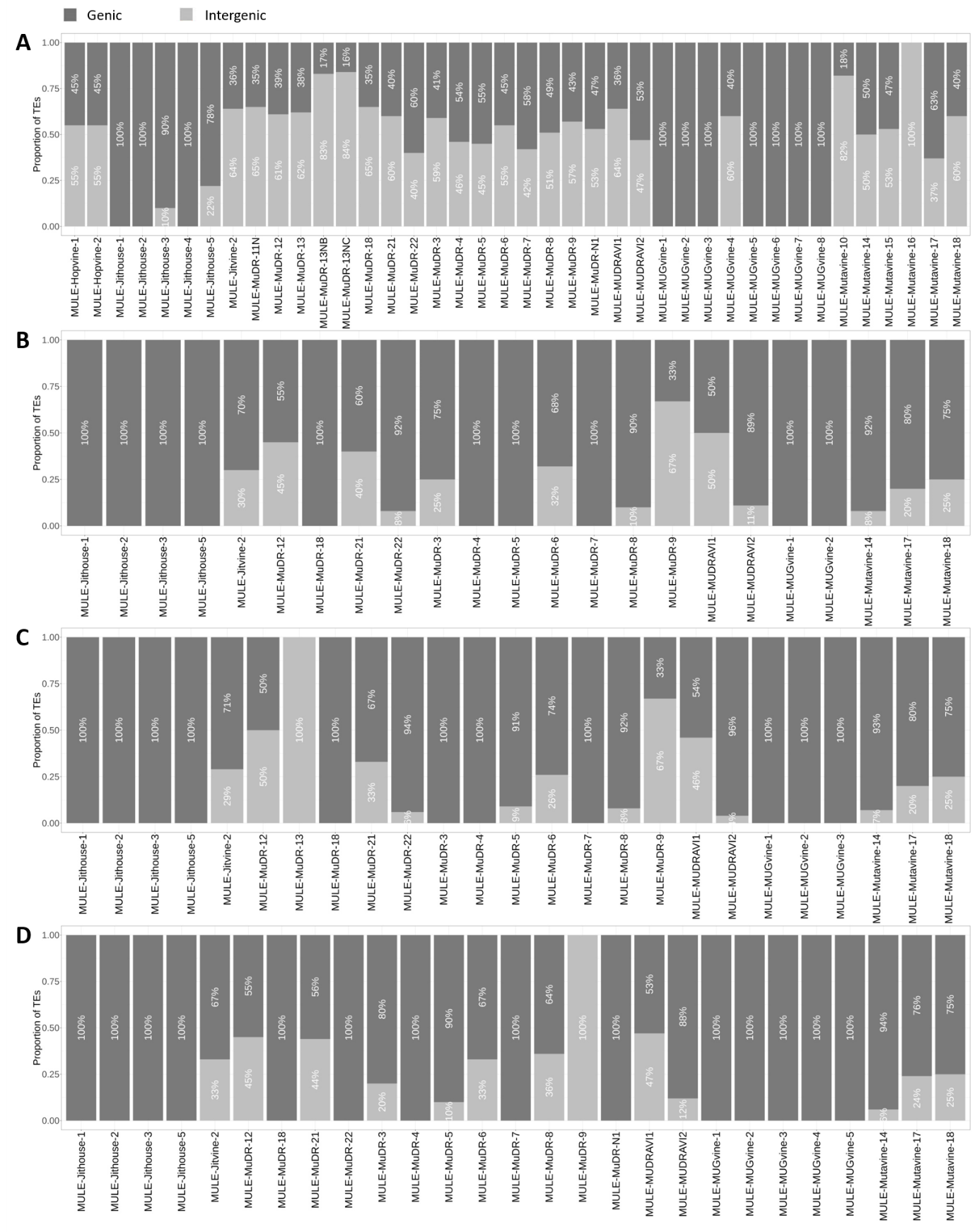


Figure S11. Genic and intergenic distribution of annotated TEs and expression candidates of MULE

**(A)** Distribution of all annotated MULE in the reference genome. **(B-E)** Distribution of MULE expression candidates of Vv_T=0 (B), Vv_Mock (C), and Vv_Yeast (D).


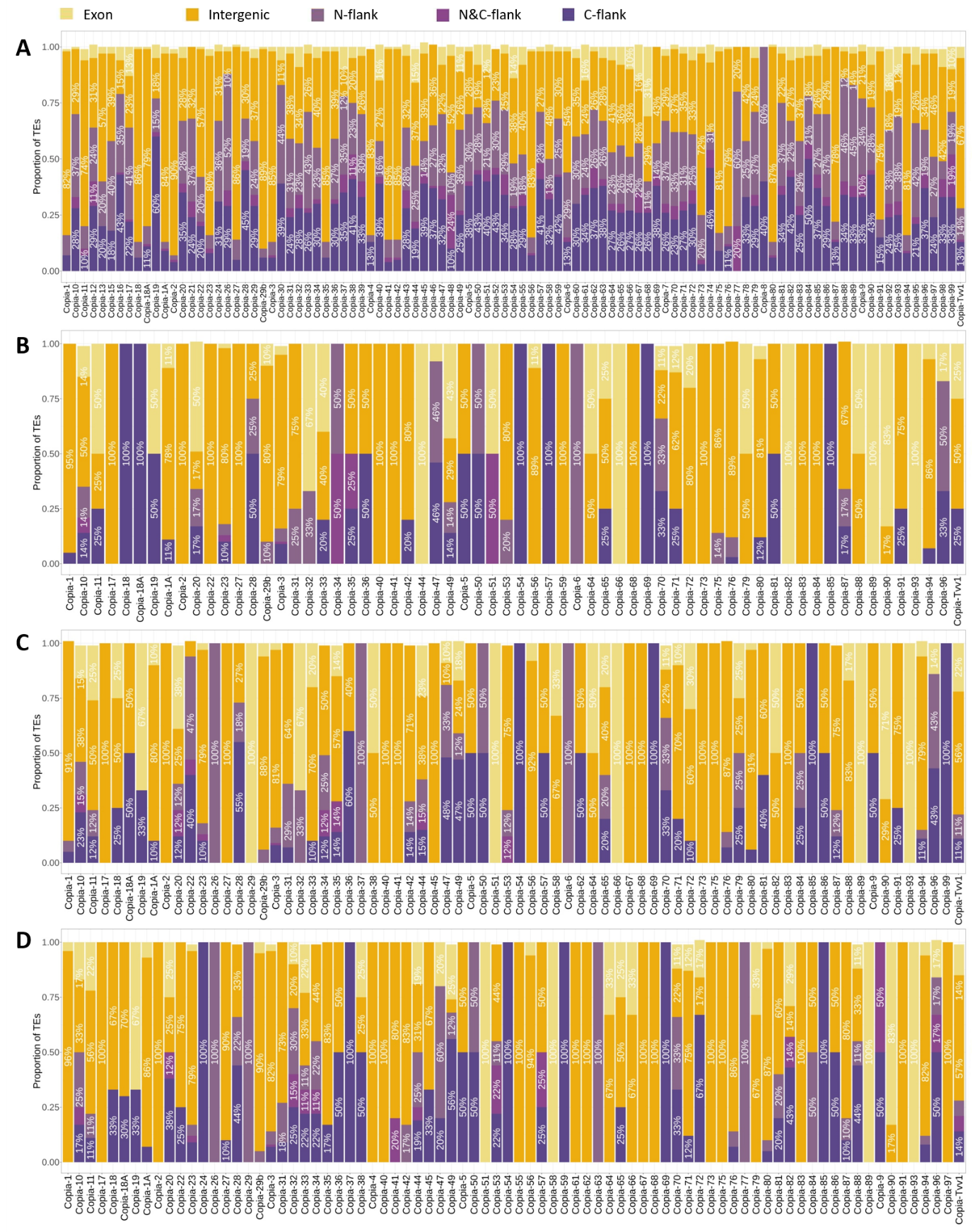


Figure S12. Location distribution of annotated genic TEs and expression candidates of Copia

**(A)** Location distribution of all annotated genic Copia in the reference genome. **(B-E)** Location distribution of genic Copia expression candidates of Vv_T=0 (B), Vv_Mock (C), and Vv_Yeast (D).


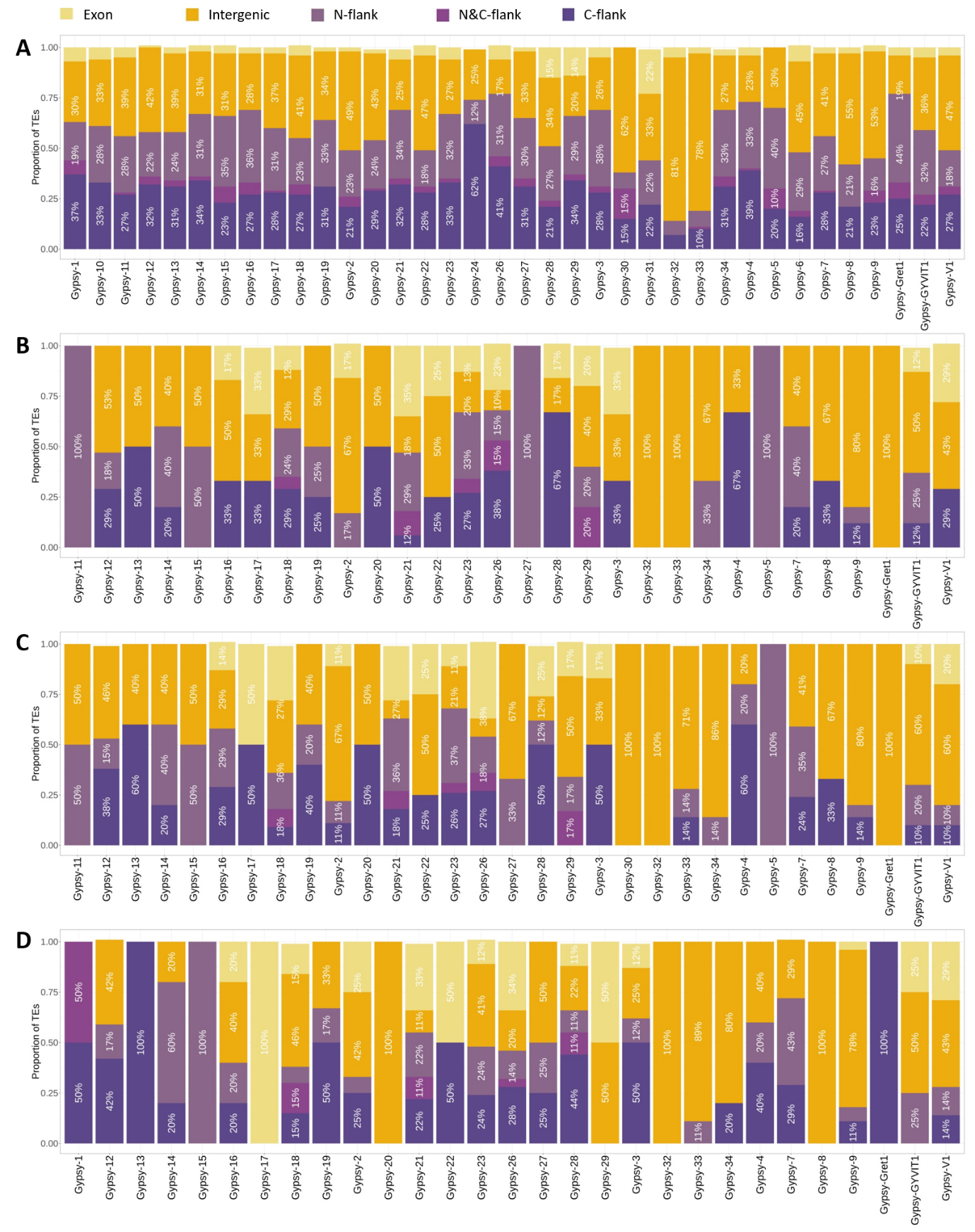


Figure S13. Location distribution of annotated genic TEs and expression candidates of Gypsy

**(A)** Location distribution of all annotated genic Gypsy in the reference genome. **(B-E)** Location distribution of genic Gypsy expression candidates of Vv_T=0 (B), Vv_Mock (C), and Vv_Yeast (D).


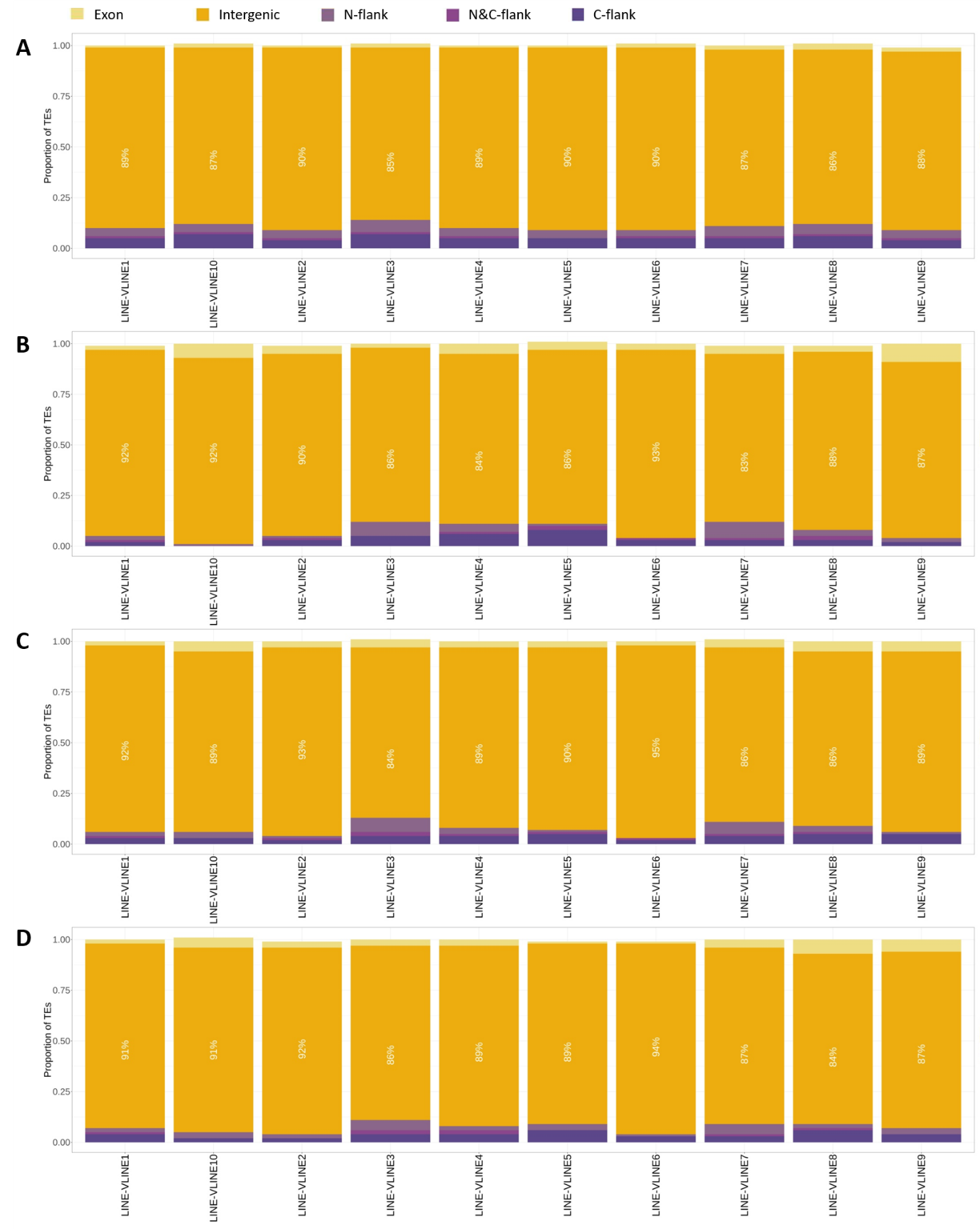


Figure S14. Location distribution of annotated genic TEs and expression candidates of LINE

**(A)** Location distribution of all annotated genic LINE in the reference genome. **(B-E)** Location distribution of genic LINE expression candidates of Vv_T=0 (B), Vv_Mock (C), and Vv_Yeast (D).


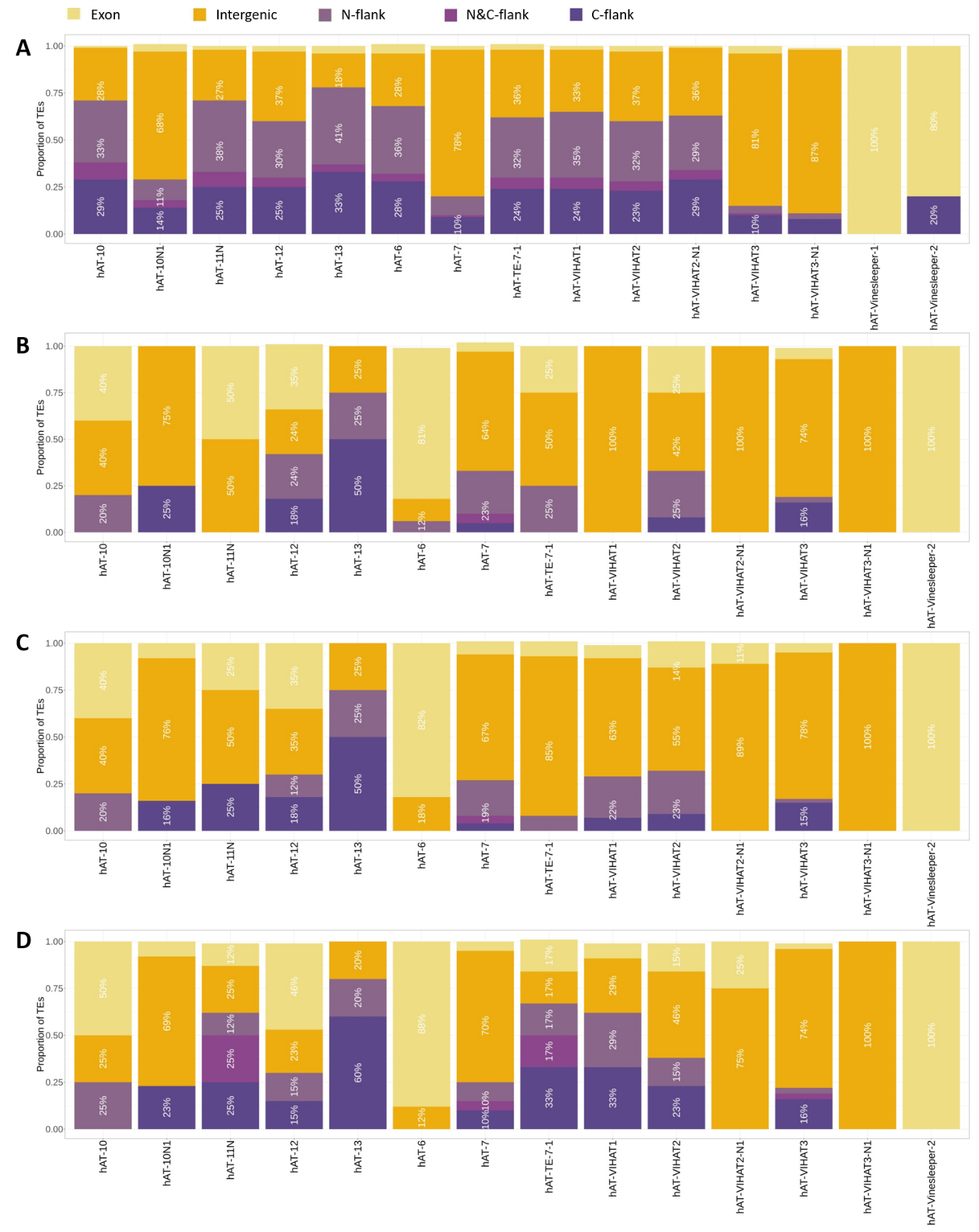


Figure S15. Location distribution of annotated genic TEs and expression candidates of hAT

**(A)** Location distribution of all annotated genic hAT in the reference genome. **(B-E)** Location distribution of genic hAT expression candidates of Vv_T=0 (B), Vv_Mock (C), and Vv_Yeast (D).


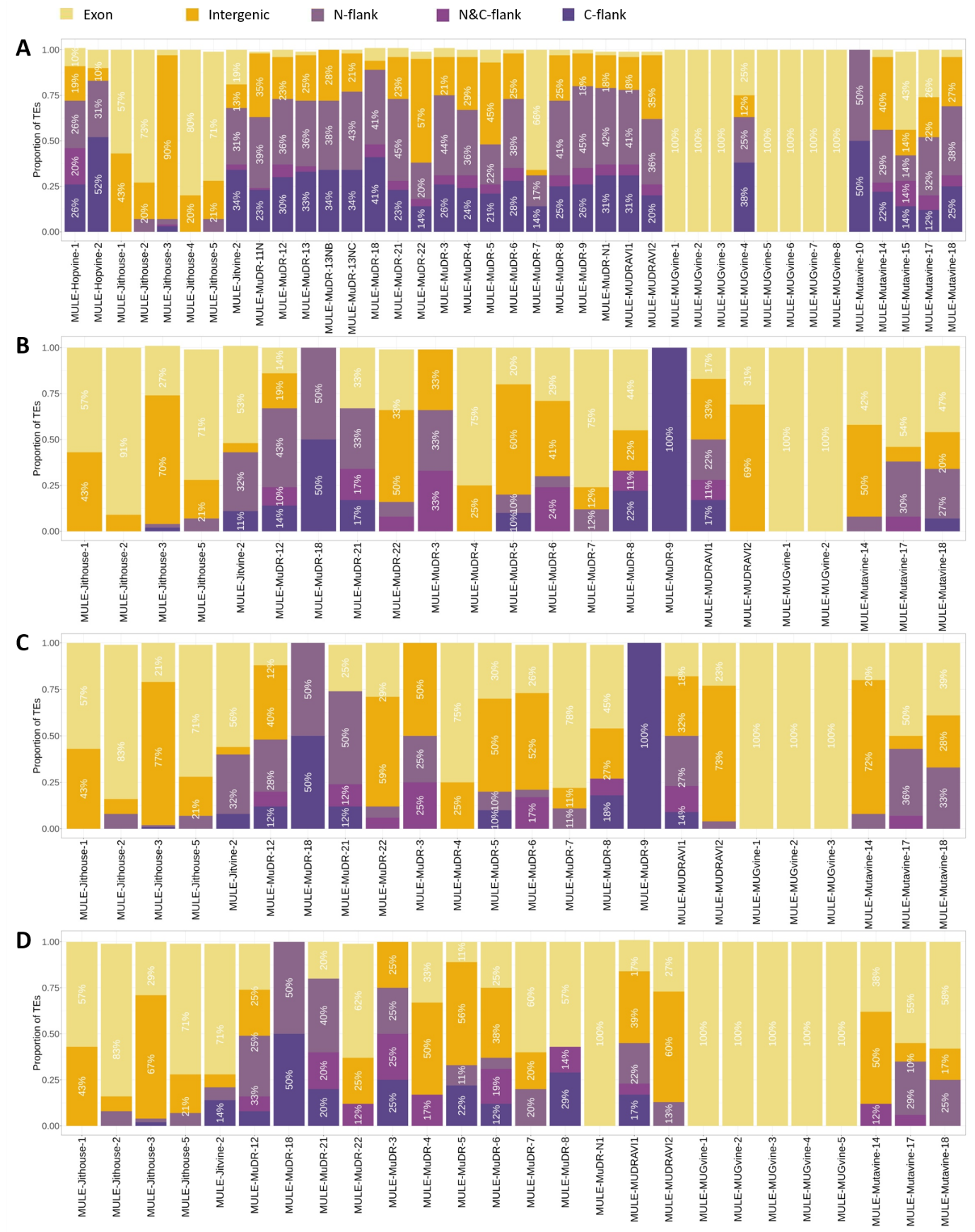


Figure S16. Location distribution of annotated genic TEs and expression candidates of MULE

**(A)** Location distribution of all annotated genic MULE in the reference genome. **(B-E)** Location distribution of genic MULE expression candidates of Vv_T=0 (B), Vv_Mock (C), and Vv_Yeast (D).


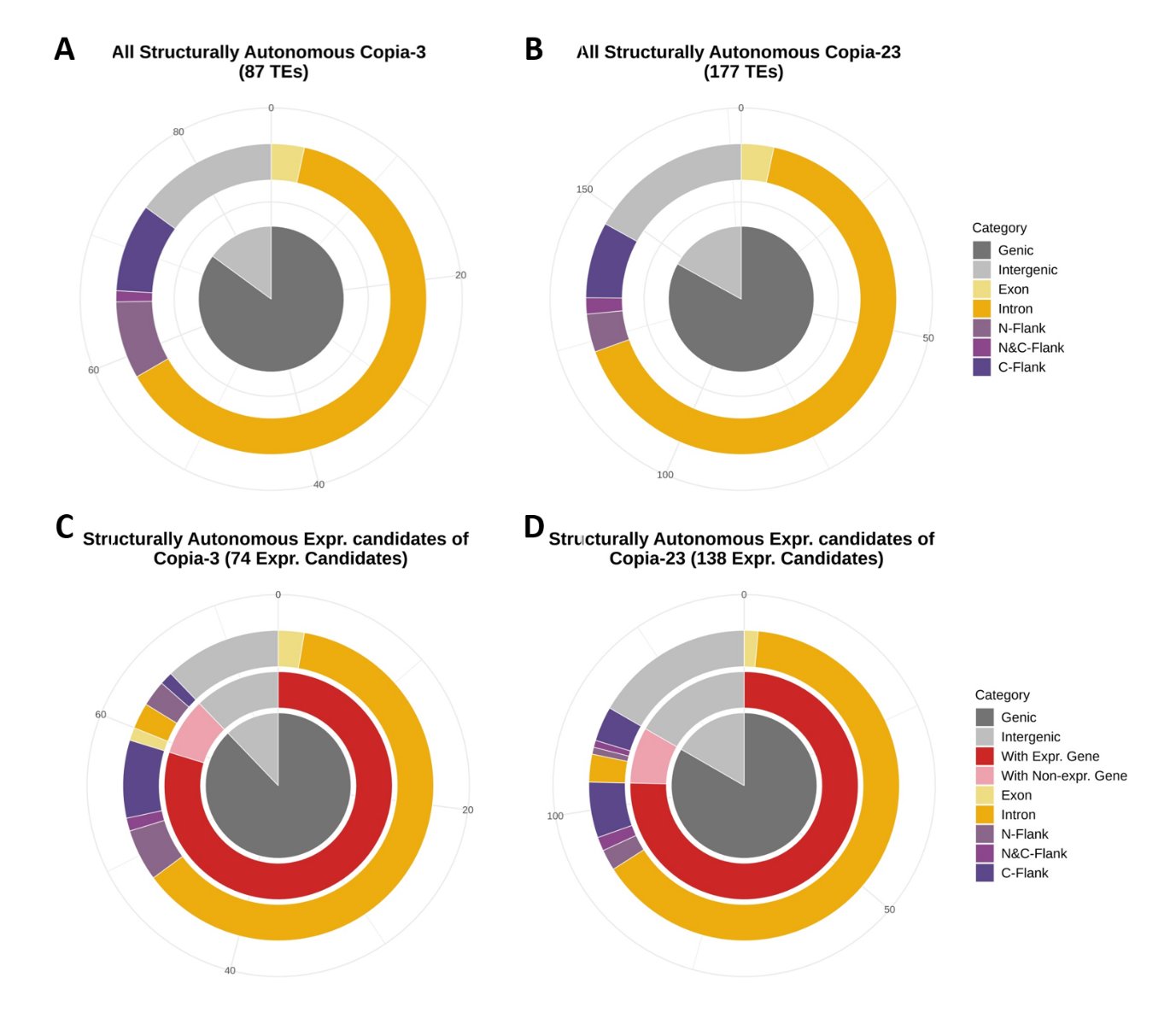


Figure S17. Hierarchical classifications of structurally intact Copia-3 and Copia-23 by location relative to gene.

**(A-B)** All structurally intact TE loci of Copia-3 (A) and Copia-23 (B) were categorized hierarchically by region (centre) and location. **(C-D)** Structurally autonomous expression candidates of Copia-3 (C) and Copia-23 (D) each treatment were categorized in the order of region (centre), the transcriptional activity of co-localized genes (2^nd^ layer), and location (3^rd^ layer). These include TE loci that were identified as expression candidates in at least one of the three experimental conditions (Vv_T=0, Vv_Mock, and Vv_Yeast).


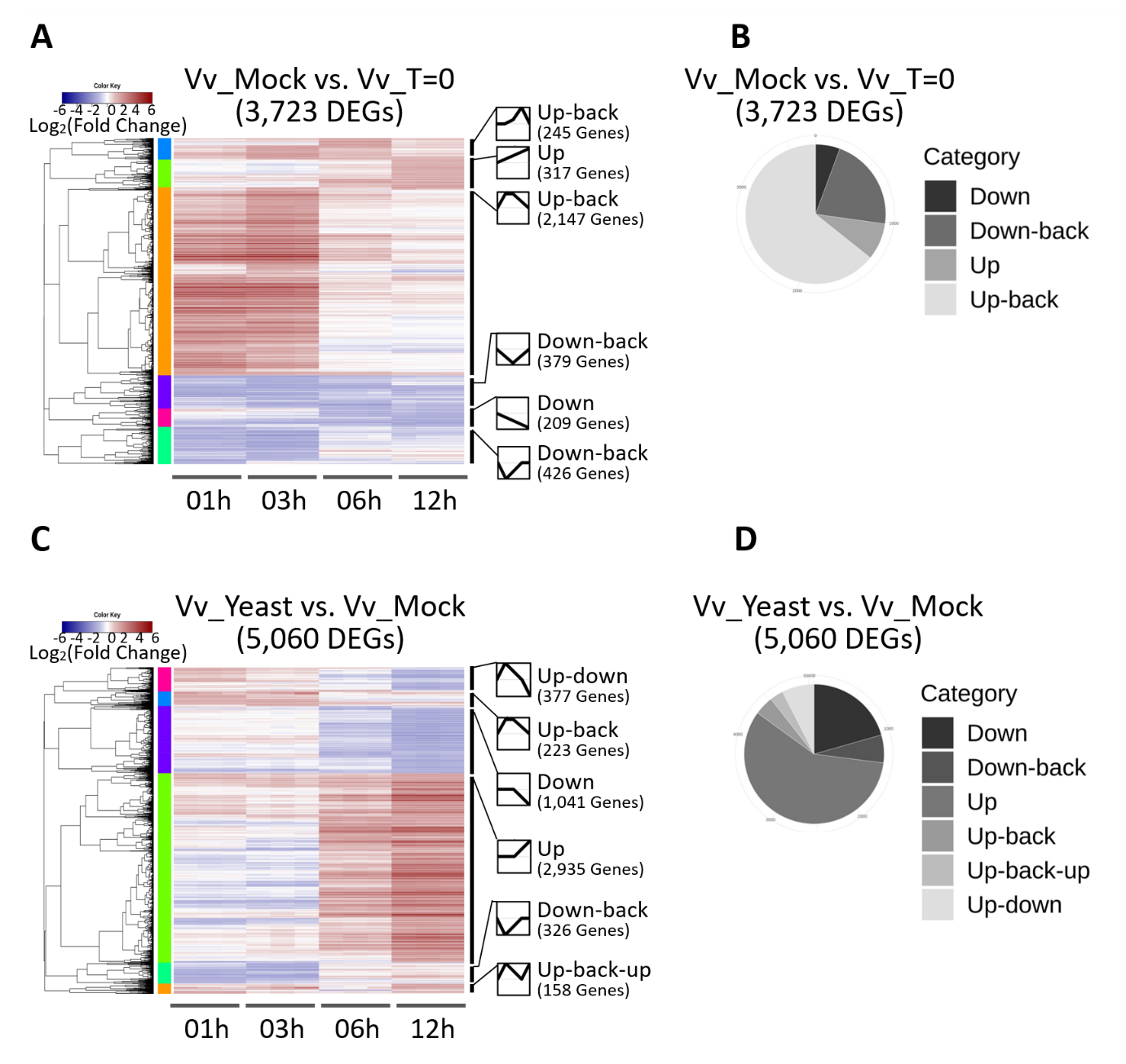


Figure S18. Expression patterns of DEGs

**(A, C)** DEGs in Vv_Mock vs Vv_T=0 (A) and Vv_Yeast vs Vv_Mock (C) were illustrated by heatmaps log_2_(fold change) with line graphs representing the dynamics of each cluster across time. **(B, D)** Similar clusters were then grouped together for the pie graphs summarizing the trend of expression changes in Vv_Mock vs Vv_T=0 (B) and Vv_Yeast vs Vv_Mock (D).


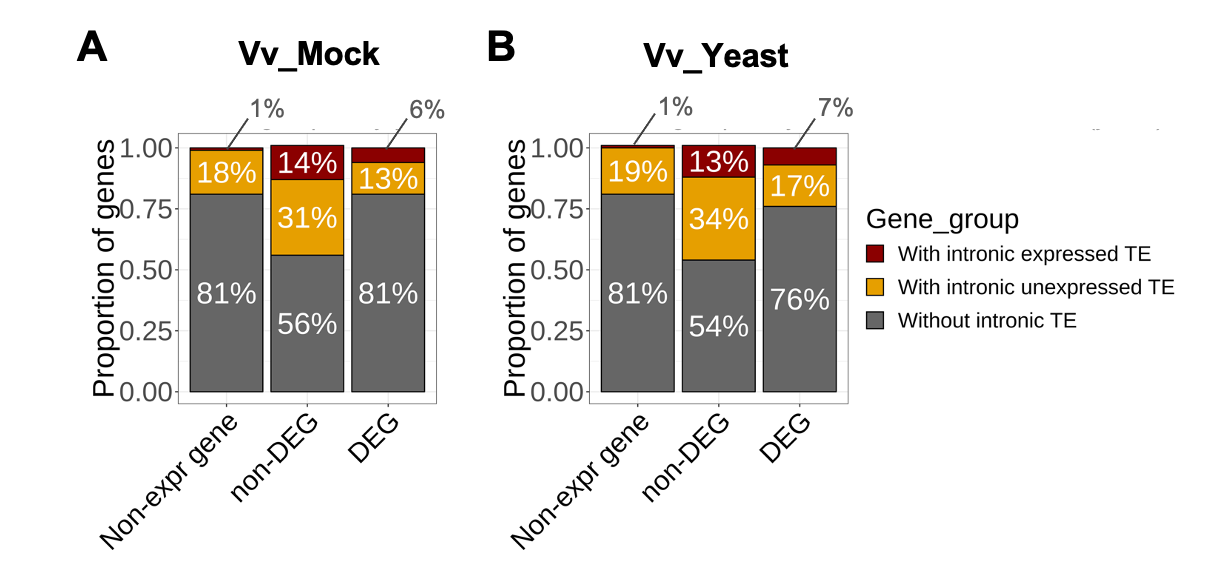


**Figure S19. Grapevine genes grouped by presence of intronic TEs**

All annotated grapevine genes were firstly categorised into non-expressed gene (non-expr gene), non-differentially expressed genes (non-DEG), and DEG by their expression activity in **(A)** Vv_Mock and **(B)** Vv_Yeast. These gene were then separate into three subgroups: with intronic expressed TEs, with intronic unexpressed TEs, or without intronic TEs. The proportion of genes in each subgroup was as indicated.


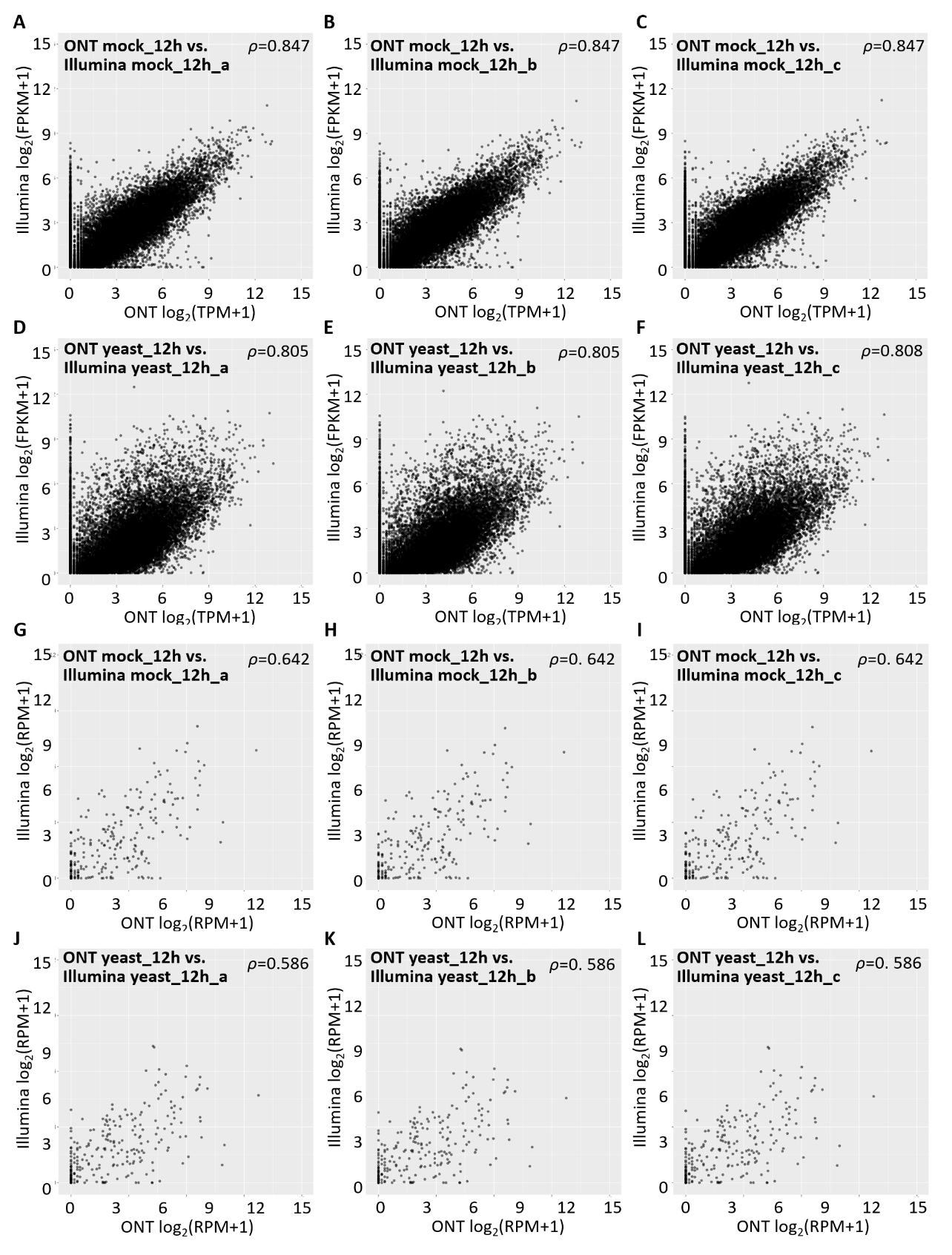


**Figure S20. Comparisons between gene expression quantified from ONT and Illumina Truseq sequencing libraries**

**(A-C, G-I)** For mock (12 hours) treatment, gene (A-C) and TE family (G-I) expression level quantified from the ONT library was compared to each of the replicates sequenced by Illumina RNAseq. **(D-F, J-L)** For yeast (12 hours) treatment, the ONT library was compared to each of the libraries sequenced by Illumina RNAseq. The gene expression levels (A-F) were given as transcripts per million mapped reads (TPM) for the ONT libraries (x-axes) and as fragments per kilobase per million mapped reads (FPKM) for the Illumina libraries (y-axes). The TE expression levels (G-L) were given as reads per million mapped reads (RPM) for both sequencing methods family (x-axes=ONT and y-axes=Illumina). Spearman’s correlation coefficient ρ was given per comparison with each point representing gene expression levels.


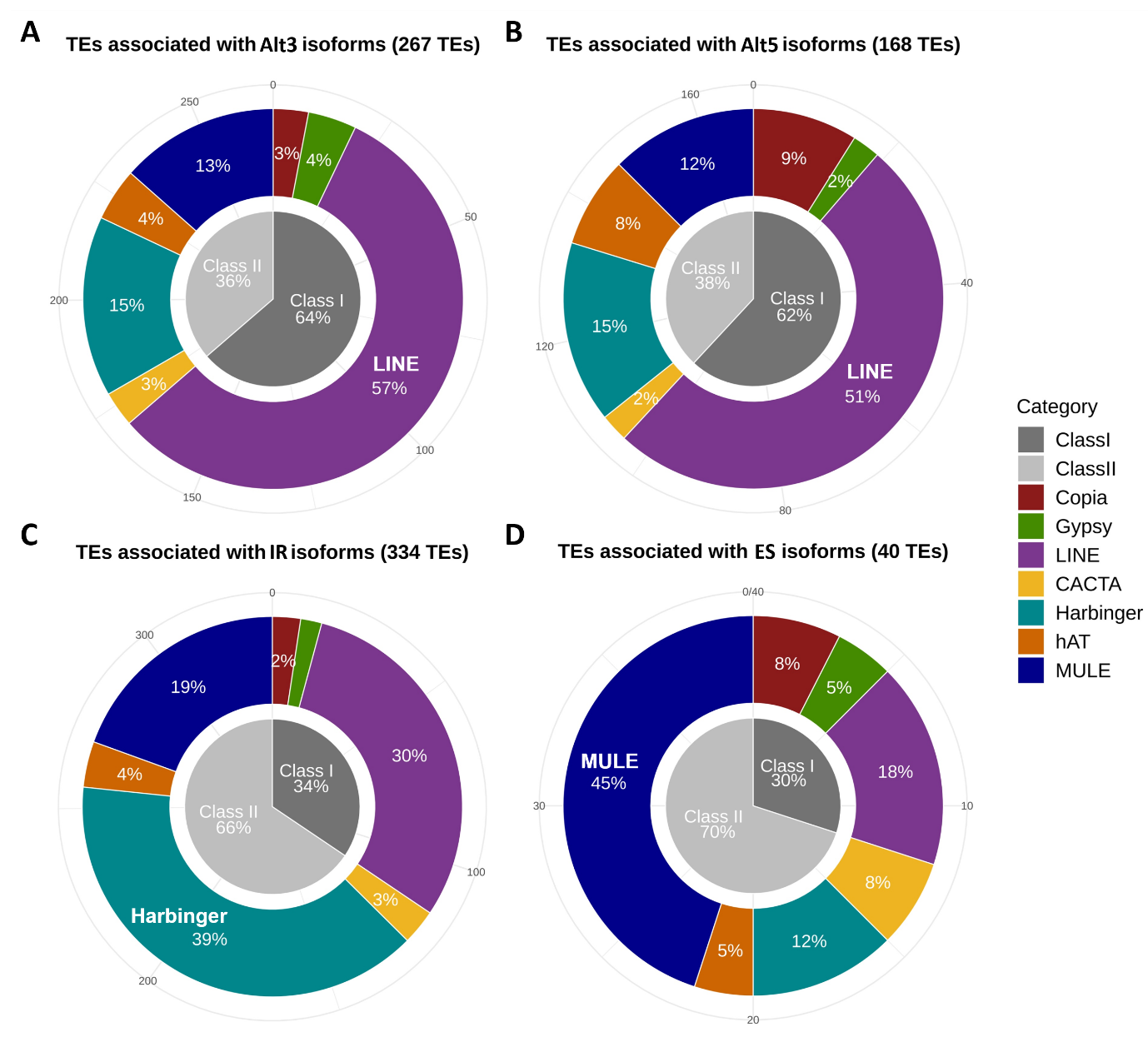


Figure S21. Categorization of TEs associated with alternative splicing

TE loci overlapping with gene-related **(A)** Alt3, **(B)** Alt5, **(C)** IR, and **(D)** ES features were grouped by class (central pie graph), then by superfamily (outer doughnut graph). The percentage of each slice is as indicated, with the most over-represented superfamily labelled.


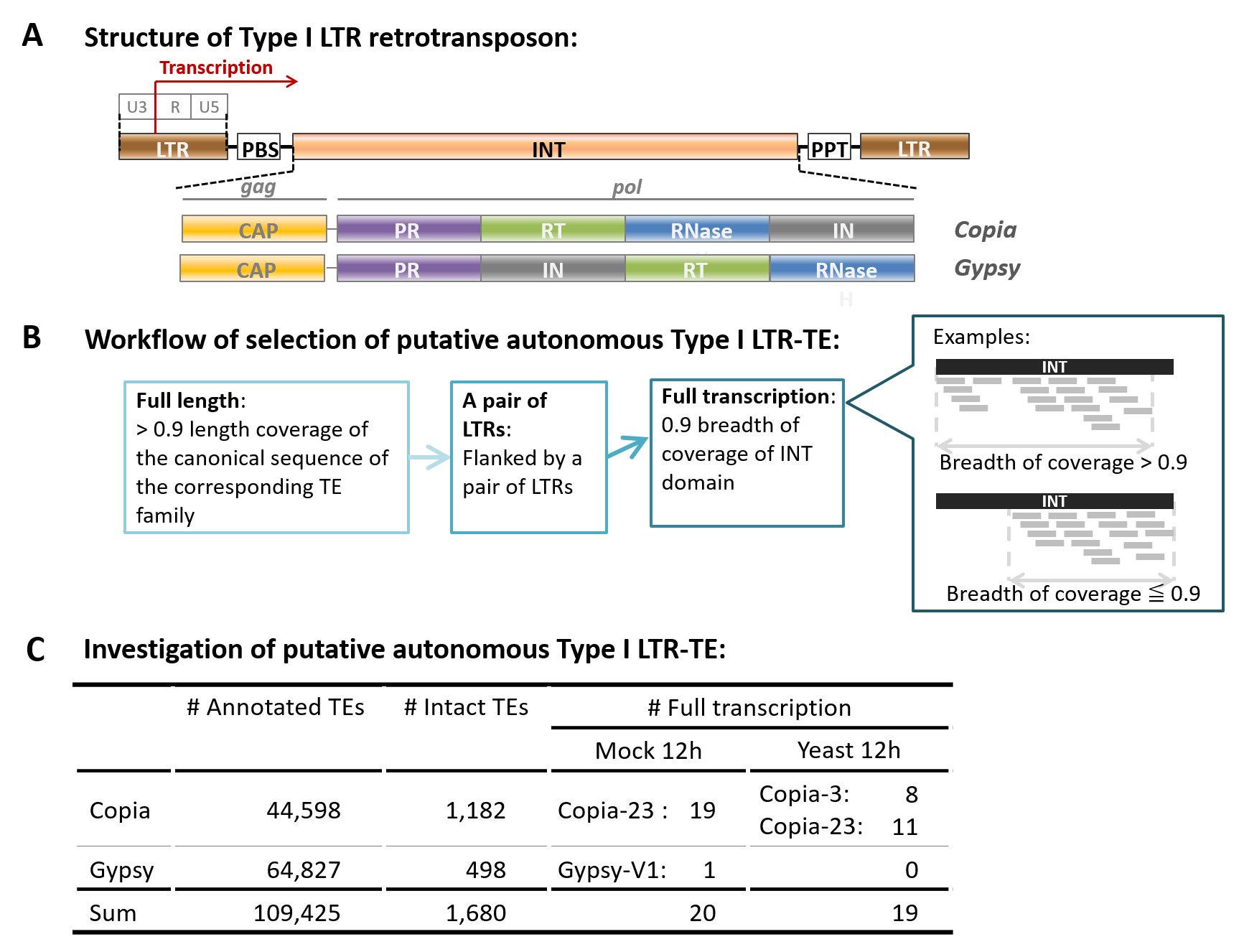


Figure S22. Identification of autonomous LTR-TE with potential full-transcription.

**(A)** Autonomous LTR-TEs are characterized by the poly-protein-coding internal domain (INT) flanked by a pair of long terminal repeats (LTR). The transcription starts within the 5’ LTR, going through the INT domain, and generally terminated at 3’ LTR. See Figure 4.1 for the acronyms. The diagram is not drawn to scale. **(B)** Workflow for collecting autonomous LTR-TE loci that were potentially fully transcribed. The short grey segments denote sequencing reads. **(C)** The numbers of annotated, intact, and potentially fully transcribed LTR-TE loci.


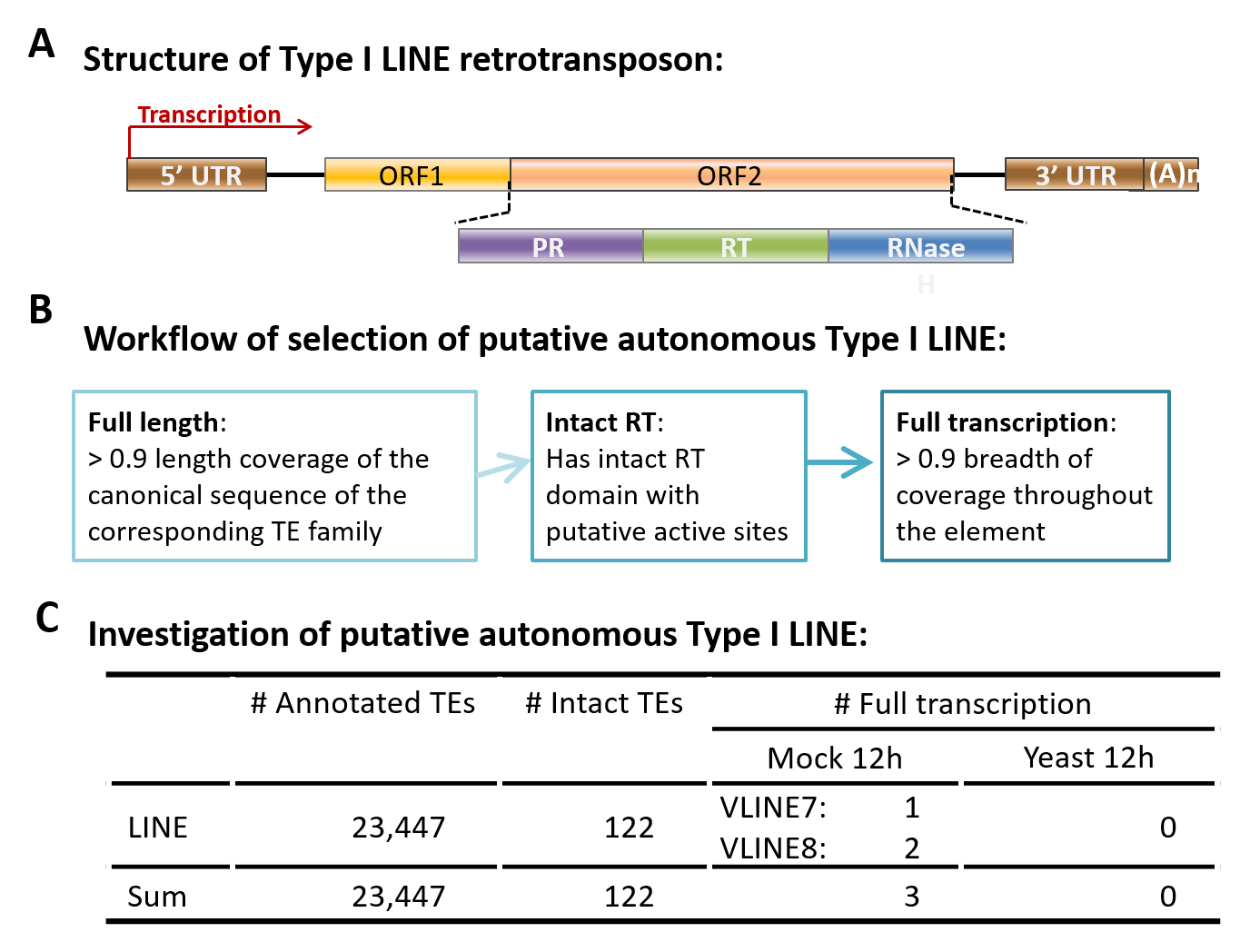


Figure S23. Identification of autonomous LINE with potential full-transcription.

**(A)** An autonomous LINE is expected to retain the open reading frames (ORF) encoding proteins necessary for mobilization. Typically, the transcription starts from the 5’ untranslated region (5’ UTR), going through ORFs and 3’ UTR, and completed with polyadenylation. See Figure 4.2 for the acronyms. The diagram is not drawn to scale. **(B)** Workflow for collecting autonomous LINE loci that were potentially fully transcribed. **(C)** The numbers of annotated, intact, and potentially fully transcribed LINE loci.


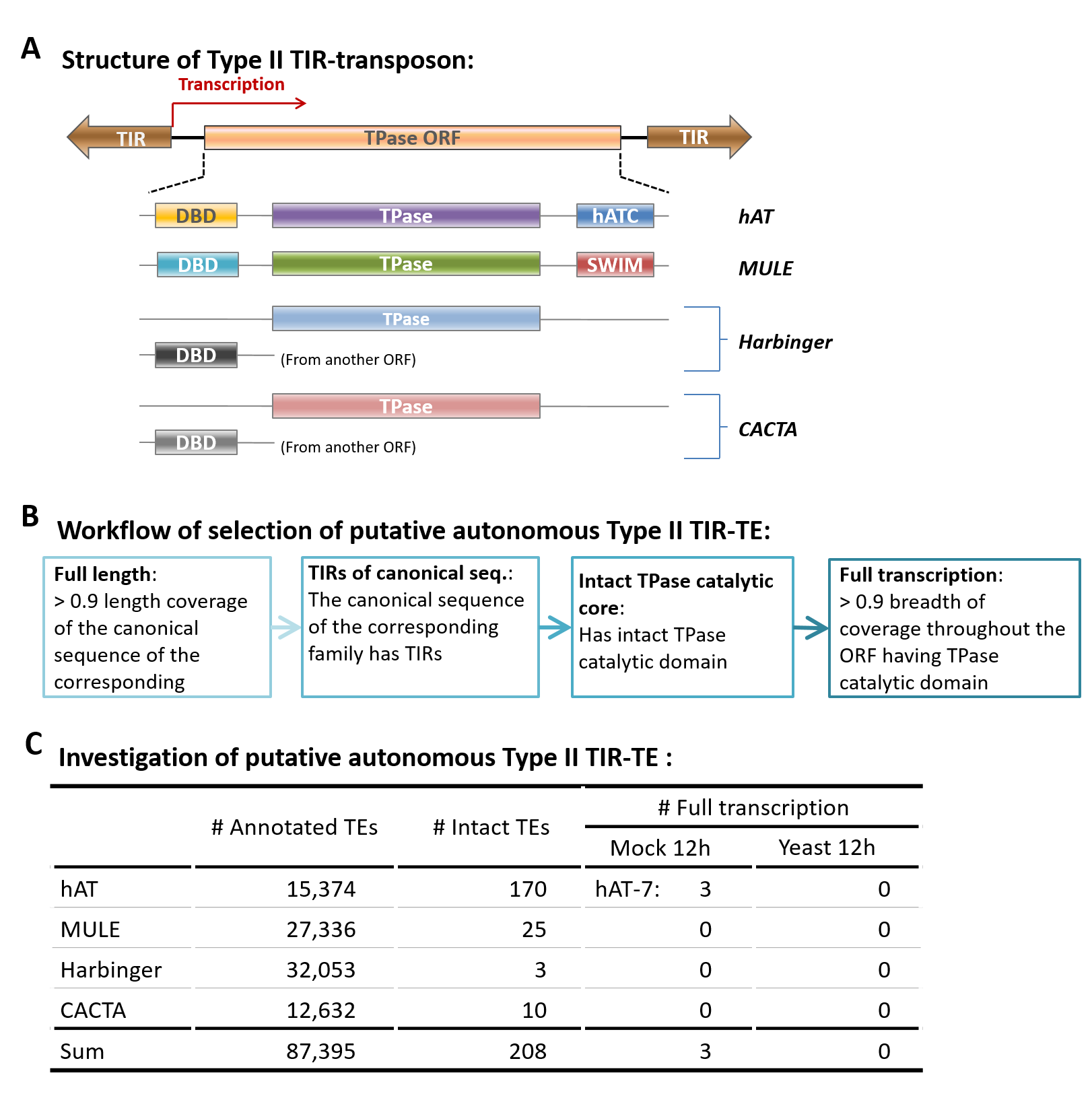


Figure S24. Identification of autonomous TIR-TEs with potential full-transcription.

**(A)** An autonomous TIR-TE is supposedly equipped with a transposase (TPase)-encoding ORF flanked by terminal inverted repeats (TIR). The typical transcription start site is as indicated. See Figure 4.3 for the acronyms. The diagram is not drawn to scale. **(B)** Workflow for collecting autonomous TIR-TE loci that were potentially fully transcribed. **(C)** The number of annotated, intact, and potentially fully transcribed TIR-TE loci.


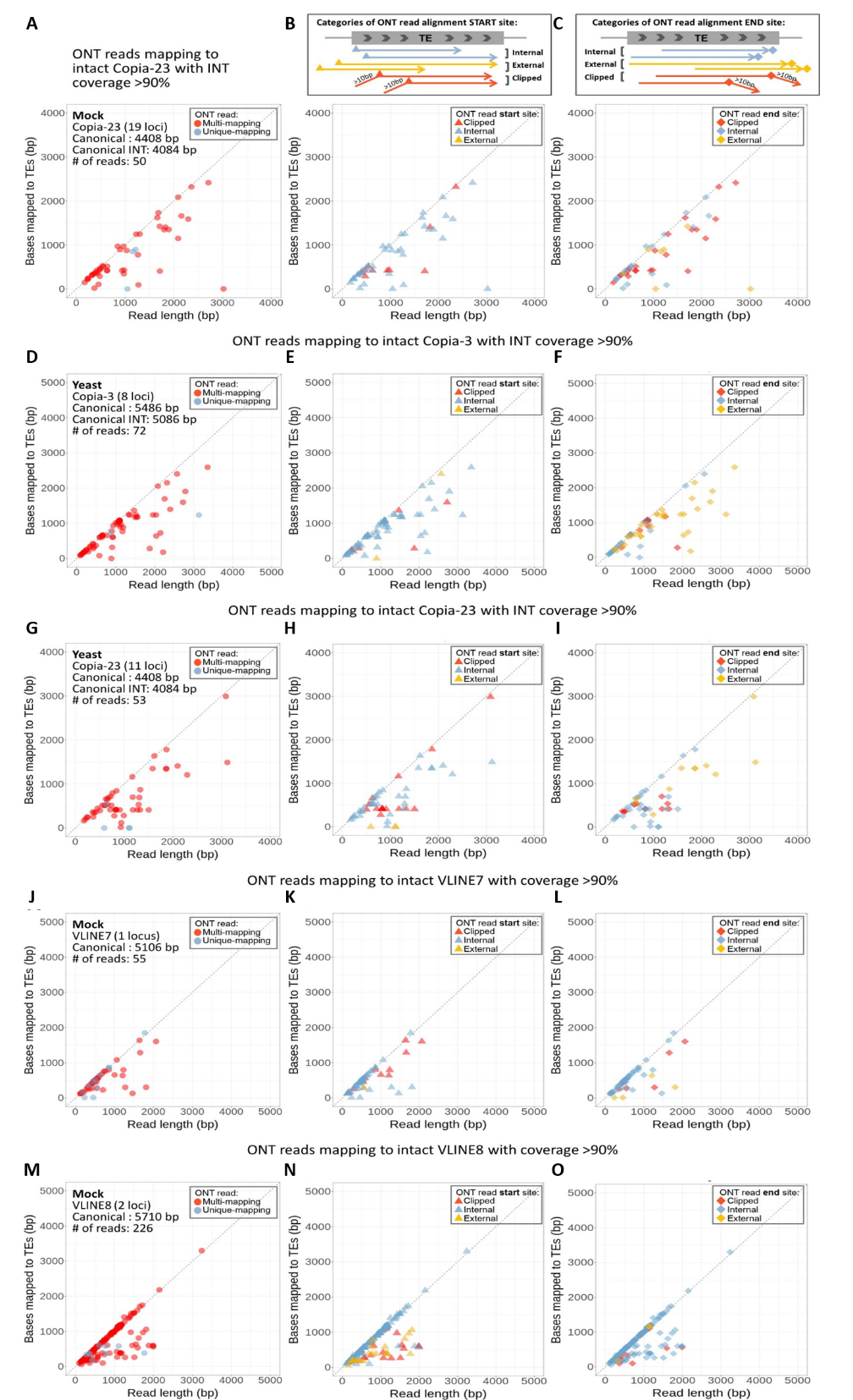


Figure S25. Characteristics of ONT reads mapping to structurally intact TE loci identified by the workflow shown in Figure S22-23.

**(A-C)** Scatter plots demonstrating the alignment properties of the 50 ONT reads mapping to 19 structurally intact Copia-23 loci whose INT domain was >90% covered by ONT reads in Vv_Mock12h. For each read, the read length was plotted against the number of bases overlapping with the autonomous locus and coloured by mapping specificity (multi-/unique-mapping; A), as well as alignment start site (B) and end site (C) relative to the TE locus. Therefore, all reads plotted in (A) were presented at the same coordinates in (B) and (C). **(D-O)** Scatter plots demonstrating the alignment properties of the ONT reads mapping to structurally autonomous Copia-3, VLINE7, and VLINE8 loci whose INT or annotated feature (for LINE element) was >90% covered by ONT reads. The experimental conditions were as indicated. (D)-(O) were plot following the approach used for (A)-(C).
