# Supplementary material for: Comprehensive analysis of both long and short read transcriptomes of a clonal and a seed-propagated model species reveal the prerequisites for transcriptional activation of autonomous and non-autonomous transposons in plants
Source: Mob DNA. 2022 May 12;13:16. doi: 10.1186/s13100-022-00271-5 (PMC9097378; doi:10.1186/s13100-022-00271-5)
Supplement: Supplementary file 8 — Additional file 8. Supplementary methods. [file 13100_2022_271_MOESM8_ESM.docx]

# **Additional file 8: Supplementary methods**

## Stress treatment

Every half gram of solid embryogenic callus cultures (*V. vinifera* cv Pinor noir clone UCD5) was collected into a 15-mL Corning conical polypropylene centrifuge tube (Sigma) containing 12 mL of hormone-free C_1_^P^ liquid medium (HF- C_1_^P^) that have been supplied with either live *H. uvarum* cultures re-suspended to OD600 = 0.8 as described in Lizamore (2013) [23]. The embryogenic callus cultures exposed to stressors were subjected to vigorous shaking for 2 seconds manually, following with incubation on a rotary shaker (100 RPM) horizontally for 8 minutes at room temperature. These calli were then transferred and spread evenly on HF- C_1_^P^-soaked filter papers on fresh C_1_^P^ plates. Continuously with the presence of the biotic stressor, these plates were incubated at 25˚C in the dark. Preparation of *H. uvarum* yeast culture was carried out as described in Lizamore (2013) [23]. Mock experiments were identical to that carried out for treatments with live yeast cultures with the same volume of stressor-free HF-C_1_^P^ liquid medium. Each treatment was harvested at 1, 3, 6, and 12 hours as shown in Fig. 1a, and gently washed with 50mL of HF-C_1_^P^ three times before being snap-frozen with liquid nitrogen. A common untreated 0 hour time point (denoted as Vv_T=0) for mock and the biotic treatment was taken prior to any treatment. All treatments and their associated time points consisted of three technical replicates.

## Identification of TE expression candidates

This analysis is comprised of three sub-pipelines described as follows:

The first sub-pipeline only collects TEs obtaining unique-mapping reads that each of these sequencing reads can be traced back to a unique origin in the genome. As illustrated in Additional file 2: Figure S1, sequencing reads unmapped to grapevine’s tRNA and rRNA sequences were aligned to 12X PN40024 grapevine reference genome using HISAT2 [70] with the parameters: *–rna-stradness RF –dtk –k 100*. Unique-mapped reads mapping to TEs were then quantified by *htseq-count* of the package Htseq [42]. TEs having read count more than 10, which approximates to 5 pairs of read, were collected.

The concept of the second sub-pipeline was to collect individual TEs that were aligned with any kind of reads, irrespective of the number of highest quality mapping loci for a given read. Following read alignment using HISAT2 as described in sub-pipeline 1, the BEDtools suite [43] was used in read quantification (Additional file 2: Figure S1, sub-pipeline 2). The command *bedtools coverage* generated raw count for TEs while multi-mapping reads matching to n-places (e.g. 10) was recorded n-times (i.e. 10). It also counted the number of bases of a TE locus covered by reads (covered bases of TE). Furthermore, this sub-pipeline incorporated *bedtools intersect* to calculate the number of bases of a read overlapping with an individual TE (mapped bases of read), which were summed for each TE locus to estimate the average read depth of an individual TE’s mapped region (i.e. only the region covered by reads, not the entire annotated feature). This was calculated as follows:

For *n* reads mapping to a TE locus, and *i* as an integer from 0 to *n*, *f(i)* = mapped bases of read *i.*

Average read depth of an individual TE’s mapped region =$\frac{\sum_{i=0}^{n} f\left( i \right)}{Bases of a TE locus covered by reads}$ .

In order to exclude TE loci that were covered by reads in a sparse and scattered way, a cut-off threshold of the average read depth five was adopted in addition to the ten read count threshold. Examples of the filtering step of this sub-pipeline were illustrated in Additional file 2: Figure S1.

The third sub-pipeline specifically collects TEs had transcription across the boundaries of the element. The software TEFingerprint [44] was originally designed for identifying unannotated insertions in genomes using paired-end short fragment DNA sequence data. This research applied TEFingerprint to capture TE loci internally mapped by multi-mapping reads only, yet the read mates, known as danglers, were uniquely aligned to a location near the insertion site (Additional file 2: Figure S1). Although TEFingerprint also has the option to use reads sit across the junction of a TE locus, this function was disabled in sub-pipeline 3 as the utilization of *htseq-count* in sub-pipeline 1 has covered this scenario. Sub-pipeline 3 applied the standard TEFingerprint pipeline where reads were mapped against the collection of 223,411 annotated *V. vinifera* TE sequences using BWA [71]. Subsequently, the mates of TE-mapped reads were aligned to the reference genome (12X PN40024) before calculating the read count of dangler clusters. Only clusters containing more than 10 dangler reads were kept to test for the intersection of dangler clusters and annotated TEs using *bedtools intersect*. The candidates need to show more than 10 dangler reads and more than 10 reads mapping internally (counted by *bedtools coverage*).

After excluding TEs that did not show enough transcription, potentially expressed TEs from the three sub-pipelines were collected together as a pool of expression candidates.

Grapevine’s TE annotation was established by Lizamore (2013) [23]. In general, all canonical transposable element sequences of *V. vinifera* were downloaded from the Repbase Update database (<https://www.girinst.org/repbase/>; access in 2016). To reconstruct the full-length canonical element of LTR-retrotransposons (LTR-TEs), LTRs and the internal sequences were reassembled for each family in the format LTR-Internal-LTR. The canonical TE sequences were used to extract local copies of the 12X PN40024 grapevine genome using RepeatMasker [72] with the default setting. Simple sequence repeats (SSRs) were masked with N’s using RepeatMasker. In total, 232 canonical TE sequences, in which each sequence represents a TE family of *V. vinifera*, were used to extract 223,411 TE-like sequences of the 12X PN40024 genome.

## Profiling of TE expression candidates

***TE integrity analysis***

The length of each annotated TE locus was compared to the corresponding canonical element sequence retrieved from the Repbase update database and reconstructed as previously described. TE loci longer than 90% of the corresponding canonical elements in length were considered full-length elements, and the rest of the annotated loci were grouped as fragmented TEs.

***Identification of transcriptionally active TE family***

To generate figures like Fig. 2, expression candidates were initially grouped into trackable (collected from the sub-pipeline 1 and 3 in Fig. 1a) and un-trackable (captured only by the sub-pipeline 2 in Fig. 1a) expression candidates. All expression candidates were further binned by family and integrity. These information were then integrated into charts like Fig. 2, where, for each TE family (the y-axis), the accumulated number of un-trackable and trackable expression candidates are respectively plotted on the left and right sides of the chart; based on this layout, the accumulated number of full-length and fragmented loci is indicated by dark and light colours respectively.

***Cladogram analysis of full-length Copia-3 and Copia-23***

In the analysis mentioned earlier, Copia-3 and Copia-23 were found to be the two TE families representing the most un-trackable full-length expression candidates, suggesting that these two TE families retain a substantial number of highly similar or identical full-length loci that are indicative of their recent activity in the evolutionary time. To investigate how diverse are these full-length TE loci, multiple alignments of the canonical sequences and full-length TE loci of Copia-3 and Copia-23 was performed by Geneious using MUSCLE alignment [73] option with default settings. The Neighbour-Joining consensus tree was then constructed by Geneious with 100 bootstraps and a 90% support threshold. The tree was further illustrated by iTOL (Interactive Tree of Life, <https://itol.embl.de/>). All the analysed sequence here are labelled by five different colours (Additional file 2: Figure S3-S4) that represent five categories of these full-length elements. These five categories are: structurally autonomous (full-length and flanked by LTRs) untrackable expression candidates; structurally autonomous trackable expression candidates; structurally non-autonomous (full-length but lost at least one LTR) un-trackable expression candidates; structurally non-autonomous trackable expression candidates; non-expressed full-length loci.

Note that in this analysis, LTR-TE loci that are full-length and flanked by LTRs are denoted as ‘structurally autonomous’ loci, while LTR-TE loci that are full-length but not flanked by LTRs are denoted as ‘structurally non-autonomous’ loci for convenience. Although there are other sequence compartments that are also determinant to the autonomous mobilization of LTR-TEs, such as primer-binding sites as well as gag and pol genes encoding proteins required for self-competent transposition, the selection of LTR-TE loci that are full-length in sequence size and flanked by LTRs has efficiently excluded 78.4% and 72.6% of the total annotated Copia-3 and Copia-23 loci, respectively, leaving the rest 87 Copia-3 loci and 177 Coipia-23 loci considered as structurally autonomous loci. The polymorphisms that accumulated in sequence context but did not greatly affect sequence size were then measured during sequence alignment by MUSCLE [73], and we took this into consideration in the construction of the Neighbour-Joining consensus tree.

***Analysis of reads mapping to Copia-3 and Copia-23***

To test whether reads mapping to Copia-3 and Copia-23 expression candidates were all derived from fragmented un-trackable expression candidates instead of full-length un-trackable expression candidates that share similarity with the fragmented counterparts, these reads were collected using *bedtools intersect* of the package Bedtools [43] and analysed as follows: reads from the triplicates of same time point (see the experimental design in section 2.3.1) were merged and then categorized into four groups by their mapping destinations: fragmented un-trackable expression candidates; full-length un-trackable expression candidates; fragmented trackable expression candidates; full-length trackable expression candidates. This gave four groups of reads for plotting Venn diagrams using the R package VennDiagram [74]. Note that, as presented in the Venn diagrams in Additional file 2: Figure S5-S6, a multi-mapping read may be categorised into multiple categories, including the categories of trackable expression candidates for that part of the DNA sequences of these trackable expression candidates may be identical to a portion of the DNA sequences of the un-trackable expression candidates.

***LTR domain annotation***

To identify full-length LTR-TE loci retaining LTRs and estimate the insertion date of these loci (the following section), the coordinates of LTRs in the *V. vinifera* reference genome were extracted based on the LTR domain annotation that has been established by Lizamore [23]. Without reassembling the LTR-INT-LTR structure, TE element sequences of V. vinifera retrieved from the Repbase database were adopted directly for the LTR annotation following the workflow described in Lizamore [23]. This kept annotated LTR and INT domains separated. The previous annotation version generated using reconstructed LTR-INT-LTR canonical sequences was compared with this version (LTR and INT separated) of annotation to identify local copies of LTR-retrotransposons (LTR-TEs) flanked by LTRs on both ends and extract the coordinates of LTR domains.

***Estimation of LTR-TE insertion date***

Full-length TE loci flanked by LTRs were considered intact copies. The insertion time of these TE loci was dated by measuring the divergence between the 5’ and 3’ LTR for each locus, as proposed by SanMiguel et al. [47]. With the coordinates of INT domains in the reference genome, the sequences of paired LTRs were extracted using BEDtools’ function *bedtools getfasta* [43] and aligned by MUSCLE [73] with the settings *-distance1 kmer4_6 –clwstrict*. Following Vitte et al. [75], the observed divergence was corrected according to the Jukes-Cantor sequence evolution model [76] with the formula:

$$K=-\frac{3}{4}\ln(1-\frac{4}{3}p)$$

where *K* is the corrected divergence and *p* is the proportion of different bases in the two LTR sequences. The insertion date was then translated from the corrected divergence with an average substitution rate of 6.5×10^-9^ substitution per site per year estimated from the *Adh1* and *Adh2* genes of grass species including maize, rice and barley [77] and had been adopted by Moisy et al. [78] for the grapevine genome. The insertion time distribution was plotted by ggplot2 [79] and depicted the insertion history of LTR-TE families with at least 10 intact copies. The peak mobilization was estimated from the distribution of insertion times using R package hdrcde [80]. To test whether the LTR-TE families, Copia-3 and Copia-23, stacked with un-trackable full-length candidates were more recently active than others, their peak insertion dates were compared with other 5 LTR-TE families with trackable full-length candidates and a statistical test (t-test) of the mean insertion times was pair-wisely performed in a pair-wise manner with the function t-test in R.

***Location bias analysis***

The location of annotated TEs in association with genes was analysed using BEDtools’ function *bedtools intersect* [43]. The intersects between TEs and gene-related features, including exon, intron, 2kb upstream (N-flank) and 2kb downstream of any given gene locus, were further examined using R script. If a TE overlapping with an exon and intron has over 95% of its body covered by the intron, it would be assigned as intronic TE, and otherwise, it would be assigned with TE loci overlapping with exon. This rule was also applied to a TE overlapping with an exon and flanking region. All annotated TEs and expression candidates were categorized with R package dplyr [81] and plotted layer by layer as a pie graph using ggplot2 [79]. The goodness of fit X-square test was performed using the function *chisq.test* implemented in the R package MASS [82].

## Differential expression analysis

In the grapevine system, TE expression candidates with unique-mapping reads (i.e. trackable TE expression candidates) in at least one of the experimental conditions were collected for differential analysis of the expression dynamic changes under different treatments over time. For those captured by sub-pipeline 1 (Additional file 2: Figure S1), their raw read count generated by Htseq’s tool *htseq-count* [42] was used for the analysis. The rest of the trackable expression candidates collected from sub-pipeline 3 (Additional file 2: Figure S1) were analysed using the raw read count of danglers produced by TEFingerprint [44]. The analysis was conducted using DESeq2 [50] as read counts of the trackable candidates in all treatments were analysed together with multifactor settings, where the full model of the design formula included treatments and time points and the reduced model included the factor of time only. Raw read counts of TEs with adjusted p-value under 0.05 were normalized and logarithmically transformed and normalized using the function *varianceStablizingTransformation* (VST) implemented in DESeq2 [50]. Afterwards, the VST-transformed read counts in mock treatment (Vv_Mock) were normalized against Vv_T=0, while the VST-transformed read counts in yeast treatment (Vv_Yeast) were normalized with that of Vv_T=0 and then the effect from mock was further deducted at each time point. After these processes, TEs with the final normalized values above 1 in at least one time point were considered differentially expressed TEs (DETEs). The Venn diagrams showing the unique and shared DETEs among the three stress treatments were generated using the R package VennDiagram [74]. Hierarchical clustering was conducted using *hclust* in R with the Pearson correlation method for measuring distances among DETEs and the ‘*complete*’ agglomeration method for clustering. With the hierarchical clustering information, heatmaps of the final normalized value were plotted by *heatmap.2* from the package gplots [83]. For each DETE cluster, the expression trend revealed from the heatmap was illustrated with a simplified line graph. The statistics of the expression trend were summarized as a pie graph using ggplot2 [79]. Differential analysis and expression pattern clustering for genes were conducted in the same way as for TEs. Co-localized DETE and DEGs were gathered for hierarchical clustering as mentioned above to test whether the paired DETE and DEG would be grouped into the same expression cluster. Note that a small number of DETEs, especially DETEs within 2kb flanking regions of genes, might co-localise with multiple DEGs and *vice versa*. Instead of arbitrarily excluding DETEs or DEGs fallen into this scenario, the comparison of the expression pattern of co-localized DETEs and DEGs was conducted on each DETE-DEG pair.

## Alternative-splicing analysis

The tool *flair diffSplice* embedded in FLAIR pipeline [53] was used to group alternative splicing into four types, including alternative 5’ splicing (Alt5), alternative 3’ splicing (Alt3), intron retention (IR) and exon skipping (ES). In addition, the productivity of each isoform was estimated by FLAIR’s *predictProductivity*, which predicts four types of productivity, including productive isoform (PRO), presence of premature termination codon (PTC), absence of start codon (NGO) and absence of stop codon (NST). To further capture alternative splicing feature directly related to TEs, BEDtools’ *bedtools intersect* was applied to identify alternative splicing features overlapping with annotated TE loci. These features would be denoted as TE-associated or TE-related alternative splicing features.

## Identification of the potential origin of full-length transcription for autonomous TE loci

For LTR-TEs, the transcription initiates from within the 5’ LTR and progresses through the primer binding site (PBS), internal domain (INT) encoding proteins necessary for autonomous transposition, as well as polypurine tract (PPT), and termination at the 3’ LTR. Therefore, as shown in Additional file 2: Figure S22, the workflow for selecting putative autonomous LTR-TE candidates exhibiting potential competent transcription began with selecting full-length TEs showing more than 90% of length coverage relative to the corresponding canonical TE sequence. Secondly, full-length candidates were examined for the presence of a pair of LTRs. Lastly, using BEDtools’ *bedtools coverage* [43], only candidates with > 90% breadth of coverage over the INT domain would be qualified as autonomous expression candidates.

Autonomous type I non-LTR retrotransposons (non-LTR-TEs) were mostly LINE elements possessing 5’ and 3’ untranslated regions (UTR) and an open reading frame (ORF) encoding poly-protein that can be processed into a protease (PR), reverse transcriptase (RT) and RNase H (Additional file 2: Figure S23). The transcription initiating from the 5’ UTR throughout the elements is necessary for reverse transcription and autonomous transposition. Therefore, full-length expression candidates of non-LTR-TEs were initially collected, following by a selection for those originated from non-LTR-TE families retaining intact RT domain with putative active sites. Those with over 90% breadth of coverage throughout the elements were considered autonomous expression candidates.

Type II expression candidates are typically flanked by terminal inverted repeats (TIRs) and contain an ORF that encodes a transposase (TPase) (Additional file 2: Figure S24). The TPase protein is assembled with multiple functional domains, including DNA binding and TPase catalytic cores, as well as other domain for metal ion chelating or dimerization depending on the TE families, such as hATC (*hAT* C-terminal dimerization) domain for *hAT* and SWIM (SWI2/SNF2 and *MuDR*) for *MULE* [4]. However, for some of these TE families in grapevine, the TIRs structure has been missing in the canonical element sequences, leaving them incompetent for autonomous mobilization. Therefore, only full-length elements belonging to the competent TIR-TE families were included in this study. The coordinates of the ORF encoding TPase (TPase-ORF) were extracted by ORFfinder (<https://www.ncbi.nlm.nih.gov/orffinder/>) and used as input into BEDtools’ *bedtools coverage* [43] to analyze the breadth of coverage of TPase-ORF. Those showing >90% coverage of the ORF were considered as autonomous expression candidates of TIR-TEs.

With the consecutive transcript information provided by the ONT platform, the aforementioned potential loci were further examined by the presence of ONT read covering the TE feature or domain necessary for autonomous mobilization. Among these ONT reads, the recognition of unique- and multi-mapping reads was performed by ‘home-brew’ scripts (<https://github.com/ting-hsuan-chen/TE_ExpressionCandidate>) that counted the number of entries in the SAM file for each TE-overlapping read since equally good alignments would be randomly designated as primary or secondary alignment by minimap2 [85], and minimap2 doesn’t report SAM field tag regarding the alignment uniqueness.

This analysis was visualized by plotting the length of ONT reads mapping to the autonomous TE loci against the number of read-bases overlapping with TEs (Fig. 6a,d; Additional file 2: Figure S25). This is to select TE loci fully covered by individual reads spanning across the necessary regions instead of those covered by co-contribution of multiple ONT reads. These reads were also surveyed for their transcriptional start and stop sites relative to mapped TE loci. BEDtools’ *bedtools intersect* [43] was used to distinguish ONT reads started or ended internally or externally. For those internal reads, if there were more than 10 clipped bases, these starts or ends would be denoted as clipped in Fig. 6 and Additional file 2: Figure S25. The genome browse image of qualified TE loci was generated by a local instance of JBrowse v1.16.8 [87].
